# Online education for rare genetic diseases: a systematic review

**DOI:** 10.1186/s13023-025-03809-x

**Published:** 2025-12-31

**Authors:** Pinar Ozmizrak, Luigi Boccuto, Tracy Brock Lowe, Stephanie Trammel, Jane DeLuca

**Affiliations:** Clemson University School of Nursing, Clemson, SC 29634 USA

**Keywords:** Rare disease, Online education, Genetics, Healthcare, Open educational resources

## Abstract

**Introduction:**

Rare genetic diseases are, collectively, not in fact rare. However, educational opportunities focused on rare genetic disease can be limited. The Internet has increased the availability of education related to rare genetic disease and is accessible to a diverse range of people who seek out such information, including healthcare professionals, researchers, students, patients, and the public.

**Purpose:**

To assess the potential educational outreach of the Internet, this systematic literature review will appraise the landscape of what education for rare genetic disease is available online, describing its form, subject, and intended audience.

**Methods:**

This systematic review encompassed all results across 20 science, healthcare, and education databases published up to September 1, 2023. The search criteria were specific to online education for rare genetic diseases.

**Results:**

From 1663 total results, after applying exclusion criteria, 58 publications remained, ranging from 2002 to 2023. Although the amount of research presenting rare genetic disease education online was limited, the forms of education and its target learners were varied. Studies could have multiple target learners and healthcare professionals (68.97% of papers) and healthcare consumers (62.07% of papers) represented the most common of 5 different learners. 22 different specific conditions or categories of disease were the focus of 56.90% papers, with the remainder being general subjects like ‘genetic testing’ or ‘rare diseases’ overall. Modes of delivery were mutually exclusive per paper, with websites (29.31% of papers) and web applications/modules (24.14% of papers) being the most common of 7 different forms. The highest representation for author institutions was the USA (58.62% of papers) out of 33 countries total. The broad spread of learners, subjects, and delivery forms demonstrates the potential for online education as a vehicle for advancing the reach of rare disease education.

**Conclusions:**

The greater accessibility afforded through online information creates an avenue for further availability of high-quality education on rare genetic diseases.

**Supplementary Information:**

The online version contains supplementary material available at 10.1186/s13023-025-03809-x.

## Introduction

Many disciplines can be taught through online education. Although the field of online education saw a sharp growth during the COVID-19 pandemic as an emergency measure to continue education delivery at a distance [1], online education has existed for far longer, being first introduced in 1989 as a consumer service [2].

Delivering education through the Internet extends accessibility to learners, diverse in both their location around the world and their educational background. Online delivery gives genetics education reach to healthcare professionals and the public alike. Genetics, in particular, is a subject that is of high importance to learners of varied medical and non-medical backgrounds, such as the healthcare stakeholders of patients, healthcare professionals, healthcare institutions, payors, and policymakers [3].

A domain within genetics is rare genetic diseases, many of which can be severe, chronic, and have early onset. These may also be called orphan diseases due to their scarcity of research, with less than 6% of conditions having an approved treatment option [4]. “Rare” can be defined through different metrics, such as affecting fewer than 200,000 in the USA or under 1 in 2000 people in the European Union [5]. However, rare diseases are collectively common, estimated to affect 1 in 17 people in their lifetime [6]. The combination of severe, life-long conditions with limited research due to individual rarity but a high collective frequency make rare disease education critical for addressing this research gap.

The purpose of this systematic review was to describe the literature devoted to published online education in healthcare relating to rare genetic diseases and address the following research questions (RQ):

*RQ1*: Who is online education for rare genetic diseases designed for?

*RQ2*: What topics are the focus of online education for rare genetic diseases?

*RQ3*: How is online education for rare genetic diseases delivered?

The PROSPERO international prospective register of systematic reviews was consulted to ensure this systematic review would not duplicate another. A combination of searches using the terms “online genetics education” revealed only 1 result, an ongoing review which differs in focus from this review [7]. The review is specific to primary care professionals as learners, broad to genetic subject, and broad to form of educational delivery while this review is broad to learners, specific to rare disease as a subject, and specific to an online form of educational delivery.

Similarly, other reviews that have been performed in genetics education are specific to only healthcare professionals as learners, and not specific to rare disease or online delivery [8–10]. As such, the scope of online education for rare genetic diseases is sufficiently unique to warrant a systematic review.

## Methods

The literature search had no starting limit and extended to publication dates up to September 1, 2023. The search strategy included five necessary categories, with synonyms within each category as shown in Table 1. Academic publications and English language were further requirements for the literature.

**Table 1:** Search strategy for systematic review

Category	Search terms
Online-related	(online OR Internet OR virtual OR web OR website OR open-access)
Education or learning resources	(educat* OR learning OR teaching OR training OR course OR program OR module OR modular OR tutorial)
Genetic or genomic	(gene OR genetic* OR genom*)
Disease-related	(health* OR disease OR disorder OR condition OR syndrome)
Rare condition	(rare OR orphan*)

Combined, the search terms queried in all databases were written as follows:

(online OR Internet OR virtual OR web OR website OR open-access) AND (educat* OR learning OR teaching OR training OR course OR program OR module OR modular OR tutorial) AND (gene OR genetic* OR genom*) AND (health* OR disease OR disorder OR condition OR syndrome) AND (rare OR orphan*)

The 20 databases and search fields queried for each are listed in Table 2.

**Table 2:** Fields searched within each database

Database	Search Field
PubMed	All fields (not full-text)
Web of Science Core Collection	Topic (abstract, keywords)
EBSCO • Academic Search Complete • APA PsycInfo • CINAHL Plus with Full Text • Cochrane Central Register of Controlled Trials • Cochrane Database of Systematic Reviews • Consumer Health Complete—EBSCOhost • Consumer Health Reference eBook Collection • Education Full Text (H.W. Wilson) • Education Research Complete • ERIC • Health Source—Consumer Edition • Health Source: Nursing/Academic Edition • MEDLINE • OpenDissertations • Psychology and Behavioral Sciences Collection • SPORTDiscus	Abstract
Gale OneFile: Educator’s Reference Complete	Keyword (key fields, not full-text)
CAB Direct	All fields (not full-text)

The chosen fields were selected specific to each database for consistent search parameters between the databases. For example, some databases offered full-text searches while others did not. The selected search fields for each database allowed for an approximately consistent search of key terms appearing within the title or abstract of the records.

## Results

Across the 20 databases searched, there were 1663 total results retrieved, as shown in Table 3. Amongst these results, there were 578 duplicate records, leaving 1085 unique records. The full list of unique records is available in Supplement 1.

**Table 3:** Database results as of September 1, 2023

Database	Results
PubMed	818
Web of Science Core Collection	339
EBSCO (16 databases)	414
Gale OneFile: Educator’s Reference Complete	30
CAB Direct	62

**Fig. 1:**
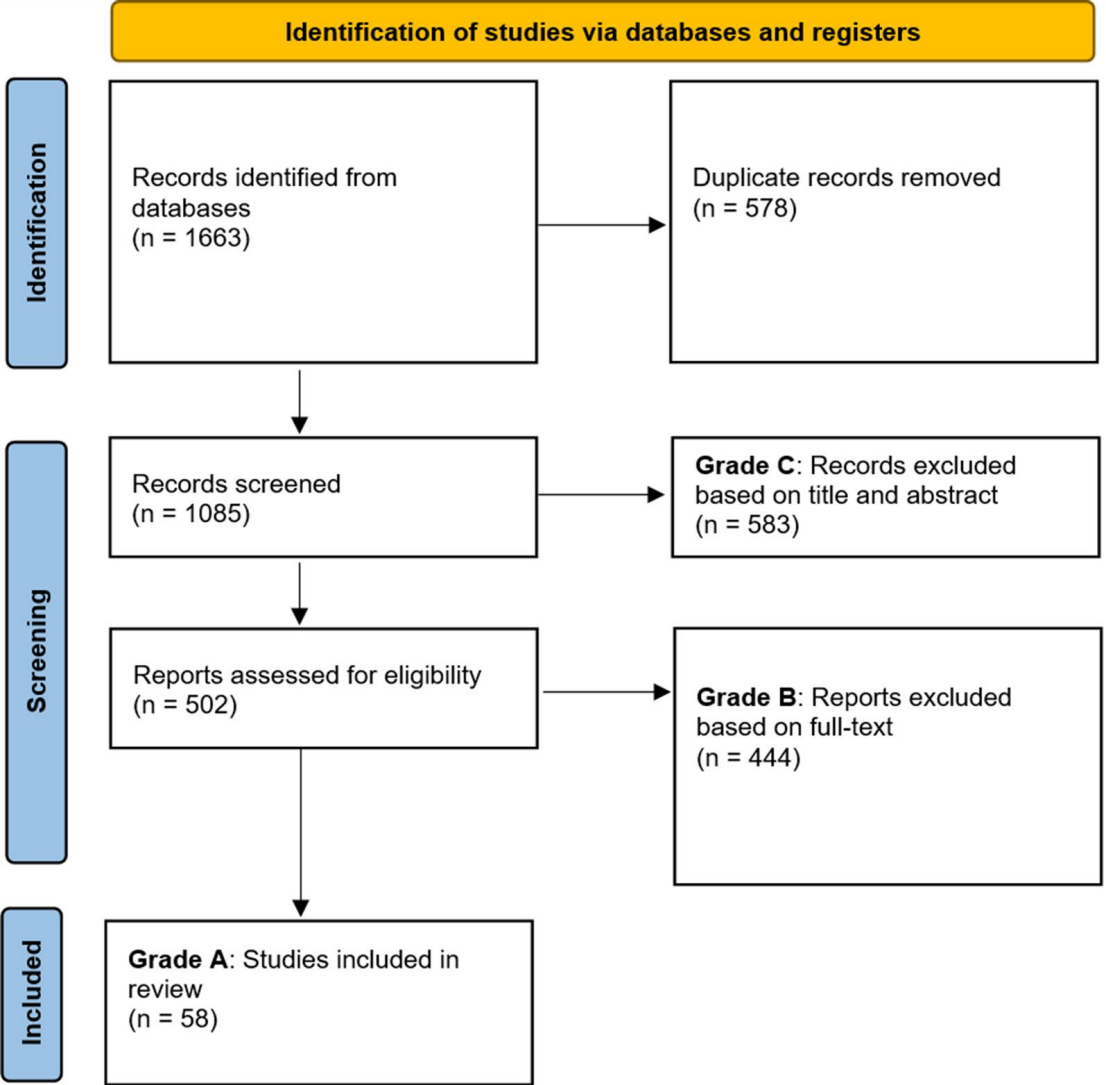
PRISMA 2020 flow diagram

**Fig. 2:**
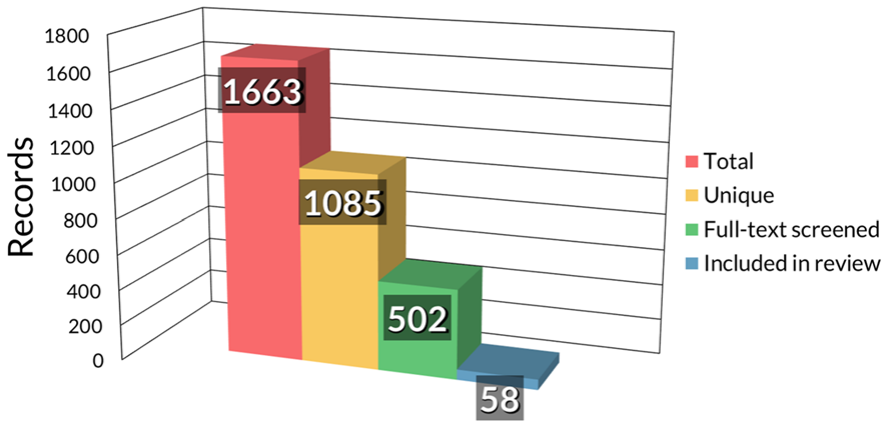
Clustered column chart of records

Figure 1 depicts a Preferred Reporting Items for Systematic Reviews and Meta-Analyses (PRISMA) diagram of the paper selection process [11] and Fig. 2 illustrates the number of records from the total search to what was included in the review.

Although all records contained all five required categories of keywords (synonyms for rare genetic disease online education), the combination of keywords could result in a variety of topics not relevant to online education for rare genetic diseases. An ABC grading system was used to screen results, where C was the least relevant to the scope and A the most relevant, based on how many of the five criteria of online, education, rare, genetic, and disease the result was related to.

### Grade C

Grade C results constituted those that, from the title and abstract, were not related to online education for rare genetic diseases. Of the 1085 unique records, 583 were graded as C results and excluded from further screening. There were multiple entries that used the key term “learning” but in the context of machine learning or deep learning, not education. Often, studies would not be related to online delivery, but included phrases such as “the online version of this article.” Database names such as Web of Science and Online Mendelian Inheritance in Man also resulted in a number of studies that did not have a digital focus but appeared because of inclusion of the terms “web” or “online.”

### Grade B

Grade B results were missing criteria to be online education for rare genetic diseases. Of the remaining 502 records, upon screening through the full-text, 444 were graded as B results. However, grade B were adjacently related, often missing one of the five critical components. For example, being an online resource for rare genetic disease but not being in the form of education. In some cases, education for rare genetic disease did not have an online mode of delivery. Grade B results were categorized based on their reason for exclusion.

The results that were graded as B and excluded from the final review are categorized in Table 4.

**Table 4:** Categories of grade B results (excluded from review)

Category	Results
Bioinformatics tool/resource	119
Clinical intervention	14
Crowdsourcing	3
Database/registry/network	98
Delphi consensus/guidelines	26
Knowledge assessment	10
Matchmaking	9
Non-academic literature	10
Non-English	12
Resource list	15
Social media/web search	22
Survey/interview	102
Telemedicine	4

The most common categories with grade B were papers presenting bioinformatics tools or resources; a survey or interview; or presenting a database, registry, or network website. Combined, these categories represented 71.85% (319/444) of all grade B results.

### Grade A

Grade A results met all five criteria for inclusion. There were 58 records graded as A and all were screened at the full-text level. These results represented records that were related to online education for rare genetic diseases. Table 5 represents all results included in the review with the number of citations current up to September 2023 and collected through Google Scholar. For each result, research questions 1, 2, and 3 are addressed through audience, topic, and delivery, respectively.

**Table 5:** A summary of grade A results of the review [12–69]

References #	Country	Audience	Topic	Delivery	Citations
[12]	USA, Canada	Healthcare professionals, researchers, healthcare consumers	Genetic testing	Website	79
[13]	France, Germany	Healthcare professionals, researchers, healthcare consumers	Rare diseases	Website	158
[14]	Canada	Healthcare professionals	Mucopolysaccharidosis type II (Hunter disease)	Web application/module	21
[15]	USA	University students	Genetic testing	Virtual laboratory/case study	21
[16]	Spain	Healthcare professionals, researchers, healthcare consumers	Inherited metabolic disorders	Website	3
[17]	USA	Healthcare consumers	Rare diseases	Website	0
[18]	USA	Healthcare consumers, K-12 students	Rare diseases	Website	0
[19]	USA	Healthcare professionals, researchers, healthcare consumers	Duchenne muscular dystrophy	Website	11
[20]	USA	K-12 students	Complex diseases	Web application/module	9
[21]	USA	Healthcare consumers	Mitochondrial diseases	Electronic document	0
[22]	Italy	Healthcare professionals, researchers	Fabry disease	Website	5
[23]	Romania, Italy, Russia, Belgium, Switzerland, Israel, Hungary, Denmark, UK, Bulgaria, Spain, Portugal, Cyprus, Poland, Serbia, Slovenia, France	Healthcare professionals, researchers, healthcare consumers	Congenital hypogonadotropic hypogonadism and Kallmann syndrome	Electronic document	31
[24]	Ireland	Healthcare professionals, researchers, healthcare consumers	Rare diseases	Website	0
[25]	USA	Healthcare professionals, researchers, healthcare consumers	Rare diseases	Website	29
[26]	USA	Healthcare professionals	Rare diseases	Website	3
[27]	USA	Healthcare professionals, researchers, healthcare consumers	Inherited metabolic disorders	Website	11
[28]	USA	Healthcare professionals, researchers	Rare diseases	Web application/module	1
[29]	USA	Healthcare consumers	Breast cancer	Web application/module	0
[30]	USA	Healthcare consumers	Genetic testing	Online course	16
[31]	Germany	Healthcare professionals, researchers	Movement disorders	Video	6
[32]	USA	Healthcare consumers	Alpha-1 antitrypsin deficiency	Website	2
[33]	USA	Healthcare consumers	Genetic testing	Web application/module	10
[34]	USA	Healthcare professionals	Spinal muscular atrophy	Virtual laboratory/case study	0
[35]	France, Netherlands	Healthcare professionals, healthcare consumers	Rare diseases	Electronic document	29
[36]	Netherlands	Healthcare professionals, healthcare consumers	Male breast cancer	Website	29
[37]	Canada, USA, France	University students	Rare diseases	Web application/module	2
[38]	UK	K-12 students	Rare diseases	Video	7
[39]	UK	Healthcare professionals, researchers, healthcare consumers	Ehlers-Danlos syndromes and hypermobility spectrum disorders	Website	12
[40]	Australia, UK	Healthcare consumers	Genetic testing	Web application/module	27
[41]	USA	Healthcare consumers	Genetic testing	Web application/module	3
[42]	UK, USA	Healthcare professionals, researchers, healthcare consumers	Rare diseases	Online conference/webinar	0
[43]	USA	Healthcare professionals, researchers, healthcare consumers	Cystic fibrosis	Online course	2
[44]	USA, Spain	Healthcare professionals, researchers, healthcare consumers	Progeria	Online conference/webinar	4
[45]	Canada, Germany, Denmark, USA, Norway, Australia	Healthcare professionals	Genetic testing	Web application/module	49
[46]	USA, Italy, Saudi Arabia, Netherlands, Canada, UK, France, Belgium, Australia, Ireland, Switzerland, Poland	Healthcare professionals, researchers	Thrombosis and hemostasis	Online conference/webinar	4
[47]	Australia	Healthcare consumers	Eosinophilic gastrointestinal disorders	Online course	4
[48]	USA	Healthcare consumers	Genetic testing	Web application/module	11
[49]	Germany, France, Italy, Netherlands, Spain, New Zealand, Hungary, Belgium, UK	Healthcare professionals, researchers, healthcare consumers	Rare neurological diseases	Website	26
[50]	Netherlands, Greece	Healthcare professionals, researchers, healthcare consumers	Duchenne muscular dystrophy	Online conference/webinar	4
[51]	Germany	Healthcare professionals	Rare neurological diseases	Online conference/webinar	1
[52]	Italy	Healthcare professionals	Rare diseases	Web application/module	0
[53]	USA	Healthcare professionals, researchers	Rare diseases	Online conference/webinar	8
[54]	USA, Canada, Germany, Switzerland, France, Austria	Healthcare professionals, researchers, healthcare consumers	NUT carcinoma	Online conference/webinar	9
[55]	Switzerland, UK, France, Belgium, Netherlands, Italy, Ireland, Spain, Germany, Greece	Healthcare professionals, researchers	Interstitial lung disease	Online conference/webinar	0
[56]	India, USA	Healthcare professionals, researchers, healthcare consumers	Rare diseases	Online conference/webinar	0
[57]	Australia	Healthcare consumers	Developmental and epileptic encephalopathies	Web application/module	1
[58]	Germany, Canada, USA	Healthcare consumers	Lymphangioleiomyomatosis	Video	6
[59]	USA	Healthcare professionals	Thyroid cancer	Video	0
[60]	Romania, Austria, Germany, Finland, Portugal, UK, Italy, Switzerland	Healthcare professionals	Rare diseases	Website	1
[61]	Australia	Healthcare professional, healthcare consumers	Adult-onset diseases	Web application/module	0
[62]	Singapore, Portugal, USA, Germany, France	Healthcare professionals, researchers, healthcare consumers	Rare bone diseases	Online conference/webinar	0
[63]	Italy, France	Healthcare professionals, researchers	Rare neuromuscular diseases	Online course	0
[64]	USA, Sweden, Japan	Healthcare professionals, researchers	Skeletal dysplasia	Online course	0
[65]	Bulgaria, Finland, Netherlands, Germany	Healthcare professionals, researchers, healthcare consumers	Rare endocrine conditions	Online conference/webinar	0
[66]	USA	Healthcare professionals, researchers, healthcare consumers	Rare diseases	Website	1
[67]	Australia	Healthcare professionals, researchers	Children’s interstitial lung disease	Online conference/webinar	0
[68]	Canada, USA	University students, K-12 students	Von Hippel-Lindau disease	Web application/module	1
[69]	UK, USA	Healthcare professionals, researchers, healthcare consumers	Rare diseases	Online course	0

### Bibliometrics

The grade A results have a publication range of 21 years from 2002 to 2023. The countries of all authors’ institutions were collected and range across 33 countries (Australia, Austria, Belgium, Bulgaria, Canada, Cyprus, Denmark, Finland, France, Germany, Greece, Hungary, India, Ireland, Israel, Italy, Japan, Netherlands, New Zealand, Norway, Poland, Portugal, Romania, Russia, Saudi Arabia, Serbia, Singapore, Slovenia, Spain, Sweden, Switzerland, UK, and USA).

**Fig. 3:**
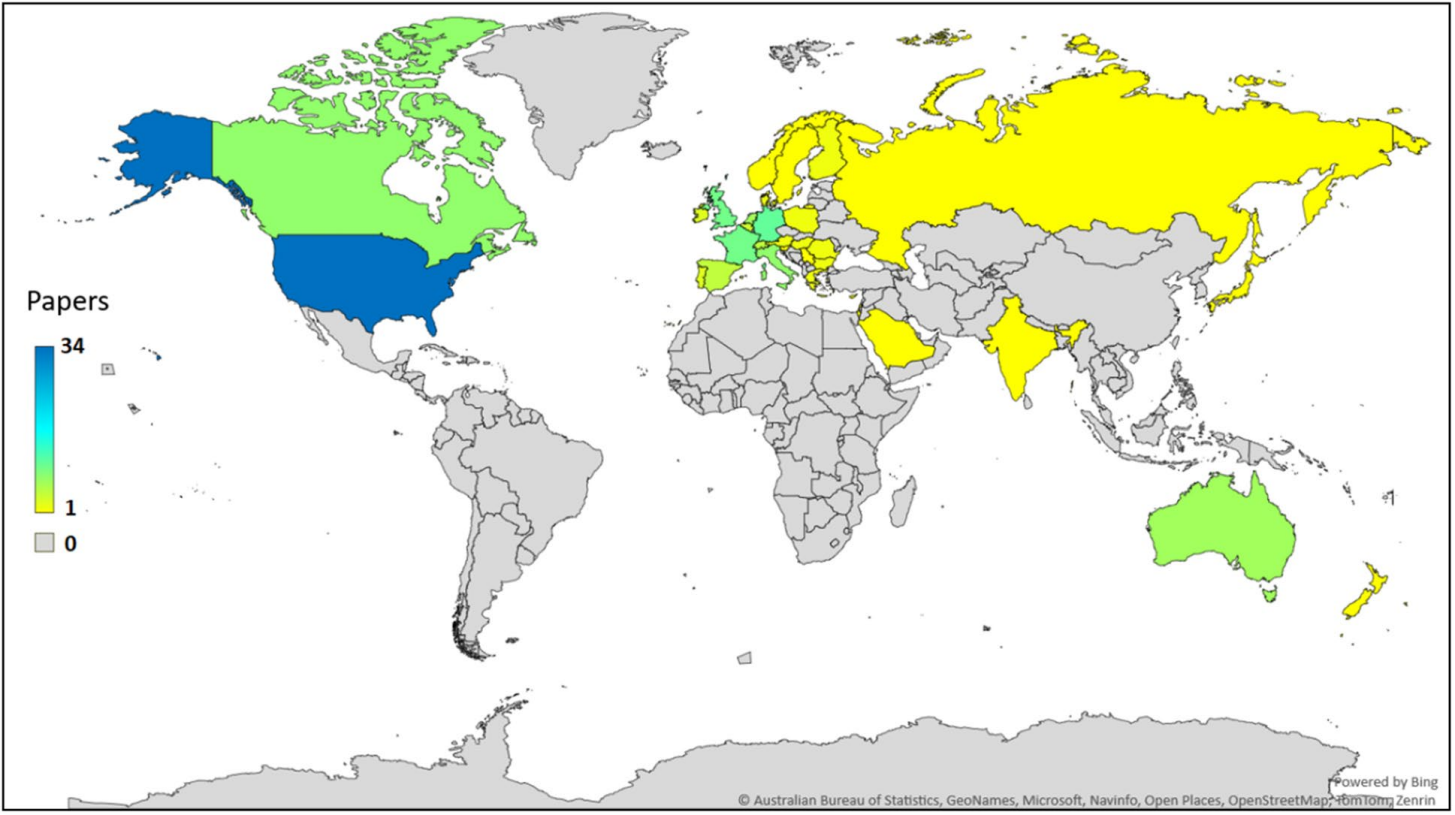
Map of countries of authors’ institutions represented in grade A results

The highest representation was author institutions in the USA, appearing in 58.62% (34/58) of papers. This component was not mutually exclusive; the studies could have authors from institutions across multiple countries. Figure 3 depicts a map of all represented countries, color-graded by number of results.

The 58 grade A results allow us to address the research questions as follows:

**RQ1: Who is online education for rare genetic diseases designed for?**

Across the studies, there were 5 different target learners categorized amongst healthcare professionals, researchers, university students, K-12 students, and healthcare consumers. This component was not mutually exclusive; the studies could have target learners across multiple categories.

Healthcare professionals included clinicians, nurses, genetic counselors, and other members of health professions. The category also included continuing medical education. Researchers included academics and non-clinical professionals. University students included students in healthcare professional programs such as medicine or nursing, as well as non-healthcare professional post-secondary students. K-12 students included children of school age up from elementary through high school. Healthcare consumers included patients affected by a genetic disorder, family of a patient affected by a genetic disorder, and laypersons or the public. The category also included patient education materials.

The highest represented target learner was healthcare professionals, appearing in 68.97% (40/58) of papers, followed by healthcare consumers at 62.07% (36/58), researchers at 50.00% (29/58), K-12 students at 6.90% (4/58), and university students at 5.17% (3/58).

**RQ2: What topics are the focus of online education for rare genetic diseases?**

Across the studies, the topics could be divided into two categories—general, representing a broad subject in the context of rare genetic disease, or specific, representing a particular rare genetic disease or category of diseases.

General subjects like ‘genetic testing’ or ‘rare diseases’ overall were the focus of 43.10% (25/58) of studies. Other studies offered more specialized education focusing on 22 different subjects across alpha-1 antitrypsin deficiency, breast cancer (male and female), cystic fibrosis, congenital hypogonadotropic hypogonadism & Kallmann syndrome, complex diseases, developmental & epileptic encephalopathies, Duchenne muscular dystrophy, Ehlers-Danlos syndromes & hypermobility spectrum disorders, eosinophilic gastrointestinal disorders, Fabry disease, inherited metabolic disorders, interstitial lung disease, lymphangioleiomyomatosis, mitochondrial diseases, mucopolysaccharidosis type II (Hunter disease), movement disorders, NUT carcinoma, rare bone diseases, spinal muscular atrophy, thrombosis & hemostasis, thyroid cancer, and Von Hippel-Lindau disease. This component was mutually exclusive; each study was represented by one primary subject of focus.

**RQ3: How is online education for rare genetic diseases delivered?**

Across the studies, there were 7 different forms of delivery including websites, web application/modules, virtual laboratory/case studies, electronic documents, online courses, videos, and online conference/webinars. This component was mutually exclusive; each study was represented by one mode of delivery.

The highest represented mode was through websites, appearing in 29.31% (17/58) of papers, followed by web application/modules at 24.14% (14/58), online conference/webinars at 20.69% (12/58), online courses at 10.34% (6/58), videos at 6.90% (4/58), electronic documents at 5.17% (3/58), and virtual laboratory/case studies at 3.45% (2/58).

## Discussion

All results varied among audience, subject, form, methods, and outcomes, showing a rich usage of interdisciplinary online education in genetic disease. This is a promising finding that there can be further education of rare genetic diseases online, integrating knowledge across disciplines and for diverse learners. The field currently has a wide breadth, so it can be improved through offering a greater depth of educative materials.

Although all grade A studies presented a form of education, some studies also performed educational research. This was defined through the reporting of outcome measures in the study. For example, Worst et al. presented an education video on thyroid cancer to healthcare professionals, specifically oncologists and endocrinologists [59]. Their study had a pre- and post-questionnaire, measuring the effect of education on knowledge and confidence, with results showing a statistically significant increase in both outcomes among the groups.

However, not all grade A studies included outcomes, though this information would be desirable as a measure of effectiveness. These studies were still included in the review because they presented online education for rare genetic diseases. As an example, Trinh et al. presented a video tutorial on how to use online tools to determine pathogenicity of variants in the context of movement disorders [31].

A key point of this review was to distinguish between articles which provided education rather than simply information. The Oxford English Dictionary defines information as “knowledge communicated concerning some particular fact, subject, or event”, whereas education is defined as “the systematic instruction, teaching, or training in various academic and non-academic subjects” [70, 71]. There were many results that presented *information* about rare genetic diseases online which were graded as B to distinguish them from the grade A results that presented *education*.

An example of a grade B result comes from Chen et al., who researched the use of social media in hemophilia care [72]. This study was pertinent to rare genetic disease in an online context; it was not a form of education being delivered, although it did include discussion of resources that could be educational. The study revealed social media to be of use in disseminating information for patient care, research, and advocacy purposes.

The 2002 study by Pagon et al. was the oldest study included in the review, presenting a previous form of the *GeneReviews* website which, when made accessible to the public, saw especially frequent use [12]. At the time, the website received 3,200 visits daily, approximating to 1.2 million visits annually, and currently it has reached over 7 million [73].

This importance of sharing information in a useable form was reflected in numerous studies on genetic information seeking on the Internet, the following of which were all captured as grade B results in this review. For example, Shalhub et al. found that amongst people with Vascular Ehlers-Danlos Syndrome, 62.3% listed the Internet as their most useful information source, followed by 18.4% listing a geneticist [74]. The Internet facilitates patient self-advocacy and can be lifesaving in helping speed diagnosis, as noted in case reports [75, 76].

In a survey performed by the European Joint Programme on Rare Diseases, the main targets for developing “an academic e-learning course on research topics in the field of rare diseases” were students (PhD or medicine), academic researchers, physicians, and patients, listed in descending order of targeting [77]. As observed among the grade A results, the most common target learner was towards healthcare professionals. This aligns with a grade B result, a survey of UK general practitioners where online modules were found to be the most preferred mode of delivering continuing genomic education, with a 70% selection [78]. Regarding length of online modules, most preferred a 30 min to 1 h timespan.

The grade A results were valuable both for being findings of presentations of online education for rare genetic diseases as well as providing insights for developing further such education. Consider the eClinic on Hunter disease by Al-Jasmi et al. [14]. The form of this module provided an eBook with expansive reference material paired with an interactive simulation of examining a patient with Hunter disease. It was designed for healthcare professionals, but gained use by the public, particularly patients and families with Hunter disease. The value of this education outside of the intended audience of healthcare professionals was that it was accessible and user-friendly, while also being a fount of knowledge from a credible source. As a result, the eClinic gained an international audience, and was translated into Japanese due to high interest.

The Hunter disease eClinic can serve as a model of accessible, informative, and engaging online education for rare genetic diseases. The structure is suitable for adaptation towards other diseases. As a limitation, the module was distributed through CD-ROM and a website running Flash, of which neither form is in common use today.

Thus, a consideration for online education for rare genetic diseases is the longevity of the education. This is dependent on the form the education is being delivered in and well as the potential to update or refresh the education as time goes on.

### Search strategy

A challenge in developing the search strategy for this review was to include relevant results while retrieving a manageable number of results to filter through. Online education can be described through varied terms. ‘Online’ and ‘web’ were critical key terms and required inclusion to capture comprehensive relevant results. However, they both increased the number of irrelevant results due to phrases such as “This article is available online” or listing of Web of Science or Online Mendelian Inheritance in Man in the abstracts.

‘Digital’ was not used as synonym for ‘online’ because apart from results already captured by other synonyms, it predominantly only added results related to digital in the anatomical sense.

The abstract of this study, if indexed in a database, would be retrieved by its own search strategy. However, a limitation of this strategy is that with inclusion criteria for five separate terms, even with multiple synonyms, it can exclude relevant results that are missing one or two components of the criteria explicitly written in the abstract. An example is a study by Staemmler et al. which assessed online resources on multiple myeloma for quality in patient education but was not retrieved in this search due to not describing the condition as ‘rare’ in the abstract [79].

### Conclusions and future research

Without the limiter of ‘rare’ or ‘orphan’ there are thousands more results returned (in PubMed alone, 13,565 compared to 818). Future studies can look to a broader landscape of online education for all genetics, without the specificity of rare disorders.

However, it is important to purposefully research rare disease and orphan drugs for a multitude of reasons. Conditions are rare individually, but collectively there are 7000 different rare diseases that affect approximately 300 million people in the world [5]. Also consider the frequent prevalence of carriers for rare genetic diseases. A condition with a 1 in 2500 incidence, such as cystic fibrosis, has a 1 in 25.51 carrier frequency, making 3.92% of the population carriers [80]. Along with having limited treatment research, rare genetic conditions are often severe in their detrimental effect on quality of life and life expectancy [4, 81]. These conditions vary in onset. They may occur from birth or in early infancy, or through adulthood and past reproductive age, potentially being inherited in future generations. Newer research and testing capabilities will identify more rare conditions with complex inheritance patterns. Rare disease can benefit greatly from further awareness and further research, such as studies in drug repurposing which can decrease time to market, lower costs, and increase drug success rates compared to novel orphan drug development [4].

This systematic review collected a variety of online education for rare diseases across target learners, subject matter, and forms of delivery. However, it revealed a scarcity of publications about educational opportunities. There are further forms of online education for rare diseases that are disseminated but not captured in this search, because the education itself has not been published or research about the education has not been performed and published.

An extension of this systematic review can be to analyze the authors of the studies. Although the authors’ countries of institutions have been examined, this can be furthered by observing the roles of the authors themselves to discover who was involved in developing the online genetics education, and how varied the authors were in terms of role (e.g. clinician, genetic counselor, rare disease advocate) and disciplinary focus (e.g. treatment research, patient education, advocacy).

The use of online education as a mode of delivery improves access to education for rare genetic diseases, a subject which both healthcare professionals and the public gain in learning from. Education facilitates awareness and research for rare diseases, providing invaluable benefit whose reach is far from rare.

### Post-review update

This systematic review covered literature up to September 1, 2023. A post-review update was performed in PubMed, with a full list of results available in Supplement 2. PubMed was the chosen database for the extended search due to having the highest coverage of results within the original search and providing publicly available user access. Using the same search method as in the original review, the update provided 277 new results in PubMed from September 1, 2023 to March 1, 2025. Using the same grading method as in the original review, 153 (55.23%) results were grade C—not relevant, 115 (41.52%) were grade B—missing criteria, and 9 (3.25%) were grade A—included in the post-review.

The 115 grade B results were applicable to labels under the same categories as within the original review (Table 6).

**Table 6:** Categories of grade B results of the post-review

Category	Results
Bioinformatics tool/resource	25
Clinical intervention	6
Database/registry/network	18
Delphi consensus/guidelines	11
Knowledge assessment	4
Matchmaking	2
Non-English	3
Resource list	2
Social media/web search	4
Survey/interview	32
Telemedicine	8

The extended search retrieved 9 new Grade A results on online education for rare genetic diseases that have been published since the systematic literature review (Table 7).

**Table 7:** A summary of grade A results of the post-review [82–90]

References #	Country	Audience	Topic	Delivery
[82]	France, Germany, Italy, Ireland	Healthcare professionals, researchers	Interstitial lung disease	Video
[83]	Canada, USA	Healthcare consumers	22q11.2 deletion syndrome	Electronic document
[84]	USA	Healthcare professionals, university students	Cystic fibrosis	Online course
[85]	France, Germany, Italy, Netherlands, Norway, Portugal, Spain	Healthcare professionals, researchers, healthcare consumers	Rare bone diseases	Website
[86]	UK	Healthcare professionals, healthcare consumers, university students	Rare diseases	Online course
[87]	Netherlands, South Africa, Sweden, Tanzania, USA	Researchers	Rare diseases	Online conference/webinar
[88]	Australia, Belgium, Canada, France, Germany, Italy, Netherlands, UK, USA	Healthcare professionals, researchers, healthcare consumers	RASopathies	Online conference/webinar
[89]	Brazil, France, Germany, Italy, Netherlands, Poland, Portugal, Spain, Sweden, Switzerland, Turkey, UK, USA	Healthcare professionals, researchers, healthcare consumers	LAMA2-muscular dystrophy	Online conference/webinar
[90]	USA	Healthcare professionals, university students	Kabuki syndrome and Noonan syndrome	Web application/module

The audience types and delivery methods in the post-review studies aligned with those found in the original review, introducing no new categories. New topics covered in the post-review include 22q11.2 deletion syndrome, RASopathies, LAMA2-muscular dystrophy, Kabuki syndrome, and Noonan syndrome.

From a bibliometric perspective, the new results included four author countries not previously represented in the review: Brazil, South Africa, Tanzania, and Turkey.

This systematic review identified studies on online education for rare genetic diseases as early as 2002 [12]. The number of these studies has increased in recent years, and with the inclusion of post-review results, 44 of 67 studies (65.67%) studies were published since 2020. While post-review results retained similar audience types and delivery modes, they introduced new topics in rare genetic disease education and extended the geographic diversity of author institutions. Combining the original review and post-review, the total results now include studies from across all continents except for Antarctica. Overall, online education for rare genetic diseases has been established across varied delivery methods and for varied audience types, and the field continues to grow with increasing speed, internationally, and featuring a wider range of rare disease topics.

## Supplementary Information

Additional file 1.

Additional file 2.

## Data Availability

The full list of search results and their grading for this systematic review is provided in the supplementary materials.
