# Supplementary material for: Online education for rare genetic diseases: a systematic review
Source: Orphanet J Rare Dis. 2025 Dec 31;21:42. doi: 10.1186/s13023-025-03809-x (PMC12874925; doi:10.1186/s13023-025-03809-x)
Supplement: Supplementary file 1 — Additional file 1. [file 13023_2025_3809_MOESM1_ESM.pdf]

Supplementary Materials 1

Systematic Review Results Spreadsheet

#	Grade	Category	PMID	Title	Authors	Journal	Year
1	A	Education	11968082	GeneTests-GeneClinics: Genetic testing information for a growing audience	Pagon, RA; Tarcz	HUMAN MUTATI	2002
2	A	Education	18026888	Networking for rare diseases: a necessity for Europe	Aymé S, Schmidt	Bundesgesundhe	2007
3	A	Education	20973983	Hunter disease eClinic: interactive, computer-assisted, problem-based appro	Al-Jasmi F, Mold	BMC Med Educ	2010
4	A	Education	21811165	The virtual diagnostic laboratory: a new way of teaching undergraduate med	Bean LJ, Fridovic	Genet Med	2011
5	A	Education	22909005	Guiametabolica.org: empowerment through internet tools in inherited meta	Armayones M, V	Orphanet J Rare	2012
6	A	Education		Quality patient education materials on the web	Barbie E. Keiser	Online	2012
7	A	Education		Finding the GEMSS in your school	Ann Donoghue D	The Exceptional	2014
8	A	Education	26356412	What can Duchenne Connect teach us about treating Duchenne muscular dy	Wang RT, Nelson	Curr Opin Neurol	2015
9	A	Education	28163322	"Touching Triton": Building Student Understanding of Complex Disease Risk	Loftin M, East K,	Am Biol Teach	2016
10	A	Education		Assessment of online educational resources regarding mitochondrial disease	Blankenship, S		2017
11	A	Education	28946642	E-Learning for Rare Diseases: An Example Using Fabry Disease	Cimmaruta C, Lig	Int J Mol Sci	2017
12	A	Education	28320476	Developing and evaluating rare disease educational materials co-created by	COST Action BM	Orphanet J Rare	2017
13	A	Education		The Irish National Rare Disease Office (NRDO): A national step towards impro	Lambert, Debora	International Jou	2017
14	A	Education	29152459	Marking 15 years of the Genetic and Rare Diseases Information Center	Lewis J, Snyder M	Transl Sci Rare Di	2017
15	A	Education	28126729	Putting the Pieces Together: Clinically Relevant Genetic and Genomic Resour	Miller R, Khromy	Hosp Pediatr	2017
16	A	Education	28724394	Development of newborn screening connect (NBS connect): a self-reported p	Osara Y, Coakley	Orphanet J Rare	2017
17	A	Education		Tutorial: Rapidly Identifying Disease-associated Rare Variants using Annotati	Kotlar, AV; Wing	ACM-BCB'18: PRO	2018
18	A	Education		Genetic Education in BRCA Families - NCT03544983	Schwartz, MD	Cochrane Central	2018
19	A	Education	29402763	Engaging a Community for Rare Genetic Disease: Best Practices and Educatio	Ortiz RA, Witte S	Interact J Med Re	2018
20	A	Education	30868102	How Do I Confirm that a New Mutation is Pathogenic?	Trinh J, Tadic V,	Mov Disord Clin P	2018
21	A	Education		Understanding information sharing about rare diseases: an evaluation of the	Xun Zhu	Journal of Comm	2018
22	A	Education	30964585	Understanding the present and preparing for the future: Exploring the needs	East KM, Cochra	J Genet Couns	2019
23	A	Education		Diagnosis and Treatment of Spinal Muscular Atrophy: Online, Case-based Ed	Finnegan, T	ANNALS OF NEUR	2019
24	A	Education	31801983	"Patient Journeys": improving care by patient involvement	Bolz-Johnson M,	Eur J Hum Genet	2020
25	A	Education	32040237	Unmet information needs of men with breast cancer and health professional	Bootsma TI, Duij	Psychooncology	2020
26	A	Education	32366968	Phenotate: crowdsourcing phenotype annotations as exercises in undergrad	Chang WH, Mash	Genet Med	2020
27	A	Education	31896776	Development and mixed-methods evaluation of an online animation for you	Lewis C, Sanders	Eur J Hum Genet	2020
28	A	Education	32535291	Understanding hypermobile Ehlers-Danlos syndrome and Hypermobility Spe	Pezaro DS, Pearc	Midwifery	2020
29	A	Education	32342777	Australian Aboriginal and Torres Strait Islander Collections of Genetic Heritag	Prictor M, Huebn	J Law Med Ethics	2020
30	A	Education	34202935	Framing Effects on Decision-Making for Diagnostic Genetic Testing: Results fr	Dwyer AA, Shen	Genes (Basel)	2021
31	A	Education	33538235	AMCP Partnership Forum: Preparing for and managing rare diseases	Flavin B, Richard	J Manag Care Spe	2021

32	A	Education	34863273	Development and evaluation of a virtual patient-centered outcomes researc	Godfrey EM, Tha	Res Involv Engage	2021
33	A	Education	33735109	The progeria research foundation 10(th) international scientific workshop; re	Gordon LB, Tumi	Aging (Albany NY	2021
34	A	Education	34749427	Decision coaching for people making healthcare decisions	Jull J, Köpke S, S	Cochrane Databa	2021
35	A	Education	34296056	Illustrated State-of-the-Art Capsules of the ISTH 2020 Congress	Krishnaswamy S,	Res Pract Thromb	2021
36	A	Education	33722276	Involving people affected by a rare condition in shaping future genomic rese	Nunn JS, Gwynn	Res Involv Engage	2021
37	A	Education	30448267	A behavior-theoretic evaluation of values clarification on parental beliefs and	Paquin RS, Peina	Soc Sci Med	2021
38	A	Education	33519696	The European Reference Network for Rare Neurological Diseases	Reinhard C, Bach	Front Neurol	2021
39	A	Education	34334415	How Patient Organizations Can Drive FAIR Data Efforts to Facilitate Research	van Lin N, Paliou	J Neuromuscul Di	2021
40	A	Education	35236389	Establishing and boosting communication in the European Reference Networ	Brunelle Praschb	Orphanet J Rare	2022
41	A	Education	36420016	Genome access and other web-based IT solutions: Genetic counseling in the	Cazzaniga A, Pleb	Front Public Heal	2022
42	A	Education	36125045	Promoting validation and cross-phylogenetic integration in model organism r	Cheng KC, Burdin	Dis Model Mech	2022
43	A	Education	35417004	Report of the First International Symposium on NUT Carcinoma	French CA, Chen	Clin Cancer Res	2022
44	A	Education	35615418	ERS International Congress 2021: highlights from the Interstitial Lung Disease	Guler SA, Cuevas	ERJ Open Res	2022
45	A	Education	37180411	Building cross-border collaborations to increase diversity and accelerate rare	Khera HK, Venug	Ther Adv Rare Dis	2022
46	A	Education	36288836	Acceptability and feasibility of an online information linker service for caregi	Robertson EG, K	BMJ Open	2022
47	A	Education	35477513	YouTube-videos for patient education in lymphangioleiomyomatosis?	Wilkens FM, Gan	Respir Res	2022
48	A	Education		The Value of Online CME in the Multidisciplinary Care of Patients with NTRK	Worst, M.A.	International Jou	2022
49	A	Education	36755105	The European Society of Human Genetics-Young committee- activities and a	Avram E, Ding C,	Eur J Hum Genet	2023
50	A	Education	37518079	Development of a person-centred digital platform for the long-term support	Best, S; Al Mahm	BMJ OPEN	2023
51	A	Education	37294334	Improving care pathways for people living with rare bone diseases (RBDs): ou	Chandran M	Osteoporos Int	2023
52	A	Education	37480080	Digital health and Clinical Patient Management System (CPMS) platform utili	Fortunato, F	Orphanet J Rare	2023
53	A	Education	37053103	Skeletal Dysplasia Families: A Stepwise Approach to Diagnosis	Handa A, Grigeli	Radiographics	2023
54	A	Education	37450855	An overview of the outreach of the 2019-2021 Endo-ERN knowledge generat	Iotova V	Endocr Connect	2023
55	A	Education	36694456	Successfully Navigating Food and Drug Administration Orphan Drug and Rare	Lomash RM, Shc	Hum Gene Ther	2023
56	A	Education	36855907	Learning to make a difference for chILD: Value creation through network coll	McKnight L, Schu	Pediatr Pulmonol	2023
57	A	Education	36617168	Developing a disease-specific annotation protocol for VHL gene curation usin	Salehipour D, Fa	Database (Oxford	2023
58	A	Education	36947286	So You Want to Build Your Disease's First Online Patient Registry: An Educati	Wicks P, Wahlstr	Patient	2023
59	B	bioinformatics tool/resource	10613854	The genexpress IMAGE knowledge base of the human muscle transcriptome:	Piétu G, Eveno E	Genome Res	1999
60	B	bioinformatics tool/resource	12651711	Triplet repeats in human genome: distribution and their association with gen	Subramanian S,	Bioinformatics	2003
61	B	bioinformatics tool/resource	15978138	Distribution of immunodeficiency fact files with XML--from Web to WAP	Väliaho J, Riikon	BMC Med Inform	2005
62	B	bioinformatics tool/resource	17980034	Cubic exact solutions for the estimation of pairwise haplotype frequencies: i	Gaunt, TR; Rodri	BMC BIOINFORM	2007
63	B	bioinformatics tool/resource	18160232	snoTARGET shows that human orphan snoRNA targets locate close to alterna	Bazeley PS, Shep	Gene	2008

64	B	bioinformatics tool/resource	20046828	Disease gene characterization through large-scale co-expression analysis	Day A, Dong J, Fu	PLoS One	2009
65	B	bioinformatics tool/resource	19557189	Identifying relationships among genomic disease regions: predicting genes at	Raychaudhuri S,	PLoS Genet	2009
66	B	bioinformatics tool/resource	20377445	Haplotype Inference in Complex Pedigrees	Kirkpatrick, B; Ha	JOURNAL OF COM	2010
67	B	bioinformatics tool/resource	22467916	Large-scale analysis of conserved rare codon clusters suggests an involvemen	Chartier M, Gaud	Bioinformatics	2012
68	B	bioinformatics tool/resource	23095660	EVA: Exome Variation Analyzer, an efficient and versatile tool for filtering str	Coutant S, Cabot	BMC Bioinformat	2012
69	B	bioinformatics tool/resource	22848493	qPMS7: a fast algorithm for finding (ℓ, d)-motifs in DNA and protein sequenc	Dinh H, Rajaseka	PLoS One	2012
70	B	bioinformatics tool/resource	23736532	Identification of deleterious synonymous variants in human genomes	Buske OJ, Manic	Bioinformatics	2013
71	B	bioinformatics tool/resource	23883437	Fabry_CEP: a tool to identify Fabry mutations responsive to pharmacological	Cammisa M, Cor	Orphanet J Rare	2013
72	B	bioinformatics tool/resource	23819870	Identifying Mendelian disease genes with the variant effect scoring tool	Carter H, Douvill	BMC Genomics	2013
73	B	bioinformatics tool/resource	23740754	Encore: Genetic Association Interaction Network centrality pipeline and appl	Davis NA, Lareau	Genet Epidemiol	2013
74	B	bioinformatics tool/resource	23539306	FishingCNV: a graphical software package for detecting rare copy number va	Shi Y, Majewski J	Bioinformatics	2013
75	B	bioinformatics tool/resource	24803668	A web-based interactive framework to assist in the prioritization of disease c	Alemán A, Garcia	Nucleic Acids Res	2014
76	B	bioinformatics tool/resource	25075118	FARVAT: a family-based rare variant association test	Choi S, Lee S, Cic	Bioinformatics	2014
77	B	bioinformatics tool/resource	24733292	Association analysis using next-generation sequence data from publicly avail	Derkach A, Chian	Bioinformatics	2014
78	B	bioinformatics tool/resource	24870143	Candidate Gene Discovery and Prioritization in Rare Diseases	Jegga, AG	CLINICAL BIOINFO	2014
79	B	bioinformatics tool/resource	24217912	The Human Phenotype Ontology project: linking molecular biology and disea	Köhler S, Doelke	Nucleic Acids Res	2014
80	B	bioinformatics tool/resource	24478219	Prioritizing disease-linked variants, genes, and pathways with an interactive	Lee IH, Lee K, Hs	Hum Mutat	2014
81	B	bioinformatics tool/resource	24960519	Design and anticipated outcomes of the eMERGE-PGx project: a multicenter	Rasmussen-Torv	Clin Pharmacol T	2014
82	B	bioinformatics tool/resource		Power analysis and sample size estimation for sequence-based association st	Wang, Gao T.	Bioinformatics	2014
83	B	bioinformatics tool/resource	24916671	EFIN: predicting the functional impact of nonsynonymous single nucleotide p	Zeng S, Yang J, C	BMC Genomics	2014
84	B	bioinformatics tool/resource	25643705	BRCA1 Circos: a visualisation resource for functional analysis of missense var	Jhuraney A, Velk	J Med Genet	2015
85	B	bioinformatics tool/resource	25596308	mirTrios: an integrated pipeline for detection of de novo and rare inherited	Li J, Jiang Y, Wan	J Med Genet	2015
86	B	bioinformatics tool/resource	26231429	PBAP: a pipeline for file processing and quality control of pedigree data with	Nato AQ Jr, Chap	Bioinformatics	2015
87	B	bioinformatics tool/resource	25655794	Risk Prediction for Progression of Macular Degeneration: 10 Common and Ra	Seddon JM, Silve	Invest Ophthalmo	2015
88	B	bioinformatics tool/resource		Modeling High Dimensional Frequency Spectral Data Based on Virtual Sampl	Tang, J; Jia, MY;	2015 IEEE INTERN	2015
89	B	bioinformatics tool/resource	26394715	SEQMINER: An R-Package to Facilitate the Functional Interpretation of Seque	Zhan, XW; Liu, D	GENETIC EPIDEM	2015
90	B	bioinformatics tool/resource	27272119	FamPipe: An Automatic Analysis Pipeline for Analyzing Sequencing Data in Fa	Chung RH, Tsai W	PLoS Comput Bio	2016
91	B	bioinformatics tool/resource	26838676	A visual and curatorial approach to clinical variant prioritization and disease	James RA, Camp	Genome Med	2016
92	B	bioinformatics tool/resource	27508260	Data and programs in support of network analysis of genes and their associa	Kontou, PI; Pavlo	DATA IN BRIEF	2016
93	B	bioinformatics tool/resource	27587678	Pathway-based approach using hierarchical components of collapsed rare va	Lee S, Choi S, Kim	Bioinformatics	2016
94	B	bioinformatics tool/resource	26446135	HEALER: homomorphic computation of ExAct Logistic rEgRession for secure r	Wang S, Zhang Y	Bioinformatics	2016
95	B	bioinformatics tool/resource	27153000	RVTESTS: an efficient and comprehensive tool for rare variant association an	Zhan X, Hu Y, Li B	Bioinformatics	2016

96	B	bioinformatics tool/resource	28822752	Comprehensive Analysis of Tissue-wide Gene Expression and Phenotype Dat	Feiglin A, Allen B	Cell Syst	2017
97	B	bioinformatics tool/resource	28520890	VCF.Filter: interactive prioritization of disease-linked genetic variants from se	Müller H, Jimene	Nucleic Acids Res	2017
98	B	bioinformatics tool/resource		Rare Disease Discovery: An Optimized Disease Ranking System	Pinol, M; Alves,	IEEE TRANSACTIO	2017
99	B	bioinformatics tool/resource	28821228	SG-ADVISER mtDNA: a web server for mitochondrial DNA annotation with da	Rueda, M; Torka	BMC BIOINFORM	2017
100	B	bioinformatics tool/resource	28035032	Using genotype array data to compare multi- and single-sample variant calls	Shringarpure SS,	Bioinformatics	2017
101	B	bioinformatics tool/resource	28196472	PreMeta: a tool to facilitate meta-analysis of rare-variant associations	Tang ZZ, Bunn P,	BMC Genomics	2017
102	B	bioinformatics tool/resource	29309409	Weighted functional linear regression models for gene-based association ana	Belonogova, NM	PLOS ONE	2018
103	B	bioinformatics tool/resource	30619023	A Meta-Analysis of α-Synuclein Multiplication in Familial Parkinsonism	Book A, Guella I,	Front Neurol	2018
104	B	bioinformatics tool/resource	29459775	Formalising recall by genotype as an efficient approach to detailed phenotyp	Corbin LJ, Tan VY	Nat Commun	2018
105	B	bioinformatics tool/resource	30566525	Mucopolysaccharidosis type II detection by Naïve Bayes Classifier: An examp	Ehsani-Moghadd	PLoS One	2018
106	B	bioinformatics tool/resource	30564269	RDAD: A Machine Learning System to Support Phenotype-Based Rare Diseas	Jia J, Wang R, An	Front Genet	2018
107	B	bioinformatics tool/resource	29898714	Calculating the statistical significance of rare variants causal for Mendelian a	Rao AR, Nelson S	BMC Med Genom	2018
108	B	bioinformatics tool/resource		Data-driven Approaches to Understand Development, Diseases and Identify	Wang, Yunguan		2018
109	B	bioinformatics tool/resource	31806013	Single-nucleus chromatin accessibility reveals intratumoral epigenetic hetero	Al-Ali R, Bauer K,	Acta Neuropatho	2019
110	B	bioinformatics tool/resource	31026367	eDiVA-Classification and prioritization of pathogenic variants for clinical diag	Bosio M, Drechs	Hum Mutat	2019
111	B	bioinformatics tool/resource	30854222	Differential metabolic activity and discovery of therapeutic targets using sum	Çubuk C, Hidalgo	NPJ Syst Biol App	2019
112	B	bioinformatics tool/resource	29939197	Disease classification: from phenotypic similarity to integrative genomics and	Dozmorov, MG	BRIEFINGS IN BIO	2019
113	B	bioinformatics tool/resource		Anatomy of missense variants in health and disease : towards better impact	Laddach, Anna Christine		2019
114	B	bioinformatics tool/resource	30535356	Development and user evaluation of a rare disease gene prioritization workfl	Lee JJY, van Karn	J Am Med Inform	2019
115	B	bioinformatics tool/resource	30591557	Blacklisting variants common in private cohorts but not in public databases o	Maffucci P, Bigio	Proc Natl Acad Sc	2019
116	B	bioinformatics tool/resource	30169785	novoCaller: a Bayesian network approach for de novo variant calling from pe	Mohanty AK, Vu	Bioinformatics	2019
117	B	bioinformatics tool/resource	32760576	Development of an informatics system for accelerating biomedical research	Navale V, Ji M, V	F1000Res	2019
118	B	bioinformatics tool/resource	30407490	OPA2Vec: combining formal and informal content of biomedical ontologies t	Smaili FZ, Gao X,	Bioinformatics	2019
119	B	bioinformatics tool/resource	32434849	AMELIE speeds Mendelian diagnosis by matching patient phenotype and gen	Birgmeier J, Hae	Sci Transl Med	2020
120	B	bioinformatics tool/resource	32364235	EXpectation Propagation LOgistic REgRession on permissioned blockCHAIN (E	Kuo TT, Gabriel R	J Am Med Inform	2020
121	B	bioinformatics tool/resource	31943009	Privacy-preserving model learning on a blockchain network-of-networks	Kuo, TT; Kim, J; G	JOURNAL OF THE	2020
122	B	bioinformatics tool/resource	31691385	The Clinical Genome and Ancestry Report: An interactive web application for	Lee IH, Negron J	Hum Mutat	2020
123	B	bioinformatics tool/resource	32686118	Synthesis of diagnostic quality cancer pathology images by generative advers	Levine AB, Peng	J Pathol	2020
124	B	bioinformatics tool/resource	31368479	Bayesian estimation of genetic regulatory effects in high-throughput reporte	Majoros WH, Kim	Bioinformatics	2020
125	B	bioinformatics tool/resource	32228266	Integrated Informatics Analysis of Cancer-Related Variants	Pagel KA, Kim R,	JCO Clin Cancer In	2020
126	B	bioinformatics tool/resource	33090109	Efficiency of Computer-Aided Facial Phenotyping (DeepGestalt) in Individuals	Pantel JT, Hajjir	J Med Internet Re	2020
127	B	bioinformatics tool/resource	32283553	Structured reviews for data and knowledge-driven research	Queralt-Rosinac	Database (Oxford	2020

128	B	bioinformatics tool/resource	31866045	UK Biobank Whole-Exome Sequence Binary Phenome Analysis with Robust R	Zhao Z, Bi W, Zh	Am J Hum Genet	2020
129	B	bioinformatics tool/resource	34370400	JWES: a new pipeline for whole genome/exome sequence data processing, m	Ahmed Z, Renart	FEBS Open Bio	2021
130	B	bioinformatics tool/resource	33335023	CanRisk Tool-A Web Interface for the Prediction of Breast and Ovarian Cance	Carver T, Hartley	Cancer Epidemiol	2021
131	B	bioinformatics tool/resource	34843456	Protein domain-based prediction of drug/compound-target interactions and	Doğan T, Akhan	PLoS Comput Bio	2021
132	B	bioinformatics tool/resource	33331865	A Bayesian linear mixed model for prediction of complex traits	Hai Y, Wen Y.	Bioinformatics	2021
133	B	bioinformatics tool/resource	34346903	Construction of Genealogical Knowledge Graphs From Obituaries: Multitask	He K, Yao L, Zhan	J Med Internet Re	2021
134	B	bioinformatics tool/resource	33264411	The Human Phenotype Ontology in 2021	Köhler S, Gargan	Nucleic Acids Res	2021
135	B	bioinformatics tool/resource	33981200	Gene4PD: A Comprehensive Genetic Database of Parkinson's Disease	Li B, Zhao G, Zho	Front Neurosci	2021
136	B	bioinformatics tool/resource	34348744	10 years of CEMARA database in the AnDDI-Rares network: a unique resourc	Messiaen C, Raci	Orphanet J Rare	2021
137	B	bioinformatics tool/resource	34529933	Identifying digenic disease genes via machine learning in the Undiagnosed Di	Mukherjee, S; Co	AMERICAN JOUR	2021
138	B	bioinformatics tool/resource	33757430	ATAV: a comprehensive platform for population-scale genomic analyses	Ren Z, Povysil G,	BMC Bioinformat	2021
139	B	bioinformatics tool/resource	33400510	PIIMS Server: A Web Server for Mutation Hotspot Scanning at the Protein-Pr	Wu FX, Yang JF,	J Chem Inf Model	2021
140	B	bioinformatics tool/resource	35674381	WAT3R: recovery of T-cell receptor variable regions from 3' single-cell RNA-s	Ainciburu M, Mo	Bioinformatics	2022
141	B	bioinformatics tool/resource	36083022	The phers R package: using phenotype risk scores based on electronic health	Aref L, Bastarach	Bioinformatics	2022
142	B	bioinformatics tool/resource	36300680	VariantAlert: A web-based tool to notify updates in genetic variant annotatio	Atzeni R, Massid	Hum Mutat	2022
143	B	bioinformatics tool/resource	35389435	Exploiting deep transfer learning for the prediction of functional noncoding v	Chen L, Wang Y.	Bioinformatics	2022
144	B	bioinformatics tool/resource	35063063	PhenoApt leverages clinical expertise to prioritize candidate genes via machi	Chen Z, Zheng Y,	Am J Hum Genet	2022
145	B	bioinformatics tool/resource	36376793	Combining genetic constraint with predictions of alternative splicing to priori	Cormier, MJ; Ped	BMC BIOINFORM	2022
146	B	bioinformatics tool/resource	35441669	STAAR workflow: a cloud-based workflow for scalable and reproducible rare	Gaynor SM, Wes	Bioinformatics	2022
147	B	bioinformatics tool/resource	34859531	Variant interpretation using population databases: Lessons from gnomAD	Gudmundsson S,	Hum Mutat	2022
148	B	bioinformatics tool/resource	35970914	KBG syndrome: videoconferencing and use of artificial intelligence driven fac	Guo L, Park J, Yi	Eur J Hum Genet	2022
149	B	bioinformatics tool/resource	35179571	ACTIVA: realistic single-cell RNA-seq generation with automatic cell-type iden	Heydari AA, Dav	Bioinformatics	2022
150	B	bioinformatics tool/resource	35504290	TOP-LD: A tool to explore linkage disequilibrium with TOPMed whole-genom	Huang L, Rosen J	Am J Hum Genet	2022
151	B	bioinformatics tool/resource	36118553	HGDiscovery: An online tool providing functional and phenotypic informatio	Karmakar M, Cic	Curr Res Struct Bi	2022
152	B	bioinformatics tool/resource	35197310	A framework to score the effects of structural variants in health and disease	Kleinert P, Kirche	Genome Res	2022
153	B	bioinformatics tool/resource	36063052	ABEILLE: a novel method for ABerrant Expression Identification empLoying m	Labory J, Le Bide	Bioinformatics	2022
154	B	bioinformatics tool/resource	35209950	DrABC: deep learning accurately predicts germline pathogenic mutation stat	Liu J, Zhao H, Zhe	Genome Med	2022
155	B	bioinformatics tool/resource	35266241	seqr: A web-based analysis and collaboration tool for rare disease genomics	Pais LS, Snow H,	Hum Mutat	2022
156	B	bioinformatics tool/resource	35316210	Whole-transcriptome sequencing-based concomitant detection of viral and h	Saeidian AH, You	JCI Insight	2022
157	B	bioinformatics tool/resource	37083939	The Regulatory Mendelian Mutation score for GRCh38	Schubach M, Naz	Gigascience	2022
158	B	bioinformatics tool/resource	35953082	Interpretable surface-based detection of focal cortical dysplasias: a Multi-cen	Spitzer H, Ripart	Brain	2022
159	B	bioinformatics tool/resource	35963939	Development of an extensive workflow for comprehensive clinical pharmaco	Tafazoli A, van d	Pharmacogenomi	2022

160	B	bioinformatics tool/resource	36477604	DrugRepo: a novel approach to repurposing drugs based on chemical and ge	Wang Y, Aldahdo	Sci Rep	2022
161	B	bioinformatics tool/resource	36063453	TVAR: Assessing Tissue-specific Functional Effects of Non-coding Variants wit	Yang H, Chen R,	Bioinformatics	2022
162	B	bioinformatics tool/resource	37599175	A life-threatening bleeding prediction model for immune thrombocytopenia	An ZY	Sci Bull (Beijing)	2023
163	B	bioinformatics tool/resource	37158367	Ravages: An R package for the simulation and analysis of rare variants in mul	Bocher O, Maren	Genet Epidemiol	2023
164	B	bioinformatics tool/resource	37389415	KG-Hub-building and exchanging biological knowledge graphs	Caufield JH	Bioinformatics	2023
165	B	bioinformatics tool/resource	36750605	Identifying congenital generalized lipodystrophy using deep learning-DEEPLIP	da Cunha Olegar	Sci Rep	2023
166	B	bioinformatics tool/resource	36681873	A Solve-RD ClinVar-based reanalysis of 1522 index cases from ERN-ITHACA re	Denomme-Picho	GENETICS IN MED	2023
167	B	bioinformatics tool/resource	36322820	PEMT: a patent enrichment tool for drug discovery	Gadiya Y, Zaliani	Bioinformatics	2023
168	B	bioinformatics tool/resource	36752514	ARAX: a graph-based modular reasoning tool for translational biomedicine	Glen, AK; Ma, CY	BIOINFORMATICS	2023
169	B	bioinformatics tool/resource	37187615	EdeepSADPr: an extensive deep-learning architecture for prediction of the in	Jiang H, Shang S,	Front Cell Dev Bio	2023
170	B	bioinformatics tool/resource	36855134	Precision information extraction for rare disease epidemiology at scale	Kariampuzha WZ	J Transl Med	2023
171	B	bioinformatics tool/resource	37312221	Var|Decrypt: a novel and user-friendly tool to explore and prioritize variants	Salma, M	Epigenetics Chro	2023
172	B	bioinformatics tool/resource	37232043	Predicting molecular mechanisms of hereditary diseases by using their tissue	Simonovsky, E	Mol Syst Biol	2023
173	B	bioinformatics tool/resource	37101200	Extending inherited metabolic disorder diagnostics with biomarker interactio	Slenter DN, Hem	Orphanet J Rare	2023
174	B	bioinformatics tool/resource	37637211	Cis-Cardio: A comprehensive analysis platform for cardiovascular-relavant cis	Song, C	Mol Ther Nucleic	2023
175	B	bioinformatics tool/resource	37257586	ChatGPT: a pioneering approach to complex prenatal differential diagnosis	Suhag A, Kidd J,	Am J Obstet Gyne	2023
176	B	bioinformatics tool/resource	37296794	Design and Development of IoT and Deep Ensemble Learning Based Model fo	Venkatachala Ap	Diagnostics (Base	2023
177	B	bioinformatics tool/resource	37605652	Identifying oxidative stress-related biomarkers in idiopathic pulmonary fibro	Yang, F	EPMA J	2023
178	B	clinical intervention		Haptics as an Aid to Copying for People with Williams Syndrome	Lee, J; Okamura,	WORLD HAPTICS	2009
179	B	clinical intervention		An investigation into semantic and phonological processing in individuals wit	Cheryl S. Lee and	Journal of Speech	2014
180	B	clinical intervention	25606367	Parent-delivered touchscreen intervention for children with fragile X syndro	Díez-Juan M, Sch	Intractable Rare	2014
181	B	clinical intervention		ARE VIRTUAL REALITY APPLICATIONS EFFECTIVE IN RUSSELL-SILVER SYNDRO	DENGİZ, AZIZ	Journal of Exercis	2018
182	B	clinical intervention	30355560	Mind-Body Treatment for International English-Speaking Adults With Neurof	Vranceanu AM, Z	JMIR Res Protoc	2018
183	B	clinical intervention		Cognitive Rehabilitation Using Virtual Reality for Children with Rare Diseases	Jacho-Guanoluis	PROCEEDINGS OF	2019
184	B	clinical intervention	32917306	Congenital amusia and academic performance among Colombian university s	Pradilla, I; Tierra	TRENDS IN NEUR	2020
185	B	clinical intervention	32634485	Virtual mind-body treatment for adolescents with neurofibromatosis: Study	Reichman M, Rik	Contemp Clin Tria	2020
186	B	clinical intervention	32354651	Guidance for the care of neuromuscular patients during the COVID-19 pande	Solé G, Salort-Ca	Rev Neurol (Paris	2020
187	B	clinical intervention	33197677	Effects of a mind-body program on symptoms of depression and perceived s	Carter S, Greenb	Complement The	2021
188	B	clinical intervention	33191871	Resilient youth with neurofibromatosis: Less perceived stress and greater life	Lester E, Vrance	J Psychosoc Onco	2021
189	B	clinical intervention	32421171	Mind-Body Therapy via Videoconferencing in Patients With Neurofibromatos	Lester EG, Gates	Ann Behav Med	2021
190	B	clinical intervention	34110294	Adaptation of a Live Video Mind-Body Program to a Web-Based Platform for	Lester EG, Hopki	JMIR Res Protoc	2021
191	B	clinical intervention	37454469	Cortico-Cerebellar neurodynamics during social interaction in Autism Spectru	Gaudfernau F	Neuroimage Clin	2023

192	B	crowdsourcing	26288840	Crowdsourcing the General Public for Large Scale Molecular Pathology Studi	Candido Dos Rei	EBioMedicine	2015
193	B	crowdsourcing	27767221	From Gutenberg to Open Science: An Unfulfilled Odyssey	Triggle CR, Triggl	Drug Dev Res	2017
194	B	crowdsourcing	32990755	CSVS, a crowdsourcing database of the Spanish population genetic variability	Peña-Chilet M, R	Nucleic Acids Res	2021
195	B	database/registry/network	10640220	Familial myeloproliferative disease	Gilbert HS.	Baillieres Clin Hae	1998
196	B	database/registry/network		Progeroid Syndromes.	Homack, Susan	Handbook of neu	2005
197	B	database/registry/network	24980845	The innovative use of a large-scale industry biomedical consortium to resear	Holden AL.	Drug Discov Toda	2007
198	B	database/registry/network	20428338	Cildb: a knowledgebase for centrosomes and cilia	Arnaiz O, Malino	Database (Oxford	2009
199	B	database/registry/network	18954903	Juvenile myelomonocytic leukemia: a report from the 2nd International JMM	Chan RJ, Cooper	Leuk Res	2009
200	B	database/registry/network	20501980	The European disorder of sex development registry: a virtual research enviro	Ahmed SF, Rodie	Sex Dev	2010
201	B	database/registry/network	20235890	The International LAM Registry: a component of an innovative web-based cli	Nurok M, Eslick I	Lymphat Res Biol	2010
202	B	database/registry/network	21463291	The impact of parent advocacy groups, the Internet, and social networking o	Black AP, Baker	Epilepsia	2011
203	B	database/registry/network	21174622	Population-specific documentation of pharmacogenomic markers and their a	Georgitsi M, Vie	Pharmacogenomi	2011
204	B	database/registry/network	21880229	Creation of the Web-based University of Chicago Monogenic Diabetes Regist	Greeley SA, Nayl	J Diabetes Sci Tec	2011
205	B	database/registry/network	22099354	Knowledge and therapeutic gaps: a public health problem in the rare coagula	Shapiro AD, Souc	Am J Prev Med	2011
206	B	database/registry/network	22753342	A modular approach to disease registry design: successful adoption of an int	Bellgard MI, Mac	Hum Mutat	2012
207	B	database/registry/network	22241657	Interpretation of genomic copy number variants using DECIPHER	Corpas M, Bragin	Curr Protoc Hum	2012
208	B	database/registry/network	22364447	Advances in primary immunodeficiency diseases in Latin America: epidemiol	Errante, PR; Fran	YEAR IN HUMAN	2012
209	B	database/registry/network	22704798	A national internet-linked based database for pediatric interstitial lung diseas	Nathan N, Taam	Orphanet J Rare	2012
210	B	database/registry/network	23324478	Disease and patient characteristics in NP-C patients: findings from an interna	Patterson MC, M	Orphanet J Rare	2013
211	B	database/registry/network	24503589	The current situation and needs of rare disease registries in Europe	Taruscio D, Gain	Public Health Gen	2013
212	B	database/registry/network	23920006	Portal for Families Overcoming Neurodevelopmental Disorders (PFOND): Imp	Ye XC, Ng I, Seid-	JMIR Res Protoc	2013
213	B	database/registry/network	24569870	Rare disease registries: an initiative to establish vasculitis registry in Poland	Padjas A, Sznajd	Pol Arch Med We	2014
214	B	database/registry/network	25160272	Vision and challenges of a cartographic representation of expert medical cen	Storf H, Hartz T,	Stud Health Tech	2014
215	B	database/registry/network	26958175	LORD: a phenotype-genotype semantically integrated biomedical data tool t	Choquet R, Maar	AMIA Annu Symp	2015
216	B	database/registry/network	25886171	Rationale, design and objectives of ARegPKD, a European ARPKD registry stu	Ebner K, Feldkoe	BMC Nephrol	2015
217	B	database/registry/network	25038238	Phenotypic and genotypic characteristics of cryopyrin-associated periodic sy	Levy R, Gérard L,	Ann Rheum Dis	2015
218	B	database/registry/network		The Comparison of the Efficacy of Once and Twice Daily Colchicine Dosage in Pediatric Patients With FMF	NCT02602028	https://clinicaltrials.gov/show/NCT02602028	2015
219	B	database/registry/network		The European Thoracic Oncology Platform (ETOP)	Peters S	Annals of Oncolo	2015
220	B	database/registry/network	26596705	Undiagnosed Diseases Network International (UDNI): White paper for global	Taruscio D, Groft	Mol Genet Metab	2015
221	B	database/registry/network	26738761	Novel features of 3q29 deletion syndrome: Results from the 3q29 registry	Glassford MR, Ro	Am J Med Genet	2016
222	B	database/registry/network	27021300	Genotyping of 22 blood group antigen polymorphisms and establishing a nat	Hong YJ, Chung Y	Ann Hematol	2016
223	B	database/registry/network	27630202	Establishment of Kawasaki disease database based on metadata standard	Park YR, Kim JJ, Y	Database (Oxford	2016

224	B	database/registry/network	26659107	An international registry for primary ciliary dyskinesia	Werner C, Lablan	Eur Respir J	2016
225	B	database/registry/network	28344196	Design of the Familial Hypercholesterolaemia Australasia Network Registry:	Bellgard MI, Wal	J Atheroscler Thr	2017
226	B	database/registry/network	28437535	Clinical Aspects of Glucose Transporter Type 1 Deficiency: Information From	Hao J, Kelly DI, S	JAMA Neurol	2017
227	B	database/registry/network	29047407	A web-based collection of genotype-phenotype associations in hereditary re	Papa R, Doglio M	Orphanet J Rare	2017
228	B	database/registry/network	28637735	Development of the Tailored Rett Intervention and Assessment Longitudinal	Santosh P, Lieve	BMJ Open	2017
229	B	database/registry/network	27924022	Expanded national database collection and data coverage in the FINDbase w	Viennas E, Komia	Nucleic Acids Res	2017
230	B	database/registry/network	28502612	MARRVEL: Integration of Human and Model Organism Genetic Resources to	Wang J, Al-Oura	Am J Hum Genet	2017
231	B	database/registry/network	30033519	Developing a database for Rett syndrome research performed in the Europe	Akinsolu FT, Balc	Child Care Health	2018
232	B	database/registry/network	30245029	Genomic Landscape and Mutational Signatures of Deafness-Associated Gene	Azaiez H, Booth	Am J Hum Genet	2018
233	B	database/registry/network	28190666	Design of a framework for the deployment of collaborative independent rare	Bellgard MI, Nap	Blood Cells Mol D	2018
234	B	database/registry/network	30563557	Evaluation of DSD training schools organized by cost action BM1303 "DSDnet	Bertalan R, Lucas	Orphanet J Rare	2018
235	B	database/registry/network	29396563	The RD-Connect Registry & Biobank Finder: a tool for sharing aggregated dat	Gainotti S, Torre	Eur J Hum Genet	2018
236	B	database/registry/network	29305133	Phenotypic stratification and genotype-phenotype correlation in a heterogen	Pogoryelova O, C	Neuromuscul Dis	2018
237	B	database/registry/network	29633452	Research protocol: The initiation, design and establishment of the Global An	Tones M, Cross	J Intellect Disabil	2018
238	B	database/registry/network	30134969	Characteristics of undiagnosed diseases network applicants: implications for	Walley NM, Pen	BMC Health Serv	2018
239	B	database/registry/network	30185236	Eight years after an international workshop on myotonic dystrophy patient r	Wood L, Bassez	Orphanet J Rare	2018
240	B	database/registry/network	29685812	The CF Canada-Sick Kids Program in individual CF therapy: A resource for the	Eckford, PDW; M	JOURNAL OF CYS	2019
241	B	database/registry/network	31239751	Tracing the effect of the melanocortin-4 receptor pathway in obesity: study	Eneli I, Xu J, Web	Appl Clin Genet	2019
242	B	database/registry/network	31343797	Iranome: A catalog of genomic variations in the Iranian population	Fattahi Z, Behesh	Hum Mutat	2019
243	B	database/registry/network	30773687	Phenotype, treatment practice and outcome in the cobalamin-dependent re	Huemer M, Diod	J Inherit Metab D	2019
244	B	database/registry/network	31234869	De-duplicating patient records from three independent data sources reveals	König K, Pechma	Orphanet J Rare	2019
245	B	database/registry/network	31475990	Navigating MARRVEL, a Web-Based Tool that Integrates Human Genomics an	Wang J, Liu Z, Be	J Vis Exp	2019
246	B	database/registry/network	31551357	Fostering open collaboration in drug development for paediatric brain tumo	Wong JF, Brown	Biochem Soc Tran	2019
247	B	database/registry/network	32161151	Use of a rare disease registry for establishing phenotypic classification of pre	Germain DP, Oliv	J Med Genet	2020
248	B	database/registry/network	32156722	Li-Fraumeni Exploration Consortium Data Coordinating Center: Building an In	Mai PL, Sand SR,	Cancer Epidemiol	2020
249	B	database/registry/network	32783387	Implementation of a registry and open access genetic testing program for inh	Mansfield BC, Ye	Am J Med Genet	2020
250	B	database/registry/network	33109251	Design, development and deployment of a web-based interoperable registry	Marques JP, Carv	Orphanet J Rare	2020
251	B	database/registry/network	32849507	Primary Immunodeficiencies in Russia: Data From the National Registry	Mukhina AA, Kuz	Front Immunol	2020
252	B	database/registry/network	32042194	The Angiosarcoma Project: enabling genomic and clinical discoveries in a rar	Painter CA, Jain E	Nat Med	2020
253	B	database/registry/network	32601614	The North American mitochondrial disease registry	Rosales XQ, Tho	J Transl Genet Ge	2020
254	B	database/registry/network	32293014	Prot2HG: a database of protein domains mapped to the human genome	Stanek D, Bis-Bre	Database (Oxford	2020
255	B	database/registry/network	34758253	100,000 Genomes Pilot on Rare-Disease Diagnosis in Health Care - Prelimina	100,000 Genom	N Engl J Med	2021

256	B	database/registry/network	35047757	How to define and enhance diagnostic and assistance pathways in neuromus	Astrea G, Marine	Acta Myol	2021
257	B	database/registry/network	34078418	The European Rare Kidney Disease Registry (ERKReg): objectives, design and	Bassanese G, Wl	Orphanet J Rare	2021
258	B	database/registry/network	33325392	Iranian Registry of Duchenne and Becker Muscular Dystrophies: Characteriza	Bayat F, Sarmien	J Neuromuscul Di	2021
259	B	database/registry/network	33539200	Classification and Personalized Prognostic Assessment on the Basis of Clinica	Bersanelli M, Tra	J Clin Oncol	2021
260	B	database/registry/network	34772435	An Integrated clinical pathway for diagnosis, treatment and care of rare dise	Choukair D, Hau	Orphanet J Rare	2021
261	B	database/registry/network	33406409	Spatiotemporal analysis of human intestinal development at single-cell resol	Fawkner-Corbett	Cell	2021
262	B	database/registry/network	34654619	Natural history of children and adults with phenylketonuria in the NBS-PKU C	Kenneson A, Sing	Mol Genet Metab	2021
263	B	database/registry/network	33625877	OncoTree: A Cancer Classification System for Precision Oncology	Kundra R, Zhang	JCO Clin Cancer In	2021
264	B	database/registry/network	33527330	Collaboration for rare diabetes: understanding new treatment options for W	Reschke F, Roha	Endocrine	2021
265	B	database/registry/network	34147352	MPSBase: Comprehensive repository of differentially expressed genes for m	Soares LDF, Villal	Mol Genet Metab	2021
266	B	database/registry/network	34301277	The LORIS MyeliNeuroGene rare disease database for natural history studies	Spahr A, Rosli Z,	Orphanet J Rare	2021
267	B	database/registry/network	34248822	The ARCA Registry: A Collaborative Global Platform for Advancing Trial Readi	Traschütz A, Reic	Front Neurol	2021
268	B	database/registry/network	33791951	The Global Registry for Hereditary Angioedema due to C1-Inhibitor Deficienc	Zanichelli A, Fark	Clin Rev Allergy I	2021
269	B	database/registry/network	36226147	An overlook on the current registries for rare and complex connective tissue	Bandeira M, Di C	Front Med (Lausa	2022
270	B	database/registry/network	35148959	Centers for Mendelian Genomics: A decade of facilitating gene discovery	Baxter SM, Pose	Genet Med	2022
271	B	database/registry/network	35143074	DECIPHER: Supporting the interpretation and sharing of rare disease phenoty	Foreman J, Brent	Hum Mutat	2022
272	B	database/registry/network	35143083	Advances in the development of PubCaseFinder, including the new applicatio	Fujiwara T, Shin	Hum Mutat	2022
273	B	database/registry/network	35959404	The White Matter Rounds experience: The importance of a multidisciplinary	Huang YT, Giaco	Front Neurol	2022
274	B	database/registry/network	35292119	Semantic modelling of common data elements for rare disease registries, an	Kaliyaperumal R,	J Biomed Semant	2022
275	B	database/registry/network	36209187	The Korean undiagnosed diseases program phase I: expansion of the nationw	Kim SY, Lee S, W	Orphanet J Rare	2022
276	B	database/registry/network	36028864	Challenges, facilitators and barriers to the adoption and use of a web-based	Marques JP, Vaz-	Orphanet J Rare	2022
277	B	database/registry/network	35346288	Nationwide comprehensive epidemiological study of rare diseases in Japan u	Ninomiya K, Oku	Orphanet J Rare	2022
278	B	database/registry/network	35854364	Standardised data on initiatives-STARDIT: Beta version	Nunn JS, Shafee	Res Involv Engage	2022
279	B	database/registry/network	35842788	Biallelic NLRP7 variants in patients with recurrent hydatidiform mole: A revie	Slim R, Fisher R,	Hum Mutat	2022
280	B	database/registry/network	35349419	The added value of a European Reference Network on rare and complex con	Talarico R, Aguile	Clin Exp Rheumat	2022
281	B	database/registry/network	35899212	Development and Implementation of the AIDA International Registry for Pati	Vitale A, Caggian	Front Med (Lausa	2022
282	B	database/registry/network	37308910	Expanding genotype-phenotype correlations in FOXG1 syndrome: results fro	Brimble, E; Reye	ORPHANET JOUR	2023
283	B	database/registry/network	36436153	GenIDA: an international participatory database to gain knowledge on health	Burger P, Colin F	J Neural Transm (	2023
284	B	database/registry/network	36692622	Pediatric biobanks to enhance clinical and translational research for children	Cianflone, A; Sav	EUROPEAN JOUR	2023
285	B	database/registry/network	36599466	Lymphangioleiomyomatosis (LAM) Cell Atlas	Du Y, Guo M, Wu	Thorax	2023
286	B	database/registry/network	36399497	IntroVerse: a comprehensive database of introns across human tissues	García-Ruiz S, Gu	Nucleic Acids Res	2023
287	B	database/registry/network	37414863	The Korean Genetic Diagnosis Program for Rare Disease Phase II: outcomes o	Kim, MJ	Eur J Hum Genet	2023

288	B	database/registry/network	36810103	Areas of improvement in the medical care of SMA: evidence from a nationwi	Leibrock B, Land	Orphanet J Rare	2023
289	B	database/registry/network		SympGAN: A systematic knowledge integration system for symptom-gene as	Lu, KZ; Yang, K; S	KNOWLEDGE-BAS	2023
290	B	database/registry/network	36916322	ERN ReCONNET points to consider for treating patients living with autoimmu	Talarico R, Ramir	Clin Exp Rheumat	2023
291	B	database/registry/network	37236007	Global reach of over 20 years of experience in the patient-centered Fabry Re	Wanner, C; Ortiz	MOLECULAR GEN	2023
292	B	database/registry/network	37516995	Neuromuscular disease genetics in underrepresented populations: increasing	Wilson LA	Brain	2023
293	B	Delphi consensus/guidelines	24084372	Assessment and management of nutrition and growth in Rett syndrome	Leonard H, Ravik	J Pediatr Gastroe	2013
294	B	Delphi consensus/guidelines	25123424	Delphi consensus on the current clinical and therapeutic knowledge on Ande	Concolino D, Deg	Eur J Intern Med	2014
295	B	Delphi consensus/guidelines	24884811	Multicentre consensus recommendations for skin care in inherited epidermo	El Hachem M, Za	Orphanet J Rare	2014
296	B	Delphi consensus/guidelines	26849438	Clinical Guidelines for Management of Bone Health in Rett Syndrome Based	Jefferson A, Leon	PLoS One	2016
297	B	Delphi consensus/guidelines	31059973	Recommendations for the organisation of care in paediatric radiation oncolo	Janssens GO, Tim	Eur J Cancer	2019
298	B	Delphi consensus/guidelines	32066461	Consensus protocols for the diagnosis and management of the hereditary au	Hansmann S, Lai	Pediatr Rheumat	2020
299	B	Delphi consensus/guidelines	34000449	Management of CLN1 Disease: International Clinical Consensus	Augustine, EF; A	PEDIATRIC NEUR	2021
300	B	Delphi consensus/guidelines	33949696	Toward a better definition of focal cortical dysplasia: An iterative histopathol	Blümcke I, Coras	Epilepsia	2021
301	B	Delphi consensus/guidelines	33657677	Supportive care in the acute phase of Stevens-Johnson syndrome and toxic e	Brüggen MC, Le	Br J Dermatol	2021
302	B	Delphi consensus/guidelines	33910590	Optimizing language for effective communication of gene therapy concepts	Hart DP, Branchf	Orphanet J Rare	2021
303	B	Delphi consensus/guidelines	34094869	Development of a practical dietitian road map for the nutritional manageme	Rocha JC, Bausel	Mol Genet Metab	2021
304	B	Delphi consensus/guidelines	33867362	A Canadian Adult Spinal Muscular Atrophy Outcome Measures Toolkit: Resul	Slayter J, Hodgki	J Neuromuscul Di	2021
305	B	Delphi consensus/guidelines	34362411	Expert opinion on diagnosing, treating and managing patients with cerebrote	Stelten BML, Dot	Orphanet J Rare	2021
306	B	Delphi consensus/guidelines	35272130	Managing hematological cancer patients during the COVID-19 pandemic: an	Buske C, Dreylin	ESMO Open	2022
307	B	Delphi consensus/guidelines	35507016	The Gene Curation Coalition: A global effort to harmonize gene-disease evid	DiStefano MT, G	Genet Med	2022
308	B	Delphi consensus/guidelines	35481685	Nomenclature of Genetic Movement Disorders: Recommendations of the Int	Lange LM, Gonza	Mov Disord	2022
309	B	Delphi consensus/guidelines	35497649	The international WAO/EAACI guideline for the management of hereditary a	Maurer M, Mage	World Allergy Org	2022
310	B	Delphi consensus/guidelines	35436894	Current challenges and opportunities in the care of patients with fibrodyspla	Pignolo RJ, Bedfo	Orphanet J Rare	2022
311	B	Delphi consensus/guidelines	35698202	Literature review and expert opinion on the impact of achondroplasia on me	Savarirayan R, Ba	Orphanet J Rare	2022
312	B	Delphi consensus/guidelines	35441707	Common data elements to standardize genomics studies in cerebral palsy	Wilson YA, Smith	Dev Med Child N	2022
313	B	Delphi consensus/guidelines	36981643	Enhancing Equitable Access to Rare Disease Diagnosis and Treatment around	Adachi T, El-Hatt	Int J Environ Res	2023
314	B	Delphi consensus/guidelines	37349772	Nutrition management of PKU with pegvaliase therapy: update of the web-b	Cunningham A	Orphanet J Rare	2023
315	B	Delphi consensus/guidelines	37230508	Recommendations for the development, implementation, and reporting of c	Hohenschurz-Sch	BMJ	2023
316	B	Delphi consensus/guidelines	36606566	Generalized pustular psoriasis: A global Delphi consensus on clinical course,	Puig L, Choon SE	J Eur Acad Derma	2023
317	B	Delphi consensus/guidelines	37354872	Development of Continuum of Care for McArdle disease: A practical tool for	Reason, SL; Voer	NEUROMUSCULA	2023
318	B	Delphi consensus/guidelines	36657764	Novel mixed-method, inclusive protocol involving global key stakeholders, in	Walsh C, Leavey	BMJ Open	2023
319	B	knowledge assessment	23484150	The Italian National External quality assessment program in molecular geneti	Censi F, Tosto F,	Biomed Res Int	2013

320	B	knowledge assessment	24357847	The Angelina effect: immediate reach, grasp, and impact of going public	Borzekowski DL,	Genet Med	2014
321	B	knowledge assessment	31342580	Assessment of predicted enzymatic activity of alpha-N-acetylglucosaminidas	Clark, WT; Kasak	HUMAN MUTATI	2019
322	B	knowledge assessment	34066384	Current Challenges of Cardiac Amyloidosis Awareness among Romanian Card	Adam R, Neculae	Diagnostics (Base	2021
323	B	knowledge assessment	34122502	Knowledge and Attitudes of Future Healthcare Professionals Toward Rare Di	Domaradzki J, W	Front Genet	2021
324	B	knowledge assessment	33749512	Myeloproliferative neoplasm questionnaire: assessing patient disease knowl	Pemmaraju N, W	Leuk Lymphoma	2021
325	B	knowledge assessment	36457581	Knowledge level of medical students and physicians about rare diseases in Li	Flores A, Burgos	Intractable Rare	2022
326	B	knowledge assessment		Awareness On Sotos Syndrome Among Dental Students	Harini, B	Journal of Positiv	2022
327	B	knowledge assessment	34888898	Knowledge, attitudes, and practices of healthcare professionals working in p	Yang J, Chen M,	Prenat Diagn	2022
328	B	knowledge assessment	36534338	Evaluating visual imagery for participant understanding of research concepts	Rothwell E, Rich	J Community Gen	2023
329	B	matchmaking	26251998	PhenomeCentral: a portal for phenotypic and genotypic matchmaking of pat	Buske OJ, Girdea	Hum Mutat	2015
330	B	matchmaking	26255989	The Matchmaker Exchange API: automating patient matching through the ex	Buske OJ, Schiett	Hum Mutat	2015
331	B	matchmaking	25703386	GeneYenta: a phenotype-based rare disease case matching tool based on on	Gottlieb MM, Ar	Hum Mutat	2015
332	B	matchmaking	26220891	GeneMatcher: a matching tool for connecting investigators with an interest i	Sobreira N, Schie	Hum Mutat	2015
333	B	matchmaking	28603714	Defining Disease, Diagnosis, and Translational Medicine within a Homeostati	Gall T, Valkanas	Front Med (Lausa	2017
334	B	matchmaking	29214177	Linked Registries: Connecting Rare Diseases Patient Registries through a Sem	Sernadela P, Gon	Biomed Res Int	2017
335	B	matchmaking	29044468	Matchmaker Exchange	Sobreira NLM, A	Curr Protoc Hum	2017
336	B	matchmaking	35224820	ModelMatcher: A scientist-centric online platform to facilitate collaborations	Harnish JM, Li L,	Hum Mutat	2022
337	B	matchmaking	37503210	GestaltMatcher Database - a FAIR database for medical imaging data of rare	Lesmann H	medRxiv	2023
338	B	Non-academic literature		In his skin: while doing research for a middle school assignment, John Buehle	Stephen Fraser	Current Science,	2005
339	B	Non-academic literature		New Website Offers Comprehensive Information on Hunter Syndrome		PR Newswire	2005
340	B	Non-academic literature		Select ataxia fact sheets are now available in Spanish		Generations	2009
341	B	Non-academic literature		Serono Symposia International Foundation (SSIF) Launches a New Web Site Dedicated to Med		PR Newswire	2010
342	B	Non-academic literature		New Website Dedicated to Patients with Thymic Cancer		PR Newswire	2013
343	B	Non-academic literature		New Website Dedicated to Sufferers of Melanoma		PR Newswire	2013
344	B	Non-academic literature		Researchers from University Federico II Provide Details of New Studies and Findings in the Are		Education Busine	2017
345	B	Non-academic literature		Aruvant Announces Data Presentation on Lead Program ARU-1801 at European Hematology A		PR Newswire	2021
346	B	Non-academic literature		EdOrG hosts virtual walkathon for A-T		UWIRE Text	2021
347	B	Non-academic literature		Rare Liver Disease Summit Seeks to Improve Patient Health Outcomes.		PR Newswire	2022
348	B	Non-English	10645649	[Genetic diseases: recent scientific findings and health and ethical problems]	Taruscio D, D'Ag	Ann Ist Super San	1999
349	B	Non-English	23021574	[Perception of rare diseases by the primary care physicians]	Avellaneda Fern	Semergen	2012
350	B	Non-English	25962227	[News in osteogenesis imperfecta: from research to clinical management]	Aubry-Rozier B,	Rev Med Suisse	2015
351	B	Non-English	27377324	[Spanish patients with central hypoventilation syndrome included in the Euro	García Teresa M	An Pediatr (Barc)	2017

352	B	Non-English		Collaborative e-Health Environments: The enhanced role of health agents	Jorente, MJV; Na	TRANSINFORMAC	2019
353	B	Non-English	31243508	[Model for personalized diagnostics and treatment in neurology-German Aca	Münchau A, Sch	Nervenarzt	2019
354	B	Non-English	31576998	[A qualitative approach to rare genetic diseases: an integrative review of the	Souza ÍP, Androl	Cien Saude Colet	2019
355	B	Non-English	32634889	[The first edition of oral rare diseases list]	Duan XH; Society	Zhonghua Kou Qi	2020
356	B	Non-English	36102313	[Italian Cystic Fibrosis Registry (ICFR). Report 2019-2020]	Campagna G, Am	Epidemiol Prev	2022
357	B	Non-English	35752506	[French practical guidelines for the diagnosis and management of IPF - 2021	Cottin V, Bonnia	Rev Mal Respir	2022
358	B	Non-English	35578923	[The Hokkaido Medical Personnel Training Plan Connecting Humans and Med	Saito T, Sakurai A	Gan To Kagaku Ry	2022
359	B	Non-English	37582888	[Diagnostics and management of patients with inherited retinal diseases in G	Künzel SH	Ophthalmologie	2023
360	B	resource list	17183579	The role of National Library of Medicine web sites in newborn screening edu	Fomous C, Miller	Ment Retard Dev	2006
361	B	resource list	17324666	Single-gene autosomal recessive disorders and Prader-Willi syndrome: an up	Isaacs JS, Zand D	J Am Diet Assoc	2007
362	B	resource list	18006790	New issues in genetic counseling of hereditary colon cancer	Lynch PM.	Clin Cancer Res	2007
363	B	resource list	23294802	An Update of Childhood Genetic Disorders	Prows, CA; Hopk	JOURNAL OF NUR	2013
364	B	resource list	24014152	Practical aspects of recruitment and retention in clinical trials of rare genetic	DeWard SJ, Wils	J Genet Couns	2014
365	B	resource list		Executive function and occupational therapy: Lessons drawn from the literat	Driscoll, Jude		2014
366	B	resource list		2018 NEWBORN SCREENING RESOURCES	Amelia Mulford	The Exceptional	2018
367	B	resource list		2018 GENETIC RESOURCES	Jordan Capizola	The Exceptional	2018
368	B	resource list	29129805	Rare genetic diseases: update on diagnosis, treatment and online resources	Pogue RE, Cavalc	Drug Discov Toda	2018
369	B	resource list	30393376	Foundation of the Newborn Screening Translational Research Network and it	Lloyd-Puryear M	Genet Med	2019
370	B	resource list	31501267	Genetic Counseling and Genome Sequencing in Pediatric Rare Disease	Elliott AM.	Cold Spring Harb	2020
371	B	resource list	32299466	Diagnosis support systems for rare diseases: a scoping review	Faviez C, Chen X,	Orphanet J Rare	2020
372	B	resource list		A STORY OF A RARE GENETIC MUTATION: AN EXCEPTIONAL BOY AND RESOU	Kelly Medellin	The Exceptional	2022
373	B	resource list	37653461	Initiatives to promote access to medicines after publication of the Brazilian P	Cunico, C	Orphanet J Rare	2023
374	B	resource list	37234922	Resources and tools for rare disease variant interpretation	Licata L, Via A, T	Front Mol Biosci	2023
375	B	social media/web search		The next big library legislative issue: taxpayers are storming the fee-based ba	Ray English and	American Librarie	2006
376	B	social media/web search	20937053	One wants to know what a chromosome is': the internet as a coping resourc	Gundersen T.	Sociol Health Illn	2011
377	B	social media/web search		ViroPharma Launches Hereditary Angioedema Facebook App to Encourage Education and Dia		PR Newswire	2012
378	B	social media/web search	24565858	Using the internet to seek information about genetic and rare diseases: a cas	Morgan T, Schmi	JMIR Res Protoc	2014
379	B	social media/web search	25540896	Genetic Information-Seeking Behaviors and Knowledge among Family Memb	Hamilton JG, Hut	J Genet Couns	2015
380	B	social media/web search	27929804	Familial Creutzfeldt-Jakob Disease: Case report and role of genetic counselin	Clift K, Guthrie K	Prion	2016
381	B	social media/web search	28115298	The Use of Social Media to Recruit Participants With Rare Conditions: Lynch	Burton-Chase, A	JMIR RESEARCH P	2017
382	B	social media/web search		The digital epidemiology of phenylketonuria, aka folling's disease: Retrospec	Al-Imam, Ahmed	Asian Journal of	2018
383	B	social media/web search	30425372	How Facebook and Twitter could be the next disruptive force in clinical trials	Ledford H.	Nature	2018

384	B	social media/web search	29130143	Incorporating Social Media into your Support Tool Box: Points to Consider fro	Rocha HM, Savat	J Genet Couns	2018
385	B	social media/web search	30243302	Late onset hyperornithinemia-hyperammonemia-homocitrullinuria syndrom	Silfverberg T, Sah	J Med Case Rep	2018
386	B	social media/web search	30287627	Hereditary angioedema: a mother diagnosing her child using Google as a dia	Srikantharajah T,	BMJ Case Rep	2018
387	B	social media/web search	30569339	"Before Facebook and before social media...we did not know anybody else th	Barton KS, Wing	J Community Gen	2019
388	B	social media/web search	31763986	Exploring Research Priorities of Parents Who Have Children With Down Synd	Sinclair M, McCu	J Med Internet Re	2019
389	B	social media/web search	32125658	How genomic information is accessed in clinical practice: an electronic surve	Evans WRH, Tran	J Community Gen	2020
390	B	social media/web search	32601388	Patient and family social media use surrounding a novel treatment for a rare	Iyer AA, Barzilay	Genet Med	2020
391	B	social media/web search	32865499	Assessing Public Opinion on CRISPR-Cas9: Combining Crowdsourcing and Dee	Müller M, Schne	J Med Internet Re	2020
392	B	social media/web search		Categories of Information Need Expressed by Parents of Individuals with Rar	Wang, T; Lund, B	JOURNAL OF CON	2020
393	B	social media/web search	34779778	Using Social Media as a Research Tool for a Bespoke Web-Based Platform for	Sinclair M, McCu	JMIR Pediatr Pare	2021
394	B	social media/web search	35167716	Digital haemophilia: Insights into the use of social media for haemophilia car	Chen R, Muralid	Haemophilia	2022
395	B	social media/web search	35844878	Promoting Research, Awareness, and Discussion on AI in Medicine Using #M	Nawaz FA, Barr A	Front Public Heal	2022
396	B	social media/web search	37306055	Using a health observance event to raise awareness: An assessment of World	Kleven DL	Birth Defects Res	2023
397	B	survey/interview	15176498	Results of an on-line survey of patients with hereditary angioedema	Huang SW.	Allergy Asthma P	2004
398	B	survey/interview	17003536	Understanding the information needs of general practitioners managing a ra	Zack P, Devile C,	Community Gene	2006
399	B	survey/interview	17710871	Children with 4q-syndrome: the parents' perspective	Strehle EM, Mid	Genet Couns	2007
400	B	survey/interview	20150778	Neonatal experiences of newborns with full trisomy 18	Bruns DA.	Adv Neonatal Car	2010
401	B	survey/interview		Parents' experiences of expanded newborn screening.	DeLuca, Jane M	Parents' Experien	2010
402	B	survey/interview		Prader-Willi Syndrome: Parent Perceptions of School, Professional, Social, an	Lowe-Greenlee,	ProQuest LLC	2010
403	B	survey/interview		Advocating for Inclusion of Children with Williams Syndrome	Self, Michelle A.	ProQuest LLC	2010
404	B	survey/interview	21990216	Birth history, physical characteristics, and medical conditions in long-term su	Bruns D.	Am J Med Genet	2011
405	B	survey/interview	23154524	Ethical dilemmas associated with genetic testing: which are most commonly	McLean N, Delat	Genet Med	2013
406	B	survey/interview	23401422	Hearing from parents: the impact of receiving the diagnosis of Williams synd	Waxler JL, Chern	Am J Med Genet	2013
407	B	survey/interview		Landau-Kleffner Syndrome: An Exploration of Parent Experience of the Diagn	Daunette Lemar	ERIC: Reports	2014
408	B	survey/interview		Pediatric Neurometabolic Disorders: Medical Needs, Service Use, and Impact	Diaz, Jullianne		2014
409	B	survey/interview	24991029	The burden of Duchenne muscular dystrophy: an international, cross-section	Landfeldt E, Lind	Neurology	2014
410	B	survey/interview	29758800	An assessment of clinician and researcher needs for support in the era of gen	Savage SK, Ziniel	Per Med	2014
411	B	survey/interview	24857332	The importance of the general practitioner as an information source for pati	Teixeira E, Borlid	Patient Educ Cou	2014
412	B	survey/interview	26184072	An online monogenic diabetes discussion group: supporting families and fuel	Perrone ME, Car	Transl Res	2015
413	B	survey/interview	25846741	Next steps for birth defects research and prevention: The birth defects study	Tinker SC, Carmi	Birth Defects Res	2015
414	B	survey/interview	25011978	A survey of genetic counselors about the needs of 18-25 year olds from fami	Werner-Lin A, Ra	J Genet Couns	2015
415	B	survey/interview	27148937	Family health history reporting is sensitive to small changes in wording	Conway-Pearson	Genet Med	2016

416	B	survey/interview	27072915	Phenotype and natural history in 101 individuals with Pitt-Hopkins syndrome	de Winter CF, Ba	Orphanet J Rare	2016
417	B	survey/interview	26964543	Quantifying the burden of caregiving in Duchenne muscular dystrophy	Landfeldt E, Lind	J Neurol	2016
418	B	survey/interview	27194034	The partnership of patient advocacy groups and clinical investigators in the r	Merkel PA, Mani	Orphanet J Rare	2016
419	B	survey/interview	27484815	Primary immunodeficiency associated with chromosomal aberration - an ESI	Schatorjé E, van	Orphanet J Rare	2016
420	B	survey/interview	26856513	Stress and coping of parents caring for a child with mitochondrial disease	Senger BA, Ward	Appl Nurs Res	2016
421	B	survey/interview	28842446	A natural history study of X-linked myotubular myopathy	Amburgey K, Tsu	Neurology	2017
422	B	survey/interview	28950866	Public and patient involvement in needs assessment and social innovation: a	de Freitas C, Dos	BMC Health Serv	2017
423	B	survey/interview	28698240	Beyond hormone replacement: quality of life in women with congenital hypo	Dzemaili S, Tiem	Endocr Connect	2017
424	B	survey/interview		Usher syndrome : a phenomenological study of adults across the lifespan livi	Evans, Michelle Deborah		2017
425	B	survey/interview		The transition of patients with rare diseases between providers: the patient j	Ferrara, Lucia	International Jou	2017
426	B	survey/interview	27550467	Knowledge and Awareness of Sickle Cell Trait Among Young African America	Harrison SE, Wal	West J Nurs Res	2017
427	B	survey/interview	29250518	Whole-Genome Sequencing in Newborn Screening-Attitudes and Opinions of	Iskrov G, Ivanov	Front Public Heal	2017
428	B	survey/interview	28513617	The perceived impact of the European registration system for genetic counse	Paneque M, Mol	Eur J Hum Genet	2017
429	B	survey/interview	29637168	Rare disease: a national survey of paediatricians' experiences and needs	Zurynski Y, Gonz	BMJ Paediatr Ope	2017
430	B	survey/interview	30458817	Survey on patients with undiagnosed diseases in Japan: potential patient nu	Adachi T, Imanis	Orphanet J Rare	2018
431	B	survey/interview		MUSIC THERAPY IN RETT SYNDROME CASES	Andziule, V; Alek	SOCIETY, INTEGR	2018
432	B	survey/interview	30128673	Alone in a Crowd? Parents of Children with Rare Diseases' Experiences of Na	Baumbusch J, M	J Genet Couns	2018
433	B	survey/interview	29500624	An Internet support group for parents of children with neurofibromatosis typ	Martin S, Struem	J Community Gen	2018
434	B	survey/interview	31970210	Spinocerebellar Ataxia Patient Perceptions Regarding Reproductive Options	Cahn S, Rosen A,	Mov Disord Clin P	2019
435	B	survey/interview	30777108	An online survey on burden of illness among families with post-stem cell tran	Conner T, Cook F	Orphanet J Rare	2019
436	B	survey/interview	31801570	Cutaneous neurofibromas: patients' medical burden, current management a	Guiraud M, Bour	Orphanet J Rare	2019
437	B	survey/interview	30711301	Psychosocial issues in erythropoietic protoporphyria - the perspective of par	Naik H, Shenbag	Mol Genet Metab	2019
438	B	survey/interview	31387656	Health Care for Mitochondrial Disorders in Canada: A Survey of Physicians	Paik K, Lines MA	Can J Neurol Sci	2019
439	B	survey/interview	30696467	Assessing disease experience across the life span for individuals with osteoge	Tosi LL, Floor MK	Orphanet J Rare	2019
440	B	survey/interview	30808425	Predispositional genome sequencing in healthy adults: design, participant ch	Zoltick ES, Linder	Genome Med	2019
441	B	survey/interview	32001839	Parents' motivations, concerns and understanding of genome sequencing: a	Lewis C, Sanders	Eur J Hum Genet	2020
442	B	survey/interview	32946487	Genetic testing offer for inherited neuromuscular diseases within the EURO-	Peterlin B, Guala	PLoS One	2020
443	B	survey/interview	32128950	Understanding genetic learning needs of people affected by rare disease	Quinn L, Davis K,	J Genet Couns	2020
444	B	survey/interview	31449940	Assessment of the Information Sources and Interest in Research Collaboratio	Shalhub S, Sage L	Ann Vasc Surg	2020
445	B	survey/interview	32497379	The World Federation of Hemophilia Annual Global Survey 1999-2018	Stonebraker JS, B	Haemophilia	2020
446	B	survey/interview	33120389	Utility and Results from a Patient-Reported Online Survey in Myotonic Dystro	Wenninger S, Sta	Eur Neurol	2020
447	B	survey/interview	34964853	Expert Evaluation of Strategies to Modernize Newborn Screening in the Unit	Bailey DB Jr, Por	JAMA Netw Open	2021

448	B	survey/interview	33681659	A survey of the European Reference Network EpiCARE on clinical practice for	Baumgartner T,	Epilepsia Open	2021
449	B	survey/interview	33712027	First French study relative to preconception genetic testing: 1500 general po	Bonneau, V; Nizo	ORPHANET JOUR	2021
450	B	survey/interview	34258488	Patient-Reported Complications, Symptoms, and Experiences of Living With	Cheung M, Rylan	J Endocr Soc	2021
451	B	survey/interview	34492150	Using Online Mendelian Inheritance in Man in low- and middle-income count	de Macena Sobr	Am J Med Genet	2021
452	B	survey/interview	33537957	Patients' perception on the quality of care for multiple endocrine neoplasia d	Drewitz KP, Grey	Endocrine	2021
453	B	survey/interview	33420343	"It seems like COVID-19 now is the only disease present on Earth": living with	Halley MC, Stanl	Genet Med	2021
454	B	survey/interview	33189624	Kleefstra syndrome: Impact on parents	Haseley A, Wallis	Disabil Health J	2021
455	B	survey/interview	35386739	Impact of Covid-19 on clinical care and lived experience of systemic sclerosis	Hughes M, Pauli	J Scleroderma Re	2021
456	B	survey/interview	37181101	They've been BITTEN: reports of institutional and provider betrayal and links	Langhinrichsen-R	Ther Adv Rare Dis	2021
457	B	survey/interview	33724192	Underrepresentation of Phenotypic Variability of 16p13.11 Microduplication	Li J, Hojlo MA, Ch	J Med Internet Re	2021
458	B	survey/interview	34294091	A questionnaire-based study to comprehensively assess the status quo of rar	Li X, Liu M, Lin J,	Orphanet J Rare	2021
459	B	survey/interview	33849615	Rare disease awareness and perspectives of physicians in China: a questionn	Li X, Zhang X, Zh	Orphanet J Rare	2021
460	B	survey/interview	33602292	Clinical characteristics and burden of illness in patients with hereditary angio	Mendivil J, Murp	Orphanet J Rare	2021
461	B	survey/interview	33474836	SARS-CoV-2 pandemic in the Brazilian community of rare diseases: A patient	Schwartz IVD, Ra	Am J Med Genet	2021
462	B	survey/interview	34065803	Movement Disorders in Children with a Mitochondrial Disease: A Cross-Secti	Ticci C, Orsucci D	J Clin Med	2021
463	B	survey/interview	33764888	Transition readiness among adolescents with rare endocrine conditions	van Alewijk, L; D	ENDOCRINE CON	2021
464	B	survey/interview	34387413	Family caregivers of rare disease: A survey on health-related quality of life in	Xu J, Bao H, Qi X,	Mol Genet Geno	2021
465	B	survey/interview	35120534	Caregivers' experience of sleep management in Smith-Magenis syndrome: a	Agar G, Bissell S,	Orphanet J Rare	2022
466	B	survey/interview	36017643	The impact of fibrodysplasia ossificans progressiva (FOP) on patients and the	Al Mukaddam M	Expert Rev Pharm	2022
467	B	survey/interview	36412585	Parental Depression and Anxiety Associated with Newborn Bloodspot Screen	Boychuk NA, Mu	Int J Neonatal Scr	2022
468	B	survey/interview	36483158	Awareness and utilization of genetic testing among Hispanic and Latino adult	Christensen KD,	HGG Adv	2022
469	B	survey/interview	36089593	Advancing qualitative rare disease research methodology: a comparison of vi	Dwyer AA, Uveg	Orphanet J Rare	2022
470	B	survey/interview	36195888	Understanding caregiver descriptions of initial signs and symptoms to improv	Eichler F, Sevin C	Orphanet J Rare	2022
471	B	survey/interview	36046392	Shared decision making between patients with Fabry disease and physicians	Inagaki N, Tsuchi	Mol Genet Metab	2022
472	B	survey/interview	34954508	SYNGAP1-related developmental and epileptic encephalopathy: The impact	Lo Barco T, De G	Epilepsy Behav	2022
473	B	survey/interview	35996871	Perceptions of patient disease burden and management approaches in syste	Mesa RA, Sulliva	Cancer	2022
474	B	survey/interview	35907899	Stakeholders' views on drug development: the congenital disorders of glycos	Monticelli, M; Fr	ORPHANET JOUR	2022
475	B	survey/interview	35312266	Co-ordinated care for people affected by rare diseases: the CONCORD mixed	Morris S, Hudson	Co-ordinated car	2022
476	B	survey/interview	36307981	'Advocacy groups are the connectors': Experiences and contributions of rare	Nguyen CQ, Kari	Health Expect	2022
477	B	survey/interview	35492689	Neurobehavioral Dimensions of Prader Willi Syndrome: Relationships Betwe	O'Hora KP, Zhan	Front Psychiatry	2022
478	B	survey/interview	35467223	Caregivers' assessment of meaningful and relevant clinical outcome assessm	Porter KA, O'Neil	J Patient Rep Out	2022
479	B	survey/interview	36362876	Three-Country Snapshot of Ornithine Transcarbamylase Deficiency	Seker Yilmaz B, B	Life (Basel)	2022

480	B	survey/interview	35204645	Awareness of Nuclear Medicine Physicians in Romania Regarding the Diagno	Stan C, Mititelu	Diagnostics (Base	2022
481	B	survey/interview	35652243	Genetic testing in interstitial lung disease: An international survey	Terwiel M, Borie	Respirology	2022
482	B	survey/interview	34218493	Caregivers of individuals with Rubinstein-Taybi syndrome: Perspectives, expe	Webster J, Wiley	J Genet Couns	2022
483	B	survey/interview	35189933	The presentation, course and outcome of COVID-19 infection in people with	Whittington JE, H	Orphanet J Rare	2022
484	B	survey/interview	37284047	International Opinions on Grading of Urothelial Carcinoma: A Survey Among	Beijert IJ, Cheng	Eur Urol Open Sci	2023
485	B	survey/interview	37269059	Current practice of first-trimester ultrasound screening for structural fetal an	Bronsgeest K	Prenat Diagn	2023
486	B	survey/interview	36611195	Understanding patient and parent/caregiver perceptions on gene therapy in	Collin-Histed T, R	Orphanet J Rare	2023
487	B	survey/interview	37628409	Caring for Children with Dravet Syndrome: Exploring the Daily Challenges of	Domaradzki, J	Children	2023
488	B	survey/interview		Dads and dyads: Stress and coping when a child has retinoblastoma	Downie, R	Dissertation Abst	2023
489	B	survey/interview	37155167	Perspectives of Rare Disease Experts on Newborn Genome Sequencing	Gold NB, Adelso	JAMA Netw Open	2023
490	B	survey/interview	37406854	Lack of guidelines and translational knowledge is hindering the implementati	Koido K	Eur J Med Genet	2023
491	B	survey/interview	37261843	A prospective survey on therapeutic inertia in psoriatic arthritis (OPTI'PsA)	Lioté F	Rheumatology (O	2023
492	B	survey/interview	36669905	Management of rare movement diseases in different world regions	Painous C, Martí	Parkinsonism Rel	2023
493	B	survey/interview	37026610	Provisional practice recommendation for the management of myopathy in V	Roy B, Peck A, Ev	Ann Clin Transl N	2023
494	B	survey/interview	37348044	Health Services Needs Assessment for Retinoblastoma in Ethiopia	Sherief ST	JCO Glob Oncol	2023
495	B	survey/interview	37041611	Shared decision-making and the caregiver experience in tuberous sclerosis co	Skrobanski H, Vy	Orphanet J Rare	2023
496	B	survey/interview	36757566	The Burden of Caring for Individuals with Tuberous Sclerosis Complex (TSC) W	Skrobanski H, Vy	Pharmacoecon O	2023
497	B	survey/interview	37632685	Genetic services survey-experience of people with rare diseases and their fa	Ward AJ	J Community Gen	2023
498	B	survey/interview	37406854	Lack of guidelines and translational knowledge is hindering the implementation of psychiatric		Eur J Med Genet	2023
499	B	telemedicine	23838594	Dysmorphology at a distance: results of a web-based diagnostic service	Douzgou S, Clayt	Eur J Hum Genet	2014
500	B	telemedicine	25720320	SDHB/SDHA immunohistochemistry in pheochromocytomas and paraganglio	Papathomas TG,	Mod Pathol	2015
501	B	telemedicine	35135120	Delivering Precision Oncology in a Community Cancer Program: Results From	Powell, SE; Dib, E	JCO PRECISION O	2018
502	B	telemedicine	32568109	A Virtual Cohort Study of Individuals at Genetic Risk for Parkinson's Disease:	Schneider RB, M	J Parkinsons Dis	2020
503	C	Not relevant		The Cultivation of Grape-vines, Fruits and ornamental Plants.		Verhandlungen 7	1939
504	C	Not relevant	264077	Bilateral renal agenesis (Potter's syndrome) in two consecutive infants	Loendersloot EW	Eur J Obstet Gyne	1978
505	C	Not relevant	10630504	Chronic recurrent multifocal osteomyelitis associated with chronic inflamma	Bousvaros A, Ma	Dig Dis Sci	1999
506	C	Not relevant	10396058	Unusual immunoglobulin and T-cell receptor gene rearrangement patterns in	Szczepański T, Po	Curr Top Microbi	1999
507	C	Not relevant	29989768	Lipodystrophy Syndromes: Presentation and Treatment	Akinci B, Sahinoz	Endotext	2000
508	C	Not relevant	25905254	Diffuse Hormonal Systems	Andersson-Rolf A	Endotext	2000
509	C	Not relevant	25905240	Current Issues in the Diagnosis and Management of Adrenocortical Carcinom	Angelousi A, Kas	Endotext	2000
510	C	Not relevant	31841296	Physiology of the Pineal Gland and Melatonin	Aulinas A.	Endotext	2000
511	C	Not relevant	26844335	Pediatric Implications of Normal Insulin-GH-IGF-Axis Physiology	Bang P.	Endotext	2000

512	C	Not relevant	25905212	Thyrotropin-Secreting Pituitary Adenomas	Beck-Peccoz P, P	Endotext	2000
513	C	Not relevant	27809435	Secondary Hypertriglyceridemia	Benuck I, Wilson	Endotext	2000
514	C	Not relevant	25905404	Thyroid Hormones in Brain Development and Function	Bernal J.	Endotext	2000
515	C	Not relevant	25905322	Acromegaly	Carroll PV, Joshi	Endotext	2000
516	C	Not relevant	26561703	Hypertriglyceridemia: Pathophysiology, Role of Genetics, Consequences, and	Chait A, Subrama	Endotext	2000
517	C	Not relevant	25905285	Pituitary Tumors in Childhood	Colao A, Pirchio	Endotext	2000
518	C	Not relevant	25905295	CCKoma	de Herder WW,	Endotext	2000
519	C	Not relevant	25905215	Insulinoma	de Herder WW,	Endotext	2000
520	C	Not relevant	25905263	Somatostatinoma	de Herder WW,	Endotext	2000
521	C	Not relevant	30521183	Aggressive Pituitary Tumors and Pituitary Carcinomas	De Sousa SMC, M	Endotext	2000
522	C	Not relevant	27809442	Non-Invasive Techniques in Pediatric Dyslipidemia	Dixon DB.	Endotext	2000
523	C	Not relevant	25905384	Radiology of the Pituitary	Evanson J.	Endotext	2000
524	C	Not relevant	25905376	Pituitary and Hypothalamic Tumor Syndromes in Childhood	Gan HW, Cerbon	Endotext	2000
525	C	Not relevant	25905378	Pituitary Gigantism	George MM, Eug	Endotext	2000
526	C	Not relevant	31895524	Subcutaneous Adipose Tissue Diseases: Dercum Disease, Lipedema, Familial	Herbst KL.	Endotext	2000
527	C	Not relevant	25905331	Cryptorchidism and Hypospadias	Hutson JM.	Endotext	2000
528	C	Not relevant	33734657	Infections in Endocrinology: Tuberculosis	Jacob JJ, Paul PA	Endotext	2000
529	C	Not relevant	25905341	Carney Complex	Kaltsas G, Kanaki	Endotext	2000
530	C	Not relevant	29465932	Medical Management of the Postoperative Bariatric Surgery Patient	Kim TY, Kim S, Sc	Endotext	2000
531	C	Not relevant	25905363	TSH Receptor Mutations and Diseases	Kleinau G, Vassa	Endotext	2000
532	C	Not relevant	25905214	Overview of Endocrine Hypertension	Koch C, Papadop	Endotext	2000
533	C	Not relevant	25905391	Ambiguous Genitalia in the Newborn	Krishnan S, Meye	Endotext	2000
534	C	Not relevant	25905355	Familial or Sporadic Adrenal Hypoplasia Syndromes	Kyritsi EM, Serte	Endotext	2000
535	C	Not relevant	27809433	Familial Hypercholesterolemia	Levenson AE, de	Endotext	2000
536	C	Not relevant	27809447	Sitosterolemia in the Pediatric Population	Liebeskind A, Pet	Endotext	2000
537	C	Not relevant	30888745	Craniopharyngiomas	Lithgow K, Hamb	Endotext	2000
538	C	Not relevant	25905408	Acute and Subacute, and Riedel's Thyroiditis	Majety P, Henne	Endotext	2000
539	C	Not relevant	25905300	Pathophysiology and Treatment of Pancreatic Neuroendocrine Neoplasms (P	Mariën L, Islam	Endotext	2000
540	C	Not relevant	25905334	Osteogenesis Imperfecta	Marini JC, Dang	Endotext	2000
541	C	Not relevant	25905397	Prolactinoma Management	Molitch ME, Dru	Endotext	2000
542	C	Not relevant	25905381	Genetic Defects in Thyroid Hormone Supply	Nettore IC, Fenzi	Endotext	2000
543	C	Not relevant	25905188	Congenital Adrenal Hyperplasia	New M, Yau M, L	Endotext	2000

544	C	Not relevant	25905168	Primary Generalized Glucocorticoid Resistance or Chrousos Syndrome	Nicolaides NC, Ki	Endotext	2000
545	C	Not relevant	27809439	Cerebrotendinous Xanthomatosis	Patni N, Wilson D	Endotext	2000
546	C	Not relevant	30321013	Infections of the Hypothalamic-Pituitary Region	Pekic S, Miljic D,	Endotext	2000
547	C	Not relevant	29465925	Multiple Endocrine Neoplasia Type 1	Pieterman CRC,	Endotext	2000
548	C	Not relevant	30160871	Hypophysitis	Prete A, Salvator	Endotext	2000
549	C	Not relevant	37229239	Disorders of Adrenal Glands and Sex Development in Children: Insights from	Raizada N, Nongl	Endotext	2000
550	C	Not relevant	25905224	Testicular Cancer: Pathogenesis, Diagnosis and Management with Focus on E	Rajpert-De Meyt	Endotext	2000
551	C	Not relevant	25905418	Defects of Thyroid Hormone Transport in Serum	Refetoff S.	Endotext	2000
552	C	Not relevant	34878751	Monogenic Disorders Altering HDL Levels	Shapiro MD, Fein	Endotext	2000
553	C	Not relevant	26561704	Monogenic Disorders Causing Hypobetalipoproteinemia	Shapiro MD, Fein	Endotext	2000
554	C	Not relevant	25905262	Paget’s Disease of Bone	Singer FR.	Endotext	2000
555	C	Not relevant	25905375	Autoimmune Polyglandular Syndromes	Sperling MA, Ang	Endotext	2000
556	C	Not relevant	25905184	Familial Isolated Pituitary Adenoma	Stiles CE, Korbon	Endotext	2000
557	C	Not relevant	25905330	Gynecomastia: Etiology, Diagnosis, and Treatment	Swerdloff RS, Ng	Endotext	2000
558	C	Not relevant	25905358	Paraneoplastic Syndromes Related to Neuroendocrine Tumors	Tsoli M, Dimitria	Endotext	2000
559	C	Not relevant	29465928	Multiple Endocrine Neoplasia Type 2	van Treijen MJC,	Endotext	2000
560	C	Not relevant	26844336	Familial Hypercholesterolemia: Genes and Beyond	Warden BA, Fazi	Endotext	2000
561	C	Not relevant	12089790	[Fatal cardiomyopathy in adult in polyglucosan body disease]	Postler E, Sinder	Pathologe	2002
562	C	Not relevant	12772052	[Kava, kavapyrones and toxic liver injury]	Teschke R.	Z Gastroenterol	2003
563	C	Not relevant	15566517	Chemokine receptor 5 and primary biliary cirrhosis: a two-centre genetic ass	Baragiotta A, Flo	Liver Int	2004
564	C	Not relevant	15570530	[The case of a 86-years old woman first diagnosed with Huntington's disease	Jähnel M.	Psychiatr Prax	2004
565	C	Not relevant	15127315	Neonatal seizures in two sisters with incontinentia pigmenti	Pörksen G, Pfeiff	Neuropediatrics	2004
566	C	Not relevant		Excitement and confusion on chromosome 6q: the challenges of neuropsych	Kohn, Y.	Molecular Psychi	2005
567	C	Not relevant	16293159	A case study: identifying a new case of Wilson's disease	Noble JA.	J Am Acad Nurse	2005
568	C	Not relevant	16248849	Disconnection of language and memory in semantic dementia: a comparativ	Passmore MJ, In	Curr Alzheimer R	2005
569	C	Not relevant		Gene Mutation Found That Increases Severity of Multisystem Syndrome; Discovery Mirrors Ex		Ascribe Higher Ed	2005
570	C	Not relevant	17111776	Suspected epidermolysis bullosa simplex in a 5-week-old Nigerian: a case rep	Otaigbe BE, Datu	Niger J Med	2006
571	C	Not relevant	17582146	Attitudes of German infertile couples towards preimplantation genetic diagn	Borkenhagen A,	Hum Reprod	2007
572	C	Not relevant		Evidence of genetic effects on blood lead concentration	John B. Whitfield	Environmental H	2007
573	C	Not relevant	17881126	No association of CNR1 gene variations with susceptibility to schizophrenia	Seifert J, Ossege	Neurosci Lett	2007
574	C	Not relevant	17848583	ABCG1 is deficient in alveolar macrophages of GM-CSF knockout mice and pa	Thomassen MJ,	J Lipid Res	2007
575	C	Not relevant	17531100	Cross-study analysis of gene expression data for intermediate neuroblastom	Warnat P, Obert	BMC Cancer	2007

576	C	Not relevant	17692127	Expressed sequences tags of the anther smut fungus, Microbotryum violaceu	Yockteng R, Mar	BMC Genomics	2007
577	C	Not relevant	18506107	Linkage analysis in a large family from Pakistan with depression and a high in	Ayub, M; Irfan, M	HUMAN HEREDIT	2008
578	C	Not relevant		Astute, Assertive, and Alpha-1: Quantifying Empowerment in a Rare Genetic	Finn, S	University of Flor	2008
579	C	Not relevant		Identification of Acipenseriformes species in trade	Ludwig, A	JOURNAL OF APP	2008
580	C	Not relevant		Therapeutic potential of nuclear receptors	Schweitzer, A; Kn	EXPERT OPINION	2008
581	C	Not relevant	19807662	Discovery of selective probes and antagonists for G-protein-coupled recepto	Arterburn JB, Op	Curr Top Med Ch	2009
582	C	Not relevant	19778490	PGD for X-linked and gender-dependent disorders using a robust, flexible sin	Christofidou C, S	Reprod Biomed O	2009
583	C	Not relevant	19634033	Learning abilities and disabilities: generalist genes in early adolescence	Davis OS, Hawor	Cogn Neuropsych	2009
584	C	Not relevant	19536175	Follow-up of a major linkage peak on chromosome 1 reveals suggestive QTLs	Ehret, GB; O'Con	EUROPEAN JOUR	2009
585	C	Not relevant	19624984	Heterogeneity and atypical presentation in infantile systemic hyalinosis with	El-Kamah GY, M	Dermatol Online	2009
586	C	Not relevant	19092778	Variable number of tandem repeat polymorphisms of DRD4: re-evaluation of	Hattori, E; Nakaji	EUROPEAN JOUR	2009
587	C	Not relevant	19254051	Diagnostic potential of 3D-data-based reconstruction software: an analysis o	Holst AI, Hirschfe	Cleft Palate Crani	2009
588	C	Not relevant	19335183	A web-based study of personality, psychopathology and substance use in twi	Kendler KS, Mye	Twin Res Hum Ge	2009
589	C	Not relevant		Primary tuberculosis of the cheek skin: difficulties in diagnostic procedure.	Knezevic, P.; Kne	Macedonian Jour	2009
590	C	Not relevant		An efficient transfection method for mouse embryonic stem cells.	Ko, B. S.	Gene Therapy	2009
591	C	Not relevant	19471313	WNT10A missense mutation associated with a complete Odonto-Onycho-De	Nawaz, S; Klar, J;	EUROPEAN JOUR	2009
592	C	Not relevant		Conundrum of Autism: A Review of Its Causes and Significant Impact on the E	Nwokeafor, CU	Forum on Public	2009
593	C	Not relevant	19829181	Short QT syndrome: a review	Patel U, Pavri BB	Cardiol Rev	2009
594	C	Not relevant	19352412	Assessing the impact of FOXP1 mutations on developmental verbal dyspraxia	Vernes, SC; Mac	EUROPEAN JOUR	2009
595	C	Not relevant	20393090	Genetic screening: A primer for primary care	Andermann A, Bl	Can Fam Physicia	2010
596	C	Not relevant	20195527	Hotspots of large rare deletions in the human genome	Bradley WE, Rae	PLoS One	2010
597	C	Not relevant	19840866	Visualizing the drug target landscape	Campbell, SJ; Ga	DRUG DISCOVERY	2010
598	C	Not relevant	20378412	Two different entities of spontaneous ovarian hyperstimulation in a woman	Dieterich M, Bol	Reprod Biomed O	2010
599	C	Not relevant	20137753	Piebaldism and neurofibromatosis type 1: family report	Duarte AF, Mota	Dermatol Online	2010
600	C	Not relevant		Rare structural variants found in attention-deficit hyperactivity disorder are	Elia, J.	Molecular Psychi	2010
601	C	Not relevant	19546859	Rare structural variants found in attention-deficit hyperactivity disorder are	Elia, J; Gai, X; Xie	MOLECULAR PSY	2010
602	C	Not relevant	20300123	The pursuit of genome-wide association studies: where are we now?	Ku, CS; Loy, EY; P	JOURNAL OF HU	2010
603	C	Not relevant	20029376	The Ski proto-oncogene regulates body composition and suppresses lipogen	Leong, GM; Kee,	INTERNATIONAL	2010
604	C	Not relevant	20687088	Deferasirox for managing transfusional iron overload in people with sickle ce	Meerpohl JJ, Ant	Cochrane Databa	2010
605	C	Not relevant	21129225	Marjolin's ulcers: theories, prognostic factors and their peculiarities in spina	Nthumba, PM	WORLD JOURNAL	2010
606	C	Not relevant	20809896	Open access high throughput drug discovery in the public domain: a Mount E	Roy A, McDonald	Curr Pharm Biote	2010
607	C	Not relevant	20875327	Gorlin syndrome or basal cell nevus syndrome (BCNS): A case report	Shivaswamy KN,	Dermatol Online	2010

608	C	Not relevant	20233565	Autoimmune polyglandular syndrome-3C in a child	Turkoglu Z, Kava	Dermatol Online	2010
609	C	Not relevant	20531440	A qualitative study exploring genetic counsellors' experiences of counselling	Ulph, F; Leong, J;	EUROPEAN JOUR	2010
610	C	Not relevant	20716345	Parkinson's disease candidate gene prioritization based on expression profile	Vahedi S, Rajabia	J Biomed Sci	2010
611	C	Not relevant		The evolution of a key innovation in an experimental population of Escherich	Blount, ZD	Michigan State U	2011
612	C	Not relevant	22136859	Waardenburg Syndrome type 1: A case report	Demirci GT, Atıs	Dermatol Online	2011
613	C	Not relevant	22110609	Optimal deconvolution of transcriptional profiling data using quadratic progr	Gong T, Hartman	PLoS One	2011
614	C	Not relevant	20631810	Phylogenetic and metabolic diversity of bacteria associated with cystic fibros	Guss, AM; Roese	ISME JOURNAL	2011
615	C	Not relevant	21368916	Bohring-Opitz (Oberklaid-Danks) syndrome: clinical study, review of the liter	Hastings, R; Cob	EUROPEAN JOUR	2011
616	C	Not relevant	26302203	Molecular genetics and molecular biology of dyslexia	Kere J.	Wiley Interdiscip	2011
617	C	Not relevant	21102413	Pure erythroid leukemia: a reassessment of the entity using the 2008 World	Liu, W; Hasserjia	MODERN PATHO	2011
618	C	Not relevant	21549076	Vascular Ehlers-Danlos syndrome: a case with fatal outcome	Morais P, Mota	Dermatol Online	2011
619	C	Not relevant	22471496	Frequent incidence of double minute chromosomes in cancers, with special	Movafagh A, Mir	Asian Pac J Cance	2011
620	C	Not relevant	20972252	Rare familial 16q21 microdeletions under a linkage peak implicate cadherin 8	Pagnamenta AT,	J Med Genet	2011
621	C	Not relevant	21654723	An atlas of tissue-specific conserved coexpression for functional annotation a	Piro, RM; Ala, U;	EUROPEAN JOUR	2011
622	C	Not relevant		Notification and support for people exposed to the risk of Creutzfeldt-Jakob	Ryan, Rebecca	Cochrane Databa	2011
623	C	Not relevant	22666659	Investigational approaches for mesothelioma	Surmont, VF; van	FRONTIERS IN ON	2011
624	C	Not relevant	21505450	Nasal speech and hypothyroidism are common hallmarks of 12q15 microdel	Vergult, S; Krgov	EUROPEAN JOUR	2011
625	C	Not relevant	21264940	Family-based and population-based association studies validate PTPRD as a r	Yang Q, Li L, Yan	Mov Disord	2011
626	C	Not relevant		A better mouse model		The Science Teac	2011
627	C	Not relevant		ORAL PRESENTATION.		Pacing & Clinical	2011
628	C	Not relevant		POSTER PRESENTATIONS.		Pacing & Clinical	2011
629	C	Not relevant	22549407	A novel homozygous p.Arg527Leu LMNA mutation in two unrelated Egyptian	Al-Haggar, M; M	EUROPEAN JOUR	2012
630	C	Not relevant	21986811	Investigative pathology: leading the post-genomic revolution	Berman, DM; Bo	LABORATORY INV	2012
631	C	Not relevant	22035297	Doubling the referral rate of monogenic diabetes through a nationwide infor	Borowiec M, Fen	Clin Genet	2012
632	C	Not relevant	22178891	Visualizing the drug target landscape	Campbell, SJ; Ga	DRUG DISCOVERY	2012
633	C	Not relevant		Aggressive fibromatosis treatment - imatinib challenges.	Damjanovska, G.	Macedonian Jour	2012
634	C	Not relevant		A case of acquired hemophilia A with maxillary osteitis.	El-Graoui, O.; Fa	Macedonian Jour	2012
635	C	Not relevant	23044548	'Location, Location, Location': a spatial approach for rare variant analysis and	Fier H, Won S, Pr	Bioinformatics	2012
636	C	Not relevant	22349688	Occurrence and clinical features of epileptic and non-epileptic paroxysmal ev	Filloux FM, Carey	Eur J Med Genet	2012
637	C	Not relevant		Evaluation of the effectiveness of introducing new alleles into the gene pool	Głażewska, I.; Pr	Czech Journal of	2012
638	C	Not relevant	22385125	A waterborne outbreak with a single clone of Campylobacter jejuni in the Da	Gubbels SM, Kuh	Scand J Infect Dis	2012
639	C	Not relevant	22490388	A reinvestigation of somatic hypermethylation at the PTEN CpG island in can	Hesson LB, Pack	Biol Proced Onlin	2012

640	C	Not relevant	22547139	Microduplications disrupting the MYT1L gene (2p25.3) are associated with sc	Lee Y, Mattai A,	Psychiatr Genet	2012
641	C	Not relevant	22931863	Sibling cases of moyamoya disease having homozygous and heterozygous c.1	Miyatake, S; Tou	JOURNAL OF HU	2012
642	C	Not relevant		Actinomyces odontolyticus associated bacteremia.	Považan, A.; Vuk	Macedonian Jour	2012
643	C	Not relevant	22901769	Histological and immunohistochemical features of the spleen in persistent p	Sun P, Juskeviciu	Diagn Pathol	2012
644	C	Not relevant	22532433	Informed consent for whole genome sequencing: a qualitative analysis of pa	Tabor HK, Stock	Am J Med Genet	2012
645	C	Not relevant	22912729	SERPINA1 PiZ and PiS heterozygotes and lung function decline in the SAPALD	Thun GA, Ferraro	PLoS One	2012
646	C	Not relevant	22761566	From dynamic expression patterns to boundary formation in the presomitic	Tiedemann HB, S	PLoS Comput Bio	2012
647	C	Not relevant	23149456	The heritability of metabolic profiles in newborn twins	Alul, FY; Cook, D	HEREDITY	2013
648	C	Not relevant	24125136	The burden of illness in patients with hereditary angioedema	Banerji A.	Ann Allergy Asth	2013
649	C	Not relevant		Renal transplant in an adult with Ellis van Creveld syndrome: a case report an	Barbullushi, M.; I	Macedonian Jour	2013
650	C	Not relevant	23196650	Intraocular lymphoma: a clinical perspective	Davis, JL	EYE	2013
651	C	Not relevant	23337954	Height matters-from monogenic disorders to normal variation	Durand, C; Rapp	NATURE REVIEW	2013
652	C	Not relevant		The Development of Word-Object Associations in Typically Developing Infant	Ha, OR	University of Loui	2013
653	C	Not relevant		Congenital factor V deficiency: Moroccans cases.	Louai, O. B.; Igal	Macedonian Jour	2013
654	C	Not relevant		Model-Based Approaches to Characterize Clinical Pharmacokinetics of Atorva	Macwan, Joyce		2013
655	C	Not relevant	23914052	The role of the toxicologic pathologist in the post-genomic era(#)	Maronpot RR.	J Toxicol Pathol	2013
656	C	Not relevant	22481131	Decision analysis, economic evaluation, and newborn screening: challenges a	Prosser LA, Gros	Genet Med	2013
657	C	Not relevant	24131976	Male breast cancer: genetics, epigenetics, and ethical aspects	Rizzolo P, Silvest	Ann Oncol	2013
658	C	Not relevant	22892530	Newborn screening for cystic fibrosis: Polish 4 years' experience with CFTR se	Sobczynska-Tom	EUROPEAN JOUR	2013
659	C	Not relevant		Combination of left ventricular noncompaction and bicuspid aortic valve in 1	Spiroska, V.; Hris	Macedonian Jour	2013
660	C	Not relevant		Multiple simultaneos metastases of malignant melanoma in the stomach, sm	Vukmirovic, F.; V	Macedonian Jour	2013
661	C	Not relevant	24085434	Analysis of FOS, BTG2, and NR4A in the function of renal medullary hyperten	Wu, YB; Zang, W	GENETICS AND M	2013
662	C	Not relevant	24262189	Genetic testing of LRRK2 in Parkinson's disease: is there a clinical role?	Buhat DM, Tan E	Parkinsonism Rel	2014
663	C	Not relevant	24393595	Crohn's disease and ulcerative colitis. Occurrence, course and prognosis duri	Burisch J.	Dan Med J	2014
664	C	Not relevant	24225993	A new mutation in the C-SH2 domain of PTPN11 causes Noonan syndrome w	Carapito, R; Paul	JOURNAL OF HU	2014
665	C	Not relevant	25246116	Mutation screening of HOXA7 and HOXA9 genes in Chinese women with Mül	Chen X, Mu Y, Li	Reprod Biomed O	2014
666	C	Not relevant	24864082	[Rare clinical form of glioblastoma multiforme]	Ejma M, Walisze	Postepy Hig Med	2014
667	C	Not relevant	24573649	Paediatric thyroid surgery is safe--experiences at a tertiary surgical centre	Fahrner R, Ubers	Swiss Med Wkly	2014
668	C	Not relevant	24852389	Funding Decisions for Newborn Screening: A Comparative Review of 22 Decis	Fischer, KE; Rogo	INTERNATIONAL	2014
669	C	Not relevant	24839407	Blood gene expression profiling in pediatric systemic lupus erythematosus an	Gilbert M, Punar	Pediatr Rheumat	2014
670	C	Not relevant	25489236	Discovery of potential drugs for human-infecting H7N9 virus containing R294	He JY, Li C, Wu G	Drug Des Devel T	2014
671	C	Not relevant	24552833	CCBE1 enhances lymphangiogenesis via A disintegrin and metalloprotease w	Jeltsch M, Jha SK	Circulation	2014

672	C	Not relevant	25526005	Hailey-Hailey disease exacerbated by multiple pregnancies: case report and r	Mauzo SH, Sulit	Dermatol Online	2014
673	C	Not relevant	25419514	Lysinuric protein intolerance presenting with multiple fractures	Posey, JE; Burrag	MOLECULAR GEN	2014
674	C	Not relevant	25244167	Incidental angiofibromas prompt a diagnosis of multiple endocrine neoplasia	Roman JW, Loge	Dermatol Online	2014
675	C	Not relevant	24581601	WNT9B in 542 Chinese women with Müllerian duct abnormalities: mutation	Tang R, Dang Y,	Reprod Biomed O	2014
676	C	Not relevant	24456963	Fish odor syndrome: a case report of trimethylaminuria	Ulman CA, Trevi	Dermatol Online	2014
677	C	Not relevant	25132547	Synaptic dysregulation in a human iPS cell model of mental disorders	Wen Z, Nguyen H	Nature	2014
678	C	Not relevant	25982095	Live births in women with recurrent hydatidiform mole and two NLRP7 muta	Akoury E, Gupta	Reprod Biomed O	2015
679	C	Not relevant	26173930	GeneMatcher aids in the identification of a new malformation syndrome wit	Au PYB, You J, Ca	Hum Mutat	2015
680	C	Not relevant	25635880	Assessment of the olfactory function in Italian patients with type 3 von Wille	Cenedese V, Me	PLoS One	2015
681	C	Not relevant	26583922	"Broadband" Bioinformatics Skills Transfer with the Knowledge Transfer Prog	Chimusa ER, Mbi	PLoS Comput Bio	2015
682	C	Not relevant		Topical tacrolimus for atopic dermatitis	Cury Martins, Ja	Cochrane Databa	2015
683	C	Not relevant	26543556	Addressing health disparities in Hispanic breast cancer: accurate and inexpen	Dean M, Boland	Gigascience	2015
684	C	Not relevant	25515598	Kindler syndrome with severe mucosal involvement in a large Palestinian pe	El Hachem, M; D	EUROPEAN JOUR	2015
685	C	Not relevant	25795408	Linsitinib (OSI-906) versus placebo for patients with locally advanced or meta	Fassnacht M, Be	Lancet Oncol	2015
686	C	Not relevant		Mutation analysis of GABAergic neuroinhibitory genes in childhood genetic g	Hunt-Jones, Charlotte Amy		2015
687	C	Not relevant	26384833	Fraser syndrome with laryngeal webs: Report of two cases and a review of th	Izadi F, Ahmadi A	Int J Pediatr Otor	2015
688	C	Not relevant	25667274	Molecular profiling and targeted therapy for advanced thoracic malignancies	Lopez-Chavez A,	J Clin Oncol	2015
689	C	Not relevant	26220823	Matching two independent cohorts validates DPH1 as a gene responsible for	Loucks CM, Parb	Hum Mutat	2015
690	C	Not relevant	25928053	Primigravida with Bernard-Soulier Syndrome: a case report	Macêdo MB, Brit	BMC Res Notes	2015
691	C	Not relevant	25726555	Diagnosing α1-antitrypsin deficiency: how to improve the current algorithm	McElvaney NG.	Eur Respir Rev	2015
692	C	Not relevant	26437281	A 7-year-old with indurated skin and unilateral progressive joint immobility:	Ogunmakin K, Va	Dermatol Online	2015
693	C	Not relevant		The challenges, adaptations, and areas of need that confront adult men with	Rolstad, Erik Bru	Dissertation Abst	2015
694	C	Not relevant	26648932	The Scion/Rootstock Genotypes and Habitats Affect Arbuscular Mycorrhizal F	Song F, Pan Z, Ba	Front Microbiol	2015
695	C	Not relevant	25392409	GEM2Net: from gene expression modeling to -omics networks, a new CATdb	Zaag R, Tamby JP	Nucleic Acids Res	2015
696	C	Not relevant	26652000	Population genomics of intrapatient HIV-1 evolution	Zanini F, Brodin J	Elife	2015
697	C	Not relevant	26677414	Spinocerebellar ataxia 28: a novel AFG3L2 mutation in a German family with	Zühlke C, Mikat	Cerebellum Ataxi	2015
698	C	Not relevant	27560481	Opportunities and technical challenges in next-generation sequencing for dia	Bacchelli C, Willi	Expert Rev Mol D	2016
699	C	Not relevant	26874361	Spontaneous fertility and pregnancy outcomes amongst 480 women with Tu	Bernard V, Dona	Hum Reprod	2016
700	C	Not relevant	27267192	Progeria and the early aging in children: a case report	Carvalho VO, Cel	Dermatol Online	2016
701	C	Not relevant	26925973	Clinical application of genomic profiling to find druggable targets for adolesc	Cha S, Lee J, Shin	BMC Cancer	2016
702	C	Not relevant		The natural history of pancreatic acinar cell cystadenoma: Is resection better	Darcy, DG; Jan, D	JOURNAL OF PED	2016
703	C	Not relevant		The Pathogenesis of Spontaneous Autoimmune Peripheral Polyneuropathy :	Gadsden, BJ	Michigan State U	2016

704	C	Not relevant	27354474	Shorter Remission Telomere Length Predicts Delayed Neutrophil Recovery Af	Gerbing RB, Alon	J Clin Oncol	2016
705	C	Not relevant	27881174	Juvenile arthritis caused by a novel FAMIN (LACC1) mutation in two children	Kallinich T, Thor	Pediatr Rheumat	2016
706	C	Not relevant	27211562	Phenotypically distinct subtypes of psychosis accompany novel or rare varian	Kranz, TM; Berns	EBIOMEDICINE	2016
707	C	Not relevant	26483095	Health-related quality of life in patients with Duchenne muscular dystrophy:	Landfeldt E, Lind	Dev Med Child N	2016
708	C	Not relevant	27114598	Clinical and Genetic Risk Factors for Acute Pancreatitis in Patients With Acute	Liu C, Yang W, D	J Clin Oncol	2016
709	C	Not relevant		Chromosome Structural Alteration an Unusual Abnormality Characterizing H	Movafagh, Abolf	Novelty in Biome	2016
710	C	Not relevant	26522496	The contribution of the androgen receptor (AR) in human spatial learning an	Mueller SC, Verw	Horm Behav	2016
711	C	Not relevant	26739172	A human macrophage-hepatocyte co-culture model for comparative studies	Rennert K, Otto	BMC Microbiol	2016
712	C	Not relevant	26851350	The Italian pilot external quality assessment program for cystic fibrosis sweat	Salvatore M, Flo	Clin Biochem	2016
713	C	Not relevant	27454254	Abstracts from the 3rd International Genomic Medicine Conference (3rd IGM	Shay JW, Homm	BMC Genomics	2016
714	C	Not relevant	27294413	Human genome meeting 2016 : Houston, TX, USA. 28 February - 2 March 20	Srivastava AK, W	Hum Genomics	2016
715	C	Not relevant	27623166	Sequence-Based Prediction of Protein-Carbohydrate Binding Sites Using Supp	Taherzadeh G, Z	J Chem Inf Model	2016
716	C	Not relevant	27639823	Genetic Factors of the Disease Course After Sepsis: Rare Deleterious Variants	Taudien, S; Lauss	EBIOMEDICINE	2016
717	C	Not relevant	27905446	Genomic evolution and chemoresistance in germ-cell tumours	Taylor-Weiner A,	Nature	2016
718	C	Not relevant	27473762	Nosological delineation of congenital ocular motor apraxia type Cogan: an ob	Wente S, Schröd	Orphanet J Rare	2016
719	C	Not relevant	26809507	Integrated genome-scale analysis of the transcriptional regulatory landscape	Wilson NK, Scho	Blood	2016
720	C	Not relevant		4th Pediatric Allergy and Asthma Meeting (PAAM).	Yavuz, S. Tolga	Clinical & Transla	2016
721	C	Not relevant	26288127	Genetic Architecture for Human Aggression: A Study of Gene-Phenotype Rel	Zhang-James, Y;	AMERICAN JOUR	2016
722	C	Not relevant		Detecting congenital malformations - Lessons learned from the Mpepu study	Ajibola G	Plos one	2017
723	C	Not relevant	28494185	Functional analysis of rare variants in mismatch repair proteins augments res	Arora, S; Huwe,	CANCER BIOLOGY	2017
724	C	Not relevant		Iran Diabetes Research Roadmap (IDRR) Study; Knowledge Gap in Genetic Re	Bandarian, F; Om	IRANIAN JOURNA	2017
725	C	Not relevant	28512024	Repurposing of Proton Pump Inhibitors as first identified small molecule inhi	Bi Y, Might M, Va	Bioorg Med Chem	2017
726	C	Not relevant	27854358	Causes of death in Prader-Willi syndrome: Prader-Willi Syndrome Association	Butler MG, Manz	Genet Med	2017
727	C	Not relevant	28961772	Reference genome assessment from a population scale perspective: an accu	Carbonell-Caball	Bioinformatics	2017
728	C	Not relevant	28073796	Comparative efficacy and tolerability of pharmacological interventions for at	Cortese S, Adam	BMJ Open	2017
729	C	Not relevant	28620005	Cancer and Central Nervous System Tumor Surveillance in Pediatric Neurofib	Evans DGR, Salva	Clin Cancer Res	2017
730	C	Not relevant	27831545	Neuroimaging findings in Mowat-Wilson syndrome: a study of 54 patients	Garavelli L, Ivano	Genet Med	2017
731	C	Not relevant	27993616	Quantitative multi-target RNA profiling in Epstein-Barr virus infected tumor c	Greijer, AE; Ram	JOURNAL OF VIR	2017
732	C	Not relevant	28785338	Subungual Squamous Cell Carcinoma Associated with Long Standing Onycho	Grigorov Y, Philip	Open Access Mac	2017
733	C	Not relevant	28931371	VISMapper: ultra-fast exhaustive cartography of viral insertion sites for gene	Juanes JM, Galle	BMC Bioinformat	2017
734	C	Not relevant	28241850	Accurate and equitable medical genomic analysis requires an understanding	Kessler MD, O'Co	Genome Biol	2017
735	C	Not relevant	28151489	Molecular diagnosis of PIK3CA-related overgrowth spectrum (PROS) in 162 p	Kuentz P, St-Ong	Genet Med	2017

736	C	Not relevant	29274129	Newborn screening for galactosaemia	Lak R, Yazdizade	Cochrane Databa	2017
737	C	Not relevant		Combination of deep phenotyping and targeted next generation sequencing	Louka E	Haematologica. C	2017
738	C	Not relevant	29604918	Emerging Concepts and Methodologies in Cancer Biomarker Discovery	Lu M, Zhang J, Zh	Crit Rev Oncog	2017
739	C	Not relevant	28728956	Ataluren in patients with nonsense mutation Duchenne muscular dystrophy	McDonald CM, C	Lancet	2017
740	C	Not relevant	28406489	Consensus recommendation for a diagnostic guideline for acid sphingomyeli	McGovern MM,	Genet Med	2017
741	C	Not relevant	27801985	Global regulatory framework for production and marketing of crops biofortif	Mejia LA, Dary O	Ann N Y Acad Sci	2017
742	C	Not relevant	25458638	Infantile Systemic Hyalinosis Complicated with Right Atrial Thrombus and Pe	Mohamed, S; Ah	PEDIATRICS AND	2017
743	C	Not relevant	28945252	Enhancer connectome in primary human cells identifies target genes of disea	Mumbach MR, S	Nat Genet	2017
744	C	Not relevant	27811861	A survey of current practices for genomic sequencing test interpretation and	O'Daniel JM, Mc	Genet Med	2017
745	C	Not relevant	29054256	Drug-target interaction prediction: A Bayesian ranking approach	Peska L, Buza K,	Comput Methods	2017
746	C	Not relevant	29087379	Changes in bacterioplankton community structure during early lake ontogen	Peter H, Jeppese	ISME J	2017
747	C	Not relevant	29226142	Computational Exploration for Lead Compounds That Can Reverse the Nucle	Rampogu S, Bae	Biomed Res Int	2017
748	C	Not relevant	29297860	Italian external quality assessment program for cystic fibrosis sweat chloride	Salvatore M, Flo	Ann Ist Super San	2017
749	C	Not relevant		Practical applications of genomics to natural product discovery and biosynth	Schwalen, Christopher Joseph		2017
750	C	Not relevant	28061752	Enumerateblood - an R package to estimate the cellular composition of whol	Shannon CP, Bals	BMC Genomics	2017
751	C	Not relevant	28385611	Description of the EuroTARGET cohort: A European collaborative project on	van der Zanden L	Urol Oncol	2017
752	C	Not relevant	28818704	Orphan Drug Regulation: A missed opportunity for children and adolescents	Vassal G, Kearns	Eur J Cancer	2017
753	C	Not relevant	27657686	The collective impact of rare diseases in Western Australia: an estimate usin	Walker CE, Mahe	Genet Med	2017
754	C	Not relevant	28702326	Rev-Erb co-regulates muscle regeneration via tethered interaction with the N	Welch, RD; Guo,	MOLECULAR MET	2017
755	C	Not relevant	28856090	A case of relapsed lung abscess caused by Eubacterium brachy infection follo	Yamakawa, H; H	RESPIRATORY ME	2017
756	C	Not relevant	27404900	Multiclass imbalance learning: Improving classification of pediatric brain tum	Zarinabad N, Wil	Magn Reson Med	2017
757	C	Not relevant		FDA Approves Brineura(TM) (cerliponase alfa) for the Treatment of CLN2 Disease, a Form of B		PR Newswire	2017
758	C	Not relevant		Key pre-clinical data on oculopharyngeal muscular dystrophy (OPMD) published in Nature Co		PR Newswire	2017
759	C	Not relevant	29332637	Pilot-RCT of an integrative group therapy for patients with refractory irritable	Berens S, Stroe-K	J Psychosom Res	2018
760	C	Not relevant	29217836	Drug development for neurodevelopmental disorders: lessons learned from	Berry-Kravis EM,	Nat Rev Drug Dis	2018
761	C	Not relevant	29861534	Understanding activity participation among individuals with Wolfram Syndro	Bumpus E, Hersh	Br J Occup Ther	2018
762	C	Not relevant	30541556	"Omics" data integration and functional analyses link Enoyl-CoA hydratase, s	Campbell NV, W	BMC Med Genom	2018
763	C	Not relevant	29288495	PanCancer insights from The Cancer Genome Atlas: the pathologist's perspec	Cooper LA, Demi	J Pathol	2018
764	C	Not relevant	29988390	Peculiarities of Precocious Puberty in Boys and Girls With McCune-Albright S	Corica D, Aversa	Front Endocrinol	2018
765	C	Not relevant	29708978	Frequency and distribution of 152 genetic disease variants in over 100,000 m	Donner J, Anders	PLoS Genet	2018
766	C	Not relevant	29350590	Human genetics and molecular mechanisms of vein of Galen malformation	Duran D, Karschn	J Neurosurg Pedi	2018
767	C	Not relevant	29240009	Gastrointestinal Symptoms in Children and Adolescents With Neurofibromat	Ejerskov C, Krog	J Pediatr Gastroe	2018

768	C	Not relevant	29337599	Pearson syndrome	Farruggia P, Di M	Expert Rev Hema	2018
769	C	Not relevant	30048469	One million dog vaccinations recorded on mHealth innovation used to direct	Gibson AD, Maze	PLoS One	2018
770	C	Not relevant	29203382	Thirteen years' experience of 893 PGD cycles for monogenic disorders in a pu	Girardet A, Ishm	Reprod Biomed O	2018
771	C	Not relevant	30369044	Severe bleeding with subclinical oculocutaneous albinism in a patient with a	Han CG, O'Brien	Am J Med Genet	2018
772	C	Not relevant	28780023	Shared ACVR1 mutations in FOP and DIPG: Opportunities and challenges in e	Han, HJ; Jain, P;	BONE	2018
773	C	Not relevant	29678421	Growth hormone receptor-deficient pigs resemble the pathophysiology of h	Hinrichs, A; Kessl	MOLECULAR MET	2018
774	C	Not relevant	29032440	Vasculitis and vasculitis-like manifestations in monogenic autoinflammatory	Jain A, Misra DP,	Rheumatol Int	2018
775	C	Not relevant	30060766	Detection of variants in dystroglycanopathy-associated genes through the ap	Johnson K, Berto	Skelet Muscle	2018
776	C	Not relevant	30046452	Opportunities and Challenges for Genetic Studies of End-Stage Renal Disease	Kalatharan V, Le	Can J Kidney Heal	2018
777	C	Not relevant	30560012	A compilation of national plans, policies and government actions for rare dis	Khosla, N; Valde	INTRACTABLE & R	2018
778	C	Not relevant	29466827	Psychometric analysis of the pediatric quality of life inventory 3.0 neuromusc	Landfeldt E, May	Muscle Nerve	2018
779	C	Not relevant	30257684	Successful use of ofatumumab in two cases of early-onset juvenile SLE with t	Lei L, Muhamma	Pediatr Rheumat	2018
780	C	Not relevant	30068317	Genome-wide association study of lung function and clinical implication in he	Li X, Ortega VE, A	BMC Med Genet	2018
781	C	Not relevant	29384847	Identification of key candidate genes and pathways in hepatitis B virus-assoc	Lin H, Zhang Q, L	Medicine (Baltim	2018
782	C	Not relevant	30280610	STAT1 and STAT3 mutations: important lessons for clinical immunologists	Olbrich P, Freem	Expert Rev Clin Im	2018
783	C	Not relevant	29633139	Automating Collection of Pain-Related Patient-Reported Outcomes to Enhan	Owen-Smith A, M	J Gen Intern Med	2018
784	C	Not relevant	29335879	Current Approaches and New Developments in the Pharmacological Manage	Quezada J, Coffm	CNS Drugs	2018
785	C	Not relevant		An In-Silico Approach for the Prediction of miRNAs in Merkel Cell Polyoma Vi	Rahman, G; Mia	ADVANCEMENTS	2018
786	C	Not relevant	30096299	Molecular Diversity and Specializations among the Cells of the Adult Mouse	Saunders A, Mac	Cell	2018
787	C	Not relevant	29456579	Splenic cyst as a rare cause of fetal abdominal cystic mass: A multicenter seri	Sepulveda W, Oc	Ultrasound	2018
788	C	Not relevant	30098061	MelaNostrum: a consensus questionnaire of standardized epidemiologic and	Stratigos AJ, Farg	J Eur Acad Derma	2018
789	C	Not relevant	29538758	Using genes to triangulate the pathophysiology of granulomatous autoinflam	Szymanski AM, O	Int Immunol	2018
790	C	Not relevant	29476647	Prenatal diagnosis in rare bleeding disorders-An unresolved issue?	Tabibian S, Sham	Int J Lab Hematol	2018
791	C	Not relevant		Medium sized congenital melanocytic nevus with suspected progression to m	Tchernev, G.; Dz	Open Access Mac	2018
792	C	Not relevant		Eruptive basaliomas: "why we have to perform surgery? "Or said otherwise:	Temelkova, I.; M	Open Access Mac	2018
793	C	Not relevant	29739461	Clinical providers' experiences with returning results from genomic sequenci	Wynn J, Lewis K,	BMC Med Genom	2018
794	C	Not relevant	30220148	[Screening of the SETX gene in sporadic amyotrophic lateral sclerosis patient	Zhang H, Liang JL	Zhonghua Yi Xue	2018
795	C	Not relevant		CONSIDERATIONS ON PAROXYSMAL NOCTURNAL HEMOGLOBINURIA (PNH):	Araujo, FC; da Sil	REVISTA CIENTIFI	2019
796	C	Not relevant	32099441	Identification of Candidate Genes and Therapeutic Agents for Light Chain Am	Bai W, Wang H,	Pharmgenomics P	2019
797	C	Not relevant		Supporting Medical Decisions for Treating Rare Diseases Through Genetic Pr	Bakurov, I; Caste	APPLICATIONS OF	2019
798	C	Not relevant	31046790	Disease course and treatment effects of a JAK inhibitor in a patient with CAN	Boyadzhiev M, M	Pediatr Rheumat	2019
799	C	Not relevant	30606745	Leveraging Evidence-Based Public Policy and Advocacy to Advance Newborn	Bronstein MG, P	Pediatrics	2019

800	C	Not relevant	31291964	Testicular ischemia in deficiency of adenosine deaminase 2 (DADA2)	Clarke K, Campb	Pediatr Rheumat	2019	
801	C	Not relevant	31687649	Effect of Bisphosphonates on Function and Mobility Among Children With Os	Constantino CS,	JBMR Plus	2019	
802	C	Not relevant		Australian Group on Antimicrobial Resistance (AGAR) Australian Staphylococ	Coombs, G. W.;	Communicable D	2019	
803	C	Not relevant	31150784	Computational de-orphanization of the olive oil biophenol oleacein: Discover	Cuyàs E, Castillo	Food Chem Toxic	2019	
804	C	Not relevant	31242245	Genetic analysis of Ghanaian G1P[8] and G9P[8] rotavirus A strains reveals th	Damanka SA, Ag	PLoS One	2019	
805	C	Not relevant	31330498	Immune checkpoint inhibitors and type 1 diabetes mellitus: a case report an	de Filette, JMK;	EUROPEAN JOUR	2019	
806	C	Not relevant	31342592	Ten years of DICER1 mutations: Provenance, distribution, and associated phe	de Kock L, Wu M	Hum Mutat	2019	
807	C	Not relevant		SA102 - A COGNITIVE AND MOLECULAR ANALYSIS OF SDCCAG8, A SCHIZOPH	Flynn, Mairead	European Neurop	2019	
808	C	Not relevant		Prediction of causative genes in inherited retinal disorders from spectral-dom	Fujinami YY	Investigative oph	2019	
809	C	Not relevant	30576415	Very low-depth whole-genome sequencing in complex trait association studi	Gilly A, Southam	Bioinformatics	2019	
810	C	Not relevant	31498321	In Vivo Functional Study of Disease-associated Rare Human Variants Using Dr	Harnish JM, Deal	J Vis Exp	2019	
811	C	Not relevant	30638886	Exploring the impact of Osteogenesis Imperfecta on families: A mixed-metho	Hill M, Lewis C, R	Disabil Health J	2019	
812	C	Not relevant	30595373	Associations of Mitochondrial and Nuclear Mitochondrial Variants and Genes	Kraja AT, Liu C, F	Am J Hum Genet	2019	
813	C	Not relevant	30773598	Treatment and long-term outcome in primary distal renal tubular acidosis	Lopez-Garcia SC,	Nephrol Dial Tran	2019	
814	C	Not relevant		11 - SYSTEMATIC RECONSTRUCTION OF AUTISM BIOLOGY WITH MULTI-LEVE	Luo, Weijun	European Neurop	2019	
815	C	Not relevant	31320697	Transcriptome analysis and codominant markers development in caper, a dr	Mercati, F; Fonta	SCIENTIFIC REPO	2019	
816	C	Not relevant		Telomere Disclosure and Impact on Psychological Distress and Health Behavi	NCT04104386	https://clinicaltri	2019	
817	C	Not relevant		Supply-side measures improving food environments promoting neglected an	Raneri, J. E.; Pad	UNSCN News	2019	
818	C	Not relevant	31819345	Identification of Targetable Pathways in Oral Cancer Patients via Random For	Schomberg J.	Cancer Inform	2019	
819	C	Not relevant	30454948	Last cases of rubella and congenital rubella syndrome in Spain, 1997-2016: T	Seppala, EM; Lop	VACCINE	2019	
820	C	Not relevant	30626441	An ontological foundation for ocular phenotypes and rare eye diseases	Sergouniotis PI,	Orphanet J Rare	2019	
821	C	Not relevant	31159889	Testing criteria for 22q11.2 deletion syndrome: preliminary results of a low c	Sgardioli IC, Paol	Orphanet J Rare	2019	
822	C	Not relevant	31286971	Whole exome sequencing approach to childhood onset familial erythroderm	Signa S, Campion	Pediatr Rheumat	2019	
823	C	Not relevant	30940883	Identification of Allosteric Inhibitors against Active Caspase-6	Tubeleviciute-Ay	Sci Rep	2019	
824	C	Not relevant	30689875	Proof of concept for quantitative urine NMR metabolomics pipeline for large	Tynkkynen T, Wa	Int J Epidemiol	2019	
825	C	Not relevant		The impact of climate on the winter strategies of insects.	van Baaren, Joan	Comptes Rendus	2019	
826	C	Not relevant	31626773	Genetic Control of Expression and Splicing in Developing Human Brain Inform	Walker RL, Rama	Cell	2019	
827	C	Not relevant	31835584	An Integrated Pan-Cancer Analysis and Structure-Based Virtual Screening of	Wang Y, Wang X	Int J Mol Sci	2019	
828	C	Not relevant	31850125	Successful Treatment of a Widespread Pemphigus Chronicus Familiaris (Haile	Wollina U, Hanse	Open Access Mac	2019	
829	C	Not relevant	30703280	Ectodermal dysplasias: Classification and organization by phenotype, genoty	Wright JT, Fete M	Am J Med Genet	2019	
830	C	Not relevant	31000350	Mutational analysis of theFAM175A gene in patients with premature ovarian	Xu X, Zhang Y, Zh	Reprod Biomed O	2019	
831	C	Not relevant		Routing algorithms for optimisation of transportation systems	Ancele, Y	Liverpool John M	2020	

832	C	Not relevant		Seasonal filarial infections and their black fly vectors in Chiang Mai Province,	Aupalee, K.; Sae	Pathogens	2020
833	C	Not relevant	32941715	Intravascular lymphoma presenting with paraneoplastic syndrome	Cenk H, Sarac G,	Dermatol Online	2020
834	C	Not relevant	32471521	Searching for a Bacteriophage Lysin to Treat Corynebacterium bovis in Immu	Cheleuitte-Nieve	Comp Med	2020
835	C	Not relevant		Australian Group on Antimicrobial Resistance (AGAR) Australian Staphylococ	Coombs, G. W.;	Communicable D	2020
836	C	Not relevant	30116028	Identification of ADHD risk genes in extended pedigrees by combining linkag	Corominas J, Klei	Mol Psychiatry	2020
837	C	Not relevant		Sickle cell anaemia in vaso-occlusive crisis and acute fatty liver of pregnancy:	Ezeanochie, M. C	Ghana Medical Jo	2020
838	C	Not relevant	32006723	Primary Immunodeficiency Diseases and Bacillus Calmette-Guérin (BCG)-Vac	Fekrvand S, Yazd	J Allergy Clin Imm	2020
839	C	Not relevant	31821114	Effective Altruism as an Ethical Lens on Research Priorities	Garrett KA, Alcal	Phytopathology	2020
840	C	Not relevant	32714614	Microarray-Based Comparative Genomic Hybridization, Multiplex Ligation-D	Glaeser AB, Diniz	J Pediatr Genet	2020
841	C	Not relevant	32219041	Identification of key gene modules and hub genes of human mantle cell lymp	Guo D, Wang H,	PeerJ	2020
842	C	Not relevant	32154507	Adults with systemic lupus exhibit distinct molecular phenotypes in a cross-s	Guthridge, JM; L	ECLINICALMEDICI	2020
843	C	Not relevant	32428713	Clinical, Immunological, and Genetic Features in Patients with Immune Dysre	Jamee M, Zaki-D	J Allergy Clin Imm	2020
844	C	Not relevant	33178111	Dissecting the Genetic and Etiological Causes of Primary Microcephaly	Jean F, Stuart A,	Front Neurol	2020
845	C	Not relevant	32917212	A case of Myhre syndrome mimicking juvenile scleroderma	Jensen B, James	Pediatr Rheumat	2020
846	C	Not relevant		A Review on the Reappearance of Crimean-Congo Hemorrhagic Fever, a Tick	Kassiri, H; Dehgh	ENTOMOLOGY A	2020
847	C	Not relevant	31999492	Molecular Diagnosis and Identification of Genetic Variants Underlying Distal	Khan N, Akhtar N	Genet Test Mol B	2020
848	C	Not relevant		Drug repurposing approach for the identification and designing of potential E	Kumar, A; Rathi,	STRUCTURAL CHE	2020
849	C	Not relevant	32567677	Newborn screening for galactosaemia	Lak R, Yazdizade	Cochrane Databa	2020
850	C	Not relevant	31685420	Proceedings of the 2019 Santa Fe Bone Symposium: New Concepts in the Ca	Lewiecki EM, Bil	J Clin Densitom	2020
851	C	Not relevant	32849793	Screening and Identification of Therapeutic Targets for Pulmonary Arterial H	Li Q, Meng L, Liu	Front Genet	2020
852	C	Not relevant	33367818	Treatment and long-term outcome in primary nephrogenic diabetes insipidu	Lopez-Garcia SC,	Nephrol Dial Tran	2020
853	C	Not relevant	32535968	Association between X-ray repair cross-complementing group 1 Arg399Gln p	Lv MQ, Li YX, Ge	Andrologia	2020
854	C	Not relevant	32460895	The Medical Genome Initiative: moving whole-genome sequencing for rare d	Marshall CR, Bick	Genome Med	2020
855	C	Not relevant		In Silico Evaluation of the ATP7B Protein: Insights from the Role of Rare Codo	Mortazavi, M; Ba	CURRENT PROTE	2020
856	C	Not relevant	33353000	Energy Metabolism Disturbances in Cell Models of PARK2 CNV Carriers with	Palladino VS, Chi	J Clin Med	2020
857	C	Not relevant	31863858	"Shanghuo" increases disease susceptibility: Modern significance of an old TC	Pan MH, Zhu SR,	J Ethnopharmaco	2020
858	C	Not relevant	32653837	Total gastrectomy for the treatment of Menetrier's disease persistent to me	Parianos, C; Agg	INTERNATIONAL	2020
859	C	Not relevant	32827285	West syndrome: a comprehensive review	Pavone P, Polizzi	Neurol Sci	2020
860	C	Not relevant		HSEAT: A Tool for Plant Heat Shock Element Analysis, Motif Identification an	Qazi, SR; Ul Haq,	CURRENT BIOINF	2020
861	C	Not relevant	32384786	The 15q11.2 BP1-BP2 Microdeletion (Burnside-Butler) Syndrome: In Silico An	Rafi SK, Butler M	Int J Mol Sci	2020
862	C	Not relevant	32980702	Management of neonatal retro-auricular embryonal rhabdomyosarcoma - Ca	Roubaud, MJC; P	INTERNATIONAL	2020
863	C	Not relevant	32375358	The Italian External Quality Assessment Program for Cystic Fibrosis Sweat Ch	Salvatore M, Am	Int J Environ Res	2020

864	C	Not relevant	32947040	Environmental risk factors associated with ANCA associated vasculitis: A syst	Scott J, Hartnett	Autoimmun Rev	2020
865	C	Not relevant	32431715	Clinical, Immunological, and Genetic Features in 49 Patients With ZAP-70 Def	Sharifinejad N, Ja	Front Immunol	2020
866	C	Not relevant		Far Eastern Scarlet-like Fever is a special clinical and epidemic manifestation	Somova, L. M.; A	Pathogens	2020
867	C	Not relevant	32415173	ROR2 suppresses metastasis of prostate cancer via regulation of miR-199a-5	Tseng JC, Huang	Cell Death Dis	2020
868	C	Not relevant	33391274	What Works When Treating Granulomatous Disease in Genetically Undefine	van Stigt AC, Dik	Front Immunol	2020
869	C	Not relevant	34164613	Nasal chondromesenchymal hamartomas in a cohort with pathogenic germli	Vasta LM, Nichol	Rhinol Online	2020
870	C	Not relevant	32822230	Treatment of Refractory Convulsive Status Epilepticus: A Comprehensive Rev	Vossler DG, Bain	Epilepsy Curr	2020
871	C	Not relevant	33015062	A Mutation in VWA1, Encoding von Willebrand Factor A Domain-Containing	Wang Y, Ping L, L	Front Cell Dev Bio	2020
872	C	Not relevant	32243819	Co-localization between Sequence Constraint and Epigenomic Information Im	Xu D, Wang C, Ki	Am J Hum Genet	2020
873	C	Not relevant	31794146	Low frequency of mutation of epidermal growth factor receptor (EGFR) and	Yin K, Feng HB, L	Thorac Cancer	2020
874	C	Not relevant	32284612	Therapeutic base editing of human hematopoietic stem cells	Zeng J, Wu Y, Re	Nat Med	2020
875	C	Not relevant	33228694	RNA sequencing analysis reveals increased expression of interferon signaling	Zhytnik L, Maasa	BMC Med Genom	2020
876	C	Not relevant		Swim coach & wife share daughter's transplant story.		UWIRE Text	2020
877	C	Not relevant	34430209	Use of pegvaliase in the management of phenylketonuria: Case series of earl	Adams D, Anders	Mol Genet Metab	2021
878	C	Not relevant		Epidemiological aspects of breast cancer.	Aimukhambetov	Science & H	2021
879	C	Not relevant		Host genetic factors determining COVID-19 susceptibility and severity.	Aitkulova, A. M.;	Science & H	2021
880	C	Not relevant	34621630	Validation of the Greek version of the Problematic Internet Use Questionnair	Aivali P, Efthymi	EMBnet J	2021
881	C	Not relevant	34063499	Children's Preferences for Oral Dosage Forms and Their Involvement in Form	Alessandrini E, B	Pharmaceutics	2021
882	C	Not relevant	34678156	A prospective prostate cancer screening programme for men with pathogeni	Bancroft, EK; Pag	LANCET ONCOLO	2021
883	C	Not relevant	33326162	Insights into multiple sclerosis-associated uveitis: a scoping review	Casselman P, Cas	Acta Ophthalmol	2021
884	C	Not relevant		Dural venous sinus thrombosis: a case report.	Chaurasia, D.; Ya	JNMA, Journal of	2021
885	C	Not relevant		Childhood neurodevelopmental disorders and maternal hypertensive disord	Chen, Kuan-Ru	Developmental M	2021
886	C	Not relevant	33785490	Study protocol of the global Effisayil 1 Phase II, multicentre, randomised, dou	Choon SE, Lebwo	BMJ Open	2021
887	C	Not relevant	34763726	"I took it off most of the time 'cause I felt comfortable": unmasking, trusted	Dariotis JK, Sloan	J Med Case Rep	2021
888	C	Not relevant	34503678	Phenotypic Expression and Outcomes in Individuals With Rare Genetic Varian	de Marvao, A; M	JOURNAL OF THE	2021
889	C	Not relevant		Characterization of cancer immune landscape in primary central nervous sys	Fei Fei; Wang Ka	Erciyes Medical J	2021
890	C	Not relevant	34905009	Safety and Efficacy of Acetyl-DL-Leucine in Certain Types of Cerebellar Ataxia	Feil K, Adrion C,	JAMA Netw Open	2021
891	C	Not relevant	33326660	Whole-exome sequencing of non-BRCA1/BRCA2 mutation carrier cases at hig	Felicio PS, Grasel	Hum Mutat	2021
892	C	Not relevant	34031609	BAR-Seq clonal tracking of gene-edited cells	Ferrari S, Beretta	Nat Protoc	2021
893	C	Not relevant	32845020	Quantitative retrospective natural history modeling for orphan drug develop	Garbade SF, Ziel	J Inherit Metab D	2021
894	C	Not relevant	33716826	Yale Global Tic Severity Scale (YGTSS): Psychometric Quality of the Gold Stan	Haas M, Jakubov	Front Psychiatry	2021
895	C	Not relevant	33839563	Epilepsy in patients with familial hemiplegic migraine	Hasırcı Bayır BR,	Seizure	2021

896	C	Not relevant	34803695	Identification of Hypoxia Induced Metabolism Associated Genes in Pulmonar	He YY, Xie XM, Z	Front Pharmacol	2021
897	C	Not relevant		A rare duplication in the PLAG1 gene: a case of neonatal diabetes.	Hekimoğlu, B.; Ç	Erciyes Medical J	2021
898	C	Not relevant		Human babesiosis in Europe.	Hildebrandt, A.;	Pathogens	2021
899	C	Not relevant	34867966	Immune Dysregulation in Patients With Chromosome 18q Deletions-Searchin	Hogendorf A, Zie	Front Immunol	2021
900	C	Not relevant		Acute pancreatitis and acute recurrent pancreatitis: investigation of clinical a	Hoşnut, F. Ö.; Şa	Izmir Dr. Behçet	2021
901	C	Not relevant	33824924	Evolution of the liver biopsy and its future	Jain D, Torres R,	Transl Gastroente	2021
902	C	Not relevant	34064706	Pancreatic Disorders in Children with Inflammatory Bowel Disease	Jakimiec P, Zdan	Medicina (Kaunas	2021
903	C	Not relevant	34984210	Factors for severe outcomes following SARS-CoV-2 infection in people with c	Jung A, Orenti A,	ERJ Open Res	2021
904	C	Not relevant	34500569	Computational Prediction of Compound-Protein Interactions for Orphan Targ	Kanai C, Kawasa	Molecules	2021
905	C	Not relevant	33509858	Bardet-Biedl syndrome presenting with laryngeal web and bifid epiglottis	Kaur P, Chaudhr	BMJ Case Rep	2021
906	C	Not relevant	33479225	Sarcoma classification by DNA methylation profiling	Koelsche C, Schri	Nat Commun	2021
907	C	Not relevant	34338422	Exome survey of individuals affected by VATER/VACTERL with renal phenoty	Kolvenbach CM,	Am J Med Genet	2021
908	C	Not relevant	34503684	Medial Arterial Calcification: JACC State-of-the-Art Review	Lanzer P, Hanna	J Am Coll Cardiol	2021
909	C	Not relevant	33936945	Whale lice (Isocyamus deltobranchium & Isocyamus delphinii; Cyamidae) pre	Lehnert K, IJsseld	Int J Parasitol Par	2021
910	C	Not relevant	33247522	Artificial Intelligence Applied to the Rapid Identification of New Antimalarial	Lima MNN, Borb	ChemMedChem	2021
911	C	Not relevant	34563217	JAK inhibitors: a potential treatment for JDM in the context of the role of int	Ll Wilkinson MG,	Pediatr Rheumat	2021
912	C	Not relevant	33991680	Contrasting epidemiology and genetic variation of Plasmodium vivax infectin	Lo, E; Russo, G; P	INTERNATIONAL	2021
913	C	Not relevant		Hypertrophic cardiomyopathy and arrhythmias as phenotype spectrum of Em	Lynch, M.; Vadga	Journal of the Ba	2021
914	C	Not relevant	34225665	Performance of Web tools for predicting changes in protein stability caused	Marabotti A, Del	BMC Bioinformat	2021
915	C	Not relevant	33864365	A rare missense variant in the ATP2C2 gene is associated with language impa	Martinelli, A; Ric	HUMAN MOLECU	2021
916	C	Not relevant	33749946	Single-cell transcriptomics reveal temporal dynamics of critical regulators of	Mayère C, Neirij	FASEB J	2021
917	C	Not relevant	34122158	Autistic Adult Health and Professional Perceptions of It: Evidence From the A	Micai M, Ciaram	Front Psychiatry	2021
918	C	Not relevant	34187505	Cutaneous mucinosis of infancy: a rare case of joint involvement	Morreale C, Blei	Pediatr Rheumat	2021
919	C	Not relevant	34073904	Application of Nanotechnology for Sensitive Detection of Low-Abundance Sin	Mukhtar M, Sarg	Nanomaterials (B	2021
920	C	Not relevant		Novel R225C variant identified in the HGD gene in Jordanian patients with al	Mwafi, NR; Ali, D	AIMS MOLECULA	2021
921	C	Not relevant		Karyotypic analyses of accessions of kersting's groundnut.	Odo, I. C.; Akane	African Crop Scie	2021
922	C	Not relevant	34407214	Clotting factor concentrates for preventing bleeding and bleeding-related co	Olasupo OO, Low	Cochrane Databa	2021
923	C	Not relevant	34809655	Vasculitis in a patient with mevalonate kinase deficiency (MKD): a case repor	Omoyinmi E, Ro	Pediatr Rheumat	2021
924	C	Not relevant	34440765	Autophagy and Mitophagy-Related Pathways at the Crossroads of Genetic Pa	Pacheco Y, Valey	Cells	2021
925	C	Not relevant	34118938	Frequency of Fabry disease in a juvenile idiopathic arthritis cohort	Paim-Marques L,	Pediatr Rheumat	2021
926	C	Not relevant		Cystic fibrosis in Canada: A historical perspective	Petruzziello-Pelle	CANADIAN JOUR	2021
927	C	Not relevant		(Pre-)breeding of potatoes suitable for organic farming in the ECOBREED pro	Plich, J.; Tatarow	Plant breeding fo	2021

928	C	Not relevant	33762274	Late survival in Ellis-van Creveld syndrome with common single atrium	Prajapati K, Path	BMJ Case Rep	2021
929	C	Not relevant	34871162	The need for continuous quality assessment for providing optimal comprehe	Ptasinski A, Colel	Allergy Asthma P	2021
930	C	Not relevant	33937968	Dravet syndrome and Dravet syndrome-like phenotype: a systematic review	Rampazzo ACM,	Neurogenetics	2021
931	C	Not relevant	33741271	The natural history of neurocognition in MPS disorders: A review	Shapiro, EG; Eise	MOLECULAR GEN	2021
932	C	Not relevant	33957837	Clinical, immunological, and genetic features in 938 patients with autoimmu	Sharifinejad N, Z	Expert Rev Clin Im	2021
933	C	Not relevant	35130404	Infantile myofibromatosis: multiple firm nodules in a premature newborn	Szeto MD, Maym	Dermatol Online	2021
934	C	Not relevant	34837688	Pre-Implantation Genetic Testing for Monogenic Disorders (PGT-M) in A Fam	Tabatabaei Z, Ka	Cell J	2021
935	C	Not relevant		Allelotyping of S-RNase gene and promoter region of MdMYB10 gene in som	Vakili-Gartavol,	Journal of Horticu	2021
936	C	Not relevant	34193201	Patient with H syndrome, cardiogenic shock, multiorgan infiltration, and digit	Ventura-Espejo L	Pediatr Rheumat	2021
937	C	Not relevant	34479523	Pulmonary fibrosis in dyskeratosis congenita: a case report with a PRISMA-co	Wang P, Xu Z.	BMC Pulm Med	2021
938	C	Not relevant	33757531	Diagnosis and management of adenosine deaminase 2 deficiency children: th	Wang W, Zhang	Pediatr Rheumat	2021
939	C	Not relevant	34521435	Colchicine - an effective treatment for children with a clinical diagnosis of au	Welzel T, Wilder	Pediatr Rheumat	2021
940	C	Not relevant	34260399	Thousands of induced germline mutations affecting immune cells identified b	Xu D, Lyon S, Bu	Proc Natl Acad Sc	2021
941	C	Not relevant	33546616	Systematic review and subgroup analysis of the incidence of acute kidney inj	Xu Z, Tang Y, Hua	BMC Nephrol	2021
942	C	Not relevant	34209609	Selection and Validation of Reference Genes for Gene Expression Analysis in	Yan X, Zhang Y, X	Insects	2021
943	C	Not relevant	33462443	Detection of aberrant gene expression events in RNA sequencing data	Yépez VA, Merte	Nat Protoc	2021
944	C	Not relevant		Seroreactivity to Coxiella burnetii in an agricultural population and prevalenc	Yoo JeongRae; K	Pathogens	2021
945	C	Not relevant	34708592	Five novel globin gene mutations identified in five Chinese families by next-g	Zhang J, Xie M, P	Mol Genet Geno	2021
946	C	Not relevant	33407657	Type I interferonopathies with novel compound heterozygous TREX1 mutati	Zhang S, Song J,	Pediatr Rheumat	2021
947	C	Not relevant	34462954	Identification of novel compound heterozygous ITGB4 mutations in a Chines	Zhou X, Wang M	J Dermatol	2021
948	C	Not relevant	35762945	Improving candidate Biosynthetic Gene Clusters in fungi through reinforcem	Almeida H, Tsan	Bioinformatics	2022
949	C	Not relevant		Epididymo-orchitis caused by POM-1 metallo-β-lactamase-producing Pseudo	Alqurashi, M.; Al	Pathogens	2022
950	C	Not relevant	35533055	Lysozyme amyloidosis-a report on a large German cohort and the characteris	Anker, S; Hinder	AMYLOID-JOURN	2022
951	C	Not relevant	34355387	Psychosocial interventions and needs among individuals and families with Li-	Barnett M, Bree	Clin Genet	2022
952	C	Not relevant		Pancreatoblastoma in previously pancreatic pseudocysts in a 14-year-old fem	Bellynda, M.; Ma	Medical Journal o	2022
953	C	Not relevant	35751020	Whole genome sequencing analysis to evaluate the influence of T2DM on po	Bermudez-Herná	BMC Genomics	2022
954	C	Not relevant	35764379	Uncovering the burden of hidden ciliopathies in the 100 000 Genomes Projec	Best S, Yu J, Lord	J Med Genet	2022
955	C	Not relevant		Odontogenic cervicofacial necrotizing fasciitis: microbiological characterizati	Böttger, S.; Zech	Pathogens	2022
956	C	Not relevant	35898688	Xeroderma Pigmentosum: A Genetic Condition Skin Cancer Correlated-A Syst	Brambullo T, Col	Biomed Res Int	2022
957	C	Not relevant	35288775	[Transition to adult care for patients with epilepsy]	Brandl U.	Nervenarzt	2022
958	C	Not relevant	35410415	Precision treatment of Singleton Merten syndrome with ruxolitinib: a case re	Broser P, von Me	Pediatr Rheumat	2022
959	C	Not relevant	36075929	Germline-somatic JAK2 interactions are associated with clonal expansion in	Brown DW, Zhou	Nat Commun	2022

960	C	Not relevant	36327219	Exome-wide association study to identify rare variants influencing COVID-19	Butler-Laporte G	PLoS Genet	2022
961	C	Not relevant	35093554	Gut microbiome alterations in hereditary angioedema	Cao, Y; Kan, HX;	ANNALS OF ALLE	2022
962	C	Not relevant	35605311	Research Paper Safety of Onasemnogene Abeparvovec for Patients With Spi	Chand, DH; Mitc	PEDIATRIC NEUR	2022
963	C	Not relevant	36375482	Protection from previous natural infection compared with mRNA vaccination	Chemaitelly, H; A	LANCET MICROBE	2022
964	C	Not relevant	35024668	Prevalence of lysosomal storage disorders in Australia from 2009 to 2020	Chin, SJ; Fuller, M	LANCET REGIONA	2022
965	C	Not relevant		The impact of water and other fluids on pediatric nephrolithiasis.	Ciongradi, C. I.; F	Nutrients	2022
966	C	Not relevant	35305867	Genomic answers for children: Dynamic analyses of > 1000 pediatric rare dis	Cohen, ASA; Farr	GENETICS IN MED	2022
967	C	Not relevant	35343909	Safety of Triage Self-assessment Using a Symptom Assessment App for Walk-	Cotte F, Mueller	JMIR Mhealth Uh	2022
968	C	Not relevant	35304014	[French practical guidelines for the diagnosis and management of IPF - 2021	Cottin V, Bonnia	Rev Mal Respir	2022
969	C	Not relevant	35644166	Upadacitinib as induction and maintenance therapy for moderately to severe	Danese S, Verme	Lancet	2022
970	C	Not relevant	35368514	Rationale and design of the PROspective ATHletic Heart (Pro@Heart) study: l	De Bosscher R, D	BMJ Open Sport E	2022
971	C	Not relevant		1747P Impact of prior chemotherapy (Chemo) on pembrolizumab (Pembro) r	De Wit, R	Annals of oncolog	2022
972	C	Not relevant	35774508	Identification of Immune-Related Hub Genes in Thymoma: Defects in CD247	Deng LF.	Front Genet	2022
973	C	Not relevant	35578211	Rare co-occurrence of multiple sclerosis and Wilson's disease - case report	Despotov K, Kliv	BMC Neurol	2022
974	C	Not relevant	35120815	The tangled web of autoreactive B cells in malaria immunity and autoimmun	Dizon BLP, Pierce	Trends Parasitol	2022
975	C	Not relevant	35140190	Genotypes and phenotypes characterization of 17 Iranian patients with inher	Fallah A, Shams	Blood Coagul Fibr	2022
976	C	Not relevant	35988270	Reconstruction of post-burn hand contractures with Trapeze flap	Fattah JH.	Cell Mol Biol (Noi	2022
977	C	Not relevant	35053437	A Systematic Review and Meta-Analysis of Malignant Rhabdoid and Small Ce	Fuchs J, Murtha-	Cancers (Basel)	2022
978	C	Not relevant	35717242	Monogenic disorders as mimics of juvenile idiopathic arthritis	Furness L, Riley P	Pediatr Rheumat	2022
979	C	Not relevant	35340600	A bibliometric review of peripartum cardiomyopathy compared to other card	Grosser M, Lin H	Biophys Rev	2022
980	C	Not relevant		Effects of Agaricus blazei acidic polysaccharide on the aging of mice through	Guo, X; Ye, YJ; Li	ELECTRONIC JOU	2022
981	C	Not relevant	36529768	Mapping age- and sex-specific HIV prevalence in adults in sub-Saharan Africa	Haeuser E, Serfe	BMC Med	2022
982	C	Not relevant	36073806	Zebra: Static and Dynamic Genome Cover Thresholds with Overlapping Refer	Hakim D, Wandr	mSystems	2022
983	C	Not relevant	35780804	Risk of second brain tumour after radiotherapy for pituitary adenoma or cran	Hamblin R, Vard	Lancet Diabetes E	2022
984	C	Not relevant	36056432	Clinical phenotypes and genetic features of hereditary transthyretin amyloid	He X, Tian Z, Gua	Orphanet J Rare	2022
985	C	Not relevant		Clinical response to treatment with teriparatide in an adolescent with osteop	Homaei, A.; Cheg	International Jou	2022
986	C	Not relevant		Diarrhea triggered by breastfeeding: a novel variant causing congenital lacta	Hosnut, F. Ö.; Sa	Erciyes Medical J	2022
987	C	Not relevant	35753523	NUDT1 promotes the accumulation and longevity of CD103(+) T-RM cells in p	Huang, BY; Lyu, Z	JOURNAL OF HEP	2022
988	C	Not relevant	35657818	Prediction of various blood group systems using Korean whole-genome sequ	Hyun J, Oh S, Ho	PLoS One	2022
989	C	Not relevant	35906690	The efficacy and safety of allogeneic stem cell transplantation in Mevalonate	Jeyaratnam J, Fa	Pediatr Rheumat	2022
990	C	Not relevant	34988950	Study of the Demographic and Clinical Profile in a Neurocutaneous Rare Dise	Jouybari L, Foji S	Acta Neurol Taiw	2022
991	C	Not relevant	35656327	New Insights on the Regulatory Gene Network Disturbed in Central Areolar C	Kazmierczak de	Front Genet	2022

992	C	Not relevant		Multicenter surveillance of cystic fibrosis in Korean children.	Kim HyungYoung	Allergy, Asthma &	2022
993	C	Not relevant	35358455	Anetumab ravtansine versus vinorelbine in patients with relapsed, mesotheli	Kindler HL, Nove	Lancet Oncol	2022
994	C	Not relevant	35429480	Safety and efficacy of ganaxolone in patients with CDKL5 deficiency disorder:	Knight EMP, Ami	Lancet Neurol	2022
995	C	Not relevant	35154238	Genetic Testing for Rare Diseases: A Systematic Review of Ethical Aspects	Kruse J, Mueller	Front Genet	2022
996	C	Not relevant	35239007	Genotype-phenotype association of TARDBP mutations in Chinese patients w	Li J, Liu Q, Sun X,	J Neurol	2022
997	C	Not relevant	36060294	Characteristics and Challenges of Primary Adrenal Insufficiency in Africa: A R	Mofokeng TRP, B	Int J Endocrinol	2022
998	C	Not relevant	35218976	Genetic susceptibility to viral disease in humans	Mogensen, TH	CLINICAL MICROB	2022
999	C	Not relevant	35218979	Human genetics of SARS-CoV-2 infection and critical COVID-19	Mogensen, TH	CLINICAL MICROB	2022
1000	C	Not relevant	35254387	Multicenter Consensus Approach to Evaluation of Neonatal Hypotonia in the	Morton SU, Chris	JAMA Neurol	2022
1001	C	Not relevant		Protective Role of Pre-/ Post-biotics on Gut Inflammation, Dysbiosis, and Life	NCT05420805	https://clinicaltri	2022
1002	C	Not relevant		Intracranial calcification and seizure with down syndrome: a case report.	Nilshan Rai; Jine	JNMA, Journal of	2022
1003	C	Not relevant		The research of parasitic diseases of wild animals on the territory of the Fede	Omeragic, J.; Kap	Veterinaria (Saraj	2022
1004	C	Not relevant	35213607	Pediatric growth hormone treatment in Italy: A systematic review of epidem	Orso M, Polisten	PLoS One	2022
1005	C	Not relevant	35249861	What We Know About Penile Mondor's Disease	Özkan B, Coşkun	Sex Med Rev	2022
1006	C	Not relevant	35577511	Effect of sodium phenylbutyrate/taurursodiol on tracheostomy/ventilation-f	Paganoni S, Hen	J Neurol Neurosu	2022
1007	C	Not relevant	36153662	Juvenile mucopolysaccharidosis plus disease caused by a missense mutation	Pavlova EV, Lev	Hum Mutat	2022
1008	C	Not relevant	36240579	Behavioral phenotyping of young Scn1a haploinsufficient mice	Reiber, M; Milja	EPILEPSY & BEHA	2022
1009	C	Not relevant	35668118	MCL1 as putative target in pancreatoblastoma	Reissig TM, Uhri	Virchows Arch	2022
1010	C	Not relevant	36240581	Predicting clinical phenotypes of metachromatic leukodystrophy based on th	Santhanakumara	MOLECULAR GEN	2022
1011	C	Not relevant	34953404	The impact of COVID-19 on cancer care and oncology clinical research: an ex	Sessa C, Cortes J	ESMO Open	2022
1012	C	Not relevant		Distribution of serotypes and antibiotic resistance of avian pathogenic Esche	Sever, N. K.; Yapi	Kafkas Üniversite	2022
1013	C	Not relevant	35813426	Diagnosis of Brain Tumor Using Light Weight Deep Learning Model with Fine-	Shelatkar T, Urva	Comput Math Me	2022
1014	C	Not relevant	36061650	Computational Biology of BRCA2 in Male Breast Cancer, through Prediction o	Shinde SD, Satpu	ACS Omega	2022
1015	C	Not relevant	36467455	Individualised prediction of drug resistance and seizure recurrence after med	Stevelink R, Al-T	EClinicalMedicine	2022
1016	C	Not relevant	35892849	Activation of Oncogenic and Immune-Response Pathways Is Linked to Diseas	Sundqvist B, Kilp	Cancers (Basel)	2022
1017	C	Not relevant	35780807	Odevixibat treatment in progressive familial intrahepatic cholestasis: a rando	Thompson RJ, Ar	Lancet Gastroent	2022
1018	C	Not relevant		Alzheimer's disease: etiological and risk factors, pathophysiological mechanis	Trobec, T.; Žužek	Zdravniški Vestni	2022
1019	C	Not relevant	35579625	Discovering a new part of the phenotypic spectrum of Coffin-Siris syndrome	van der Sluijs PJ,	Genet Med	2022
1020	C	Not relevant	35151277	The research landscape of tuberous sclerosis complex-associated neuropsych	Vanclooster S, Bi	J Neurodev Disor	2022
1021	C	Not relevant		Tools for computational design and high-throughput screening of therapeuti	Vasina, Michal	Advanced Drug D	2022
1022	C	Not relevant		Increased Interest in Anatomical Donation As A Result of Positive Media Cov	Walker CM	FASEB journal	2022
1023	C	Not relevant	35123508	NLRP12-associated systemic autoinflammatory diseases in children	Wang HF.	Pediatr Rheumat	2022

1024	C	Not relevant	36338666	Clinical and genetic studies of 17 Han Chinese pedigrees and 31 sporadic pati	Wang Y, Wu Q, C	Mol Vis	2022
1025	C	Not relevant		Multiplex PCR identification of Aspergillus cristatus and Aspergillus chevalier	Wang Zhong; Jin	Foods	2022
1026	C	Not relevant	34991944	An observational, prospective, multicenter, natural history study of patients	Wijburg, FA; Aiac	MOLECULAR GEN	2022
1027	C	Not relevant		Characterization of gut microbiome in the mud snail Cipangopaludina cathay	Wu YangYang; C	Animals	2022
1028	C	Not relevant	36115379	Human infection of avian influenza A H3N8 virus and the viral origins: a descr	Yang, RG; Sun, H	LANCET MICROBE	2022
1029	C	Not relevant		Clinical and pathological diagnosis of hereditary gastrointestinal polyposis in	Yoneji, W.; Yoshi	Veterinary Scienc	2022
1030	C	Not relevant	34596301	Biallelic AOPEP Loss-of-Function Variants Cause Progressive Dystonia with Pr	Zech M, Kumar K	Mov Disord	2022
1031	C	Not relevant		Investigation of parasitic infection in crocodile lizards (Shinisaurus crocodilur	Zeng YongRu; Xi	Animals	2022
1032	C	Not relevant	35982159	Integrating de novo and inherited variants in 42,607 autism cases identifies	Zhou X, Feliciano	Nat Genet	2022
1033	C	Not relevant	37293316	A pragmatic approach to the diagnosis of inborn errors of metabolism in dev	Anetor, J. I.; Orim	African Journal of	2023
1034	C	Not relevant	37549695	European candidaemia is characterised by notable differential epidemiology	Arendrup MC	J Infect	2023
1035	C	Not relevant	36460017	Overall survival with first-line atezolizumab in combination with vemurafenib	Ascierto PA, Stro	Lancet Oncol	2023
1036	C	Not relevant	36864288	Recurrence mutation in RBBP8 gene causing non-syndromic autosomal reces	Batool T, Irshad	J Hum Genet	2023
1037	C	Not relevant	37211769	Current advancements in therapy for Niemann-Pick disease: progress and pit	Bremova-Ertl T,	Expert Opin Phar	2023
1038	C	Not relevant		Pancreatic cancer -impact of genetic and environmental factors.	Centner, H.; Lauš	Hrana u Zdravlju i	2023
1039	C	Not relevant	36904136	Investigating the dietary intake using the CyFFQ semi-quantitative food frequ	Christodoulou, C	Nutrients	2023
1040	C	Not relevant		Von Hippel-Lindau disease and agenesis of the corpus callosum: report of a n	Çolak, E.; Özkan,	Erciyes Medical J	2023
1041	C	Not relevant	37052967	Patients' and Members of the Public's Wishes Regarding Transparency in the	Cumyn, A; Mena	JOURNAL OF MED	2023
1042	C	Not relevant	36691078	Dry synovitis, a rare entity distinct from juvenile idiopathic arthritis	De Somer L, Bad	Pediatr Rheumat	2023
1043	C	Not relevant	36868990	ABCB5+mesenchymal stromal cells facilitate complete and durable wound cl	Dieter, K; Nieber	CYTOTHERAPY	2023
1044	C	Not relevant	37342181	Characterization of global research trends and prospects on platinum-resista	Duan Y	Front Oncol	2023
1045	C	Not relevant		A fatal case of familial hemophagocytic lymphohistiocytosis associated with	Dündar, M. A.; O	Erciyes Medical J	2023
1046	C	Not relevant	37513616	An insight into the exploration of antibiotic resistance genes in calorie restric	Fan XiuQin; Lu Y	Nutrients	2023
1047	C	Not relevant	36441344	Movement disorders in hereditary spastic paraplegia (HSP): a systematic revi	Fereshtehnejad	Neurol Sci	2023
1048	C	Not relevant	34216700	Awake airway endoscopy in mucopolysaccharidosis: a case report	Fernandes, S; Bo	BRAZILIAN JOURN	2023
1049	C	Not relevant	36598939	Iterative computational design and crystallographic screening identifies pote	Gahbauer S, Corr	Proc Natl Acad Sc	2023
1050	C	Not relevant	37436303	New challenges in health technology assessment (HTA): the case of Zolgensm	Guimarães, R.	Ciência & Sa	2023
1051	C	Not relevant	36826837	Diagnosis of TBC1D32-associated conditions: Expanding the phenotypic spec	Harris SC, Chong	Am J Med Genet	2023
1052	C	Not relevant	35346640	Frequency of alleles and genotypes associated with alpha-1 antitrypsin defici	Hernandez-Pere	PULMONOLOGY	2023
1053	C	Not relevant	37063926	Bioinformatics-integrated screening of systemic sclerosis-specific expressed	Jin J, Liu Y, Tang	Front Immunol	2023
1054	C	Not relevant	36403688	Bank Voles Show More Impulsivity in IntelliCage Learning Tasks than Wood M	Jorimann, M; Ma	NEUROSCIENCE	2023
1055	C	Not relevant	36787101	A 24-Week, Phase IIa, Randomized, Double-Blind, Placebo-Controlled Study o	Khanna D, Dento	Arthritis Rheuma	2023

1056	C	Not relevant	36752464	Heart Rate Variability Analysis May Identify Individuals With Williams-Beuren	Levin MD, Cathe	JACC Clin Electrop	2023
1057	C	Not relevant		Case of Seckel syndrome in a 9-month-old girl.	Martha Kelana, A	Open Access Mac	2023
1058	C	Not relevant	37628706	PSTPIP1-Associated Myeloid-Related Proteinemia Inflammatory (PAMI) Synd	Mejbri, M	Genes (Basel)	2023
1059	C	Not relevant		Cutaneous mastocytosis in childhood: an update from the literature.	Özdemir, Ö.; Sav	Erciyes Medical J	2023
1060	C	Not relevant	37444014	E-cadherin immunostaining in equine melanocytic tumors.	Pimenta, J.; Pires	Animals	2023
1061	C	Not relevant	36349687	Identifying causal serum protein-cardiometabolic trait relationships using wh	Png G, Gerlini R,	Hum Mol Genet	2023
1062	C	Not relevant		A Comprehensive Review on Technological Advances in Alternate Drug Disco	Pola, M	Current Trends in	2023
1063	C	Not relevant	37217202	Kitaviruses: A Window to Atypical Plant Viruses Causing Nonsystemic Disease	Ramos-González	Annu Rev Phytop	2023
1064	C	Not relevant	37273037	The future of rare autonomic disease research	Rand, CM	Clin Auton Res	2023
1065	C	Not relevant	36795469	Emergent dynamics of adult stem cell lineages from single nucleus and single	Raz AA, Vida GS,	Elife	2023
1066	C	Not relevant	37111473	Back from exile? First records of chewing lice Lutridia exilis; Ischnocera; Mall	Rohner, S.; Boyi,	Pathogens	2023
1067	C	Not relevant	37632635	Multiple endocrine neoplasia type 4 (MEN4): a thorough update on the lates	Ruggeri RM	Endocrine	2023
1068	C	Not relevant	36764972	Remote assessment of ADHD in children and adolescents: recommendations	Santosh, P	European Child &	2023
1069	C	Not relevant	37464858	Tuberous sclerosis complex in a 17-month-old: a case report.	Sarjan, K. C.; Boh	JNMA, Journal of	2023
1070	C	Not relevant	37516884	Total choline intake and working memory performance in adults with phenyl	Schoen MS	Orphanet J Rare	2023
1071	C	Not relevant	36774885	The efficacy of intranasal oxytocin in patients with Prader-Willi syndrome: A	Shalma NM, Alsh	Diabetes Metab S	2023
1072	C	Not relevant	37006978	Genetic Spectrum in F13A1 Detected by Next-Generation Sequencing Among	Sharma R, Jamw	Indian J Hematol	2023
1073	C	Not relevant	37440426	Exploring the rare variants associated with Type 2 Diabetes Mellitus in Indian	Shelake G	J Biomol Struct D	2023
1074	C	Not relevant	37508232	Meningococcal antibiotic resistance: molecular characterization of isolates fr	Spiliopoulou, I.;	Antibiotics	2023
1075	C	Not relevant	36902710	2-Year Change in Revised Hammersmith Scale Scores in a Large Cohort of Un	Stimpson G, Ram	J Clin Med	2023
1076	C	Not relevant	37287551	Familial Adult Myoclonic Epilepsy: Clinical and Genetic Approach to an Under	Uzun GA, Baykan	Noro Psikiyatr Ar	2023
1077	C	Not relevant	36796629	Characterization of Global Research Trends and Prospects on Moyamoya Dis	Wang C, Kong D,	World Neurosurg	2023
1078	C	Not relevant		A novel universal primer multiplex real-time PCR (UP-M-rtPCR) approach for	Wang WenJun;	Foods	2023
1079	C	Not relevant	37530275	A rare optineurin mutation in an Indian family with coexistence of JOAG and	Yadav M	Indian J Ophthalm	2023
1080	C	Not relevant	36599632	Alleviation of Hepatic Steatosis by 4-azidophlorizin via the Degradation of Ge	Ye J, Qi Y, Chen J	Adv Biol (Weinh)	2023
1081	C	Not relevant	36830556	Identification of personality-related candidate genes in Thoroughbred raceho	Yokomori, T.; Oh	Animals	2023
1082	C	Not relevant		[Identification of potential biomarkers of pigmented villonodular synovitis by	Yuan, L	Chinese Journal o	2023
1083	C	Not relevant	37422901	TBK1 variants in Chinese patients with amyotrophic lateral sclerosis: Genetic	Zhao B	Eur J Neurol	2023
1084	C	Not relevant	37171577	Genetic and Phenotypic Spectrum of Amyotrophic Lateral Sclerosis Patients	Zhao B, Jiang Q,	Mol Neurobiol	2023
1085	C	Not relevant	37610949	Enhancing understanding of SARS-CoV-2 infection among individuals with Do	Gomes da Silva,	Sao Paulo Med J	2023
