# Supplementary material for: Online education for rare genetic diseases: a systematic review
Source: Orphanet J Rare Dis. 2025 Dec 31;21:42. doi: 10.1186/s13023-025-03809-x (PMC12874925; doi:10.1186/s13023-025-03809-x)
Supplement: Supplementary file 2 — Additional file 2. [file 13023_2025_3809_MOESM2_ESM.pdf]

Supplementary Materials 2

Post-Review Results Spreadsheet

#	Grade	Category	PMID	Title	Authors	Journal	Year
1	A	video	38124115	An evaluation of an open access iPSC training cour	Schweikert A, Ken	Stem Cell Res Ther	2023
2	A	electronic document	37658574	Development and evaluation of an educational res	Blankenship K, Ch	J Genet Couns	2024
3	A	online course	38727543	A national postgraduate nurse practitioner and phy	Brady CJ, Looman	J Am Assoc Nurse Prac	2024
4	A	website	38296035	ERN BOND: The key European network leveraging d	Casareto L, Appel	Eur J Med Genet	2024
5	A	online course	39039494	Rare disease 101: an online resource teaching on c	Dunne TF, Jeffries	Orphanet J Rare Dis	2024
6	A	online conference/webinars	39989936	Harnessing genomics and translational research to	Nkya S, David A, A	Pan Afr Med J	2024
7	A	online conference/webinars	37969032	The 8th International RASopathies Symposium: Exp	Pierpont EI, Benne	Am J Med Genet A	2024
8	A	online conference/webinars	38306718	European Joint Programme on Rare Diseases works	Smeets H, Verbrug	Neuromuscul Disord	2024
9	A	web application/module	38488790	Recognition of Genetic Conditions After Learning W	Waikel RL, Othma	JAMA Netw Open	2024
10	B	Bioinformatics tool/resource	38344682	Genetic and clinical characterization of familial ren	Xu L, Zhao R, Zhao	Clin Kidney J	2023
11	B	Bioinformatics tool/resource	37637211	Cis-Cardio: A comprehensive analysis platform for	Song C, Zhang Y, H	Mol Ther Nucleic Acids	2023
12	B	Bioinformatics tool/resource	37697433	Assessing base-resolution DNA mechanics on the g	Jiang WJ, Hu C, La	Nucleic Acids Res	2023
13	B	Bioinformatics tool/resource	36993726	Developmental isoform diversity in the human neo	Patowary A, Zhang	bioRxiv	2023
14	B	Bioinformatics tool/resource	37599175	A life-threatening bleeding prediction model for imi	An ZY, Wu YJ, Hou	Sci Bull (Beijing)	2023
15	B	Bioinformatics tool/resource	38788190	TARO: tree-aggregated factor regression for microb	Mishra AK, Mahmu	Bioinformatics	2024
16	B	Bioinformatics tool/resource	39602828	FunlncModel: integrating multi-omic features from	Li YY, Qian FC, Zha	Brief Bioinform	2024
17	B	Bioinformatics tool/resource	38484704	Modular vector assembly enables rapid assessmen	McGee AV, Liu YV,	Cell Genom	2024
18	B	Bioinformatics tool/resource	38211594	SIGMA leverages protein structural information to p	Zhao H, Du H, Zha	Cell Rep Methods	2024
19	B	Bioinformatics tool/resource	38607411	VARista: a free web platform for streamlined whole	Hadar N, Dolgin V,	Hum Genet	2024
20	B	Bioinformatics tool/resource	38102483	Curation of causal interactions mediated by genes	Iannuccelli M, Vitr	Mol Psychiatry	2024
21	B	Bioinformatics tool/resource	37956336	KnockTF 2.0: a comprehensive gene expression pro	Feng C, Song C, So	Nucleic Acids Res	2024
22	B	Bioinformatics tool/resource	37889053	scGRN: a comprehensive single-cell gene regulato	Huang X, Song C, Z	Nucleic Acids Res	2024
23	B	Bioinformatics tool/resource	38682594	SynDesign: web-based prime editing guide RNA des	Park J, Yu G, Seo S	Nucleic Acids Res	2024
24	B	Bioinformatics tool/resource	38175747	Protocol for detecting rare and common genetic as	Yuan J, Li K, Peng H	STAR Protoc	2024
25	B	Bioinformatics tool/resource	39407093	Be-dataHIVE: a base editing database	Schneider L, Mina	BMC Bioinformatics	2024
26	B	Bioinformatics tool/resource	38187171	FDrisk: development of a validated risk assessmen	Lobel CJ, Laney DA	J Rare Dis (Berlin)	2024
27	B	Bioinformatics tool/resource	39149406	VUStruct: a compute pipeline for high throughput a	Moth CW, Sheeha	bioRxiv	2024
28	B	Bioinformatics tool/resource	39176563	An Integrated Pipeline for Phenotypic Characterizat	Chen X, Wang J, Fa	Stud Health Technol In	2024
29	B	Bioinformatics tool/resource	39499532	CACHE Challenge #1: Targeting the WDR Domain o	Li F, Ackloo S, Arro	J Chem Inf Model	2024
30	B	Bioinformatics tool/resource	39096608	i-Dent: A virtual assistant to diagnose rare genetic d	Kadi H, Kawczynsk	Comput Biol Med	2024

31	B	Bioinformatics tool/resource	37865290	A New Set of in Silico Tools to Support the Interpret	Porras LM, Padilla	J Mol Diagn	2024
32	B	Bioinformatics tool/resource	39360376	Risk prediction modelling in idiopathic inflammato	Zhang W, Huang G	Clin Exp Rheumatol	2025
33	B	Bioinformatics tool/resource	39950976	Machine learning-based survival prediction tool for	Saygili ES, Elhassa	J Clin Endocrinol Meta	2025
34	B	Bioinformatics tool/resource	39700857	UniVar: A variant interpretation platform enhancing	Au-Yeung CCY, Ch	Comput Biol Med	2025
35	B	Clinical intervention	39033111	Early treatment for children with mental health pro	Serrano M, Elias M	Trials	2024
36	B	Clinical intervention	39626227	Patients' and Clinicians' Experiences Using a Real-T	McCann L, Lewis L	J Med Internet Res	2024
37	B	Clinical intervention	38318960	The impact of a virtual mind-body program on symp	McDermott K, Bak	Am J Med Genet A	2024
38	B	Clinical intervention	38950130	Development and evaluation of a personalised psy	Sarkany R, Walbur	Development and eval	2024
39	B	Clinical intervention	39548598	Effects of internet-based health education on patie	Zhao L, Liu Y, Li J, I	Orphanet J Rare Dis	2024
40	B	Clinical intervention	39103734	Virtual reality tolerability, sense of presence and us	Migliore S, Casella	Neurol Sci	2025
41	B	Database/registry/network	37414863	The Korean Genetic Diagnosis Program for Rare Dis	Kim MJ, Kim B, Lee	Eur J Hum Genet	2023
42	B	Database/registry/network	37516995	Neuromuscular disease genetics in under-represen	Wilson LA, Macken	Brain	2023
43	B	Database/registry/network	38519604	Spanish registry of hemoglobinopathies and rare an	Bardón-Cancho EJ	Ann Hematol	2024
44	B	Database/registry/network	37898381	The Global ALPL gene variant classification project	Farman MR, Rehde	Bone	2024
45	B	Database/registry/network	39028753	PPCRKB: a risk factor knowledge base of postopera	Duan J, Li P, Shao	Database (Oxford)	2024
46	B	Database/registry/network	38965703	SpadaHC: a database to improve the classification	Moreno-Cabrera JI	Database (Oxford)	2024
47	B	Database/registry/network	38262306	The side effect registry immuno-oncology (SERIO) -	Ertl C, Ruf T, Mentz	Eur J Cancer	2024
48	B	Database/registry/network	38629401	Lethal phenotypes in Mendelian disorders	Cacheiro P, Lawso	Genet Med	2024
49	B	Database/registry/network	38061948	The myotubular and centronuclear myopathy patie	Bullivant J, Sen A,	Neuromuscul Disord	2024
50	B	Database/registry/network	39380901	SLC6A1 patient & organization perspective: foundir	Tiller J, DeLeeuw N	Ther Adv Rare Dis	2024
51	B	Database/registry/network	38834317	European Autism GEnomics Registry (EAGER): prot	Bloomfield M, Lau	BMJ Open	2024
52	B	Database/registry/network	38862132	Leveraging Patient Engagement Through Collabora	Evans D, Rothschi	Am Soc Clin Oncol Edu	2024
53	B	Database/registry/network	39725440	Paediatric Personalized Research Network Switzerl	Mozun R, Belle FN	BMJ Open	2024
54	B	Database/registry/network	39176652	Privacy-Preserving Linkage of Distributed Pseudony	Hayn D, Sandner E	Stud Health Technol In	2024
55	B	Database/registry/network	37703843	Development of a Minimum Dataset for the Monito	Chen SC, Bryce J,	Horm Res Paediatr	2024
56	B	Database/registry/network	39544699	Overcoming genetic neuromuscular diagnostic pitf	Frezatti RSS, Toma	Brain Commun	2024
57	B	Database/registry/network	39526399	SV4GD: a comprehensive structural variation datab	Shi L, Zhang S, Li Y	Nucleic Acids Res	2025
58	B	Database/registry/network	39885579	KaRhab: an international online registry for cardiac	Herrmann VM, Are	Orphanet J Rare Dis	2025
59	B	Delphi consensus/guidelines	37669844	Consensus recommendations on holistic care in he	Obici L, Callaghan	BMJ Open	2023
60	B	Delphi consensus/guidelines	38152089	Guideline for the management of myasthenic synd	Wiendl H, Abicht A	Ther Adv Neurol Disord	2023
61	B	Delphi consensus/guidelines	37658177	Peripheral Nervous, Hepatic, and Gastrointestinal I	Mauermann ML, C	Adv Ther	2023

62	B	Delphi consensus/guidelines	38670233	Definition, acronyms, nomenclature, and classifica	Reshef A, Buttgere	J Allergy Clin Immunol	2024
63	B	Delphi consensus/guidelines	39393368	Data-driven, harmonised classification system for	Komrokji RS, Lanir	Lancet Haematol	2024
64	B	Delphi consensus/guidelines	39024860	Monitoring and integrated care coordination of pati	Guffon N, Burton B	Mol Genet Metab	2024
65	B	Delphi consensus/guidelines	38347616	Recommendations for optimal interdisciplinary ma	Graessner H, Rein	Orphanet J Rare Dis	2024
66	B	Delphi consensus/guidelines	39185098	A General Neurologist's Practical Diagnostic Algorit	Bruno MK, Dhall R	Neurol Clin Pract	2024
67	B	Delphi consensus/guidelines	38843911	ERS statement on transition of care in childhood in	Pohunek P, Manal	Eur Respir J	2024
68	B	Delphi consensus/guidelines	39723120	Development of the Dutch translational knowledge	Hieltjes IJ, van der	JIMD Rep	2024
69	B	Delphi consensus/guidelines	39907276	Management of genetically determined kidney ston	Miano R, Gambaro	Minerva Urol Nephrol	2025
70	B	Knowledge assessment	38984594	Navigating Dravet syndrome in Spain: A cross-secti	Solaz S, Cardenal-	Epilepsia Open	2024
71	B	Knowledge assessment	38882666	Knowledge and thresholds for palliative care and su	Juan DWK, Ng IAT,	Front Med (Lausanne)	2024
72	B	Knowledge assessment	39736694	Impact of gender on self-assessment accuracy am	Bodard S, Bouzid D	BMC Med Educ	2024
73	B	Knowledge assessment	38105149	Identification and management of gastrointestinal	Cappello M, Barba	Dig Liver Dis	2024
74	B	Matchmaking	37812136	Facilitating the Molecular Diagnosis of Rare Geneti	Hsieh TC, Lesman	Curr Protoc	2023
75	B	Matchmaking	38477981	Validation of 3 Computer-Aided Facial Phenotyping	Reiter AMV, Pante	J Med Internet Res	2024
76	B	Non-English	37582888	[Diagnostics and management of patients with inhe	Künzel SH, Mahrer	Ophthalmologie	2023
77	B	Non-English	38742380	[Italian Cystic Fibrosis Registry (ICFR). Report 2021	Campagna G, Ama	Epidemiol Prev	2024
78	B	Non-English	38109904	[Kids Lung Registry and Child-EU Project - Progress	Griese M, Gold A, C	Klin Padiatr	2024
79	B	Resource list	37926987	An Expert Evaluation of Oncology Website Resourc	Banayat AC, Chall	J Pediatr Hematol Onc	2023
80	B	Resource list	39976996	Adeno-Associated Virus Gene Therapy Developmen	Lomash RM, Dehd	Hum Gene Ther	2025
81	B	Social media/web search	37707878	Patients With Rare Diseases and the Power of Onlin	Ashtari S, Taylor A	JMIR Form Res	2023
82	B	Social media/web search	38696211	My Digital Refuge	Wexler A.	JAMA	2024
83	B	Social media/web search	38224480	The Use of Social Media to Express and Manage Me	Pearce E, Raj H, Er	JMIR Infodemiology	2024
84	B	Social media/web search	39680438	"Crying in the Wilderness"-The Use of Web-Based S	Pearce EE, Majid A	JMIR Form Res	2024
85	B	Survey/interview	37752446	Informed choice and routinization of the second-tri	Lust EER, Bronsge	BMC Pregnancy Childb	2023
86	B	Survey/interview	37873848	Prospects for Expansion of Universal Newborn Scre	Iskrov G, Angelova	Int J Neonatal Screen	2023
87	B	Survey/interview	37632685	Genetic services survey-experience of people with	Ward AJ, Lambert	J Community Genet	2023
88	B	Survey/interview	37805563	Challenges in the care of individuals with severe pr	Backeljauw PF, An	Orphanet J Rare Dis	2023
89	B	Survey/interview	38661678	European pathways of care in Alpha-1 Antitrypsin d	Chorostowska-Wy	Respir Med	2023
90	B	Survey/interview	38976125	Impacts of X-linked Retinitis Pigmentosa and Patie	Pungor K, Lee J, De	Adv Ther	2024
91	B	Survey/interview	39317512	Perceptions of genomic newborn screening: a cros	Seed L, Scott A, Pi	BMJ Open	2024
92	B	Survey/interview	38035538	Non-pharmacological therapeutic needs in people	Ballesteros-Sayas	Epilepsy Behav	2024

93	B	Survey/interview	38349347	Insights into adherence to medication and lifestyle	Peltenburg PJ, van	Europace	2024
94	B	Survey/interview	39234078	Long-term distress throughout one's life: health-rel	Liang W, Cao S, Su	Front Public Health	2024
95	B	Survey/interview	39669611	ARID1B-related disorder in 87 adults: Natural histo	van der Sluijs PJ, G	Genet Med Open	2024
96	B	Survey/interview	38711219	'High hopes for treatment': Australian stakeholder p	Nguyen CQ, Kariya	Health Expect	2024
97	B	Survey/interview	37980193	PERSUADE Survey-PERioperative AnestheSia and In	Kummerow M, von	J Cardiothorac Vasc An	2024
98	B	Survey/interview	38386321	National Rapid Genome Sequencing in Neonatal In	Marom D, Mory A,	JAMA Netw Open	2024
99	B	Survey/interview	38323148	Walk a mile in my shoes: perspectives towards sha	Leigh S, Baines R,	Mhealth	2024
100	B	Survey/interview	38355752	Eliciting parental preferences and values for the ret	Goranitis I, Meng Y	NPJ Genom Med	2024
101	B	Survey/interview	39138481	Burden of illness in Rett syndrome: initial evaluatio	Kaufmann WE, Pe	Orphanet J Rare Dis	2024
102	B	Survey/interview	39252123	Preferences for coordinated care for rare diseases:	Morris S, Walton H	Orphanet J Rare Dis	2024
103	B	Survey/interview	38326898	Inventory of current practices regarding hematopoi	Schoenmakers DH	Orphanet J Rare Dis	2024
104	B	Survey/interview	38245779	Long-term monitoring of fatty acid oxidation defect	Schwantje M, Grün	Orphanet J Rare Dis	2024
105	B	Survey/interview	39707431	Review of a specialist Rett syndrome clinic from 20	Sloper E, Hunt M, (	Orphanet J Rare Dis	2024
106	B	Survey/interview	39614274	Shifting focus from ideality to reality: a qualitative s	van Lonkhuizen PJ	Orphanet J Rare Dis	2024
107	B	Survey/interview	37261843	A prospective survey on therapeutic inertia in psori	Lioté F, Constantin	Rheumatology (Oxford	2024
108	B	Survey/interview	38165635	Empowerment of genetic information by women at-	Hoefel AML, Wesc	J Community Genet	2024
109	B	Survey/interview	38403596	The burden of disease in metachromatic leukodyst	Thomas S, Morriso	Orphanet J Rare Dis	2024
110	B	Survey/interview	38441167	'Something that helped the whole picture': Experien	McInnes-Dean H,	Prenat Diagn	2024
111	B	Survey/interview	38643010	Patient preferences in genetic newborn screening f	Martin S, Angolini	BMJ Open	2024
112	B	Survey/interview	39354183	Defining the complex needs of families with rare dis	Wilsnack C, Rising	Eur J Hum Genet	2024
113	B	Survey/interview	40009279	Psychological supports for people living with a rare	Nerney D, O'Malle	Ir J Med Sci	2025
114	B	Survey/interview	38348940	Health-related quality of life and fear of progression	Kiermeier S, Schot	J Genet Couns	2025
115	B	Survey/interview	40025610	Diagnosis of hereditary transthyretin amyloidosis in	Péréon Y, Adams D	Orphanet J Rare Dis	2025
116	B	Survey/interview	38741243	Exploring the role of digital tools in rare disease ma	Chang A, Huang SI	J Genet Couns	2025
117	B	Telemedicine	37803436	Possible Concomitant Aggressive NK Cell Leukemia	Dayton VJ, Hoang T	Diagn Pathol	2023
118	B	Telemedicine	38168160	Reducing Time to Diagnosis of Rare Genetic Diseas	Vuocolo B, Sierra R	Res Sq	2023
119	B	Telemedicine	38946606	The Growing Role of Telerehabilitation and Teleass	Lavorgna L, Maida	Telemed J E Health	2024
120	B	Telemedicine	38231401	The Inborn Errors of Immunity-Virtual Consultation	Coppola E, Sgrulle	J Clin Immunol	2024
121	B	Telemedicine	38320840	Implementation of rapid genomic sequencing in sa	D'Gama AM, Hills S	BMJ Open	2024
122	B	Telemedicine	39251895	Project GIVE: using a virtual genetics service platfo	Vuocolo B, Sierra R	J Neurodev Disord	2024
123	B	Telemedicine	38190817	Expert Pathology for Gestational Trophoblastic Dise	Kaur B, Nadal A, B	Gynecol Obstet Invest	2024

124	B	Telemedicine	39629753	Telehealth Is Effective in the Evaluation of Individua	Tan QK, McConkie	Am J Med Genet A	2025
125	C	Not relevant	24260777	Biotin-Thiamine-Responsive Basal Ganglia Disease	Tabarki B, Al-Hash	GeneReviews(®)	1993
126	C	Not relevant	29989768	Lipodystrophy Syndromes: Presentation and Treatr	Akinci B, Gular MC	Endotext	2000
127	C	Not relevant	25905162	Adrenocortical Carcinoma	Angelousi A, Kassi	Endotext	2000
128	C	Not relevant	26844335	Pediatric Implications of Normal Insulin-GH-IGF Ax	Bang P.	Endotext	2000
129	C	Not relevant	25905184	Familial Isolated Pituitary Adenoma	Carreira AL, Korbo	Endotext	2000
130	C	Not relevant	25905384	Radiology of the Pituitary	Evanson J.	Endotext	2000
131	C	Not relevant	25905292	Genetic Obesity Syndromes	Farooqi IS.	Endotext	2000
132	C	Not relevant	25905391	Ambiguous Genitalia in the Newborn	Flück CE, Güran T.	Endotext	2000
133	C	Not relevant	25905168	Primary Generalized Glucocorticoid Resistance Syr	Kino T, Nicolaides	Endotext	2000
134	C	Not relevant	27809433	Familial Hypercholesterolemia	Levenson AE, de F	Endotext	2000
135	C	Not relevant	30321013	Infections Of The Hypothalamic-Pituitary Region	Pekic S, Miljic D, P	Endotext	2000
136	C	Not relevant	34878751	Monogenic Disorders Altering HDL Levels	Shapiro MD, Feing	Endotext	2000
137	C	Not relevant	26561704	Monogenic Disorders Causing Hypobetalipoprotein	Shapiro MD, Feing	Endotext	2000
138	C	Not relevant	25905375	Autoimmune Polyglandular Syndromes	Sperling MA, Ange	Endotext	2000
139	C	Not relevant	26561703	Hypertriglyceridemia: Pathophysiology, Role of Ger	Subramanian S.	Endotext	2000
140	C	Not relevant	38536945	Cushing Syndrome/Disease in Children and Adoles	Tatsi C.	Endotext	2000
141	C	Not relevant	25905179	Adrenal Insufficiency Due to X-Linked Adrenoleuko	Vlachou S, Kanaki	Endotext	2000
142	C	Not relevant	26844336	Familial Hypercholesterolemia: Genes and Beyond	Warden BA, Fazio	Endotext	2000
143	C	Not relevant	37929088	Transcript-Level In Silico Analysis of Alzheimer's Di	Azmi MB, Ahmed A	ACS Omega	2023
144	C	Not relevant	37961518	Modular vector assembly enables rapid assessmer	McGee AV, Liu YV,	bioRxiv	2023
145	C	Not relevant	37974187	Perampanel effectiveness in treating ROGDI-relate	Meng L, Huang D,	BMC Med Genomics	2023
146	C	Not relevant	37620677	Bibliometric analysis of the global publication activ	Cheng L, Liu Y, Ma	Clin Rheumatol	2023
147	C	Not relevant	37842447	Multicentric Osteolysis Nodulosis Arthropathy Synd	Imam SK, Alnaqeb	Cureus	2023
148	C	Not relevant	38229793	Marfanoid to Mortality: A Case Report on Sudden C	Toshniwal S, Chat	Cureus	2023
149	C	Not relevant	38478639	Two congenital cases of pigmented epithelioid mel	Zaaroura H, Cyren	Dermatol Online J	2023
150	C	Not relevant	37745025	Clinical phenotypes and outcomes in children with	Sperotto F, Gutiér	EClinicalMedicine	2023
151	C	Not relevant	37632635	Multiple endocrine neoplasia type 4 (MEN4): a thor	Ruggeri RM, Benev	Endocrine	2023
152	C	Not relevant	38116790	Barakat syndrome diagnosed decades after initial p	Spennato U, Siegw	Endocrinol Diabetes M	2023
153	C	Not relevant	38053725	Identification of key genes and pathways in adreno	Yin M, Wang Y, Re	Front Endocrinol (Laus	2023
154	C	Not relevant	38003043	Statistical Dissection of the Genetic Determinants	Lazareva TE, Barbi	Genes (Basel)	2023

155	C	Not relevant	37761896	Outcome Measures and Biomarkers for Clinical Tri	Siow SF, Yeow D, I	Genes (Basel)	2023
156	C	Not relevant	37549695	European candidaemia is characterised by notable	Arendrup MC, Arik	J Infect	2023
157	C	Not relevant	37739572	Safety and efficacy of tamoxifen in boys with Duche	Henzi BC, Schmid	Lancet Neurol	2023
158	C	Not relevant	37828538	Tocilizumab reduces the unmanageable inflammat	Wang W, Wang W,	Pediatr Rheumatol On	2023
159	C	Not relevant	37848905	Efficacy and safety of thalidomide in children with r	Zhang C, Yu Z, Ga	Pediatr Rheumatol On	2023
160	C	Not relevant	38130371	Isolated renal and urinary tract aspergillosis: a syst	Bongomin F, Morg	Ther Adv Urol	2023
161	C	Not relevant	38173711	ScreenPlus: A comprehensive, multi-disorder newl	Kelly NR, Orsini JJ,	Mol Genet Metab Rep	2023
162	C	Not relevant	39091406	Identification and characterization of multiple fung	Pramesh D, Prasa	3 Biotech	2024
163	C	Not relevant	39393841	Pituitary gland duplication syndrome - An internatio	Löbel U, Catala M,	AJNR Am J Neuroradio	2024
164	C	Not relevant	38951460	Human Inborn Errors of Immunity in Pyoderma Gan	Oprea Y, Antohi D	Am J Clin Dermatol	2024
165	C	Not relevant	39288765	The BabySeq Project: A clinical trial of genome seq	Smith HS, Zettler I	Am J Hum Genet	2024
166	C	Not relevant	38979718	The impact of impaired intrauterine growth on male	Meng F, Yao M, Li S	Andrology	2024
167	C	Not relevant	38556905	Evaluating mFARS in pediatric Friedreich's ataxia: I	Rummey C, Perlm	Ann Clin Transl Neurol	2024
168	C	Not relevant	39492681	Characterization of Genetic Landscape and Novel I	Topping J, Chang L	Arthritis Rheumatol	2024
169	C	Not relevant	39250567	Bezafibrate as treatment in males for Barth syndro	Pieles G, Steward	Bezafibrate as treatme	2024
170	C	Not relevant	39767580	Studying Rare Movement Disorders: From Whole-E	Marsili L, Duque K	Biomedicines	2024
171	C	Not relevant	39095810	Optimal treatment strategies for hepatoid adenoca	Deng H, Wang L, L	BMC Cancer	2024
172	C	Not relevant	39334246	Post-marketing surveillance framework of cell and	Cai Y, Sui L, Wang	BMC Med	2024
173	C	Not relevant	39215902	Double-hit primary central nervous system lympho	Onodera K, Shirah	Brain Tumor Pathol	2024
174	C	Not relevant	39097527	Treatment of Hailey-Hailey disease with biologics a	Liu W, Xue X, Li S.	Clin Exp Dermatol	2024
175	C	Not relevant	38182800	Global trends in research of melanoma differentiat	Yuan X, Shi J, Peng	Clin Rheumatol	2024
176	C	Not relevant	39331535	Advent of oral medications for the treatment of her	Valerieva A, Cabal	Clin Transl Allergy	2024
177	C	Not relevant	39569253	Psychosis in Laurence-Moon Syndrome: A Case Re	Al Fareh N, Abbas	Cureus	2024
178	C	Not relevant	39583597	Drug-Induced Type 1 Brugada Pattern: A Case Repo	Al-Anee O, Theeb I	Cureus	2024
179	C	Not relevant	39655114	An Unusual Presentation of Urinary Retention in a Y	Desouky O, Chkir I	Cureus	2024
180	C	Not relevant	39835031	Macrotrabecular-Massive Hepatocellular Carcinom	Eftimie Spitz R, Ma	Cureus	2024
181	C	Not relevant	39835009	Complex Genetic Framework in Familial Amyotropl	Frolov A, D'sa E, H	Cureus	2024
182	C	Not relevant	39564070	The Iron Enigma: Expounding Iron Deficiency in a P	Idrees Z, Khan H, A	Cureus	2024
183	C	Not relevant	39717303	Two Cases of Menkes Disease With Similar Intracra	Katase S, Tsuchiya	Cureus	2024
184	C	Not relevant	39650963	Lysosomal Acid Lipase Deficiency: A Report of Two	Nedelcu C, Dijmar	Cureus	2024
185	C	Not relevant	39569257	Segmental Darier's Disease Treated With Cryothera	Nguyen N, Liu K, S	Cureus	2024

186	C	Not relevant	39677172	Navigating Pompe Disease Assessment: A Compre	Nunes Campos L,	Cureus	2024
187	C	Not relevant	39840160	A Rare Case of Bartter Syndrome Type 3 Presenting	Pradeep U, Achary	Cureus	2024
188	C	Not relevant	39664132	Aplastic Anemia: Demographic and Clinical Charac	Rodríguez-Sevilla	Cureus	2024
189	C	Not relevant	39588395	Retinitis Pigmentosa Associated With EYS Gene Mu	Ruiz-Justiz AJ, Mol	Cureus	2024
190	C	Not relevant	38959927	Drug-Induced dermatomyositis following COVID-19	Herron E, Powell D	Dermatol Online J	2024
191	C	Not relevant	39154774	Preclinical alternative drug discovery programs for	Sebastiano MR, Ha	Drug Discov Today	2024
192	C	Not relevant	39020240	17α Hydroxylase/17,20 lyase deficiency: clinical fe	Siklar Z, Camtosur	Endocrine	2024
193	C	Not relevant	37726640	Clinical and genetic characteristics of CEL-MODY (	Sun S, Gong S, Li N	Endocrine	2024
194	C	Not relevant	38841617	Unraveling progression subtypes in people with Hu	Raschka T, Li Z, Ga	EPMA J	2024
195	C	Not relevant	38965372	Burden re-analysis of neurodevelopmental disorde	Smal N, Majdoub F	Eur J Hum Genet	2024
196	C	Not relevant	38566815	Genomic analysis in Chilean patients with suspect	Brito F, Lagos C, C	Front Genet	2024
197	C	Not relevant	39764439	Case Report and literature review: Delayed diagnos	Ouyang L, Yang F,	Front Genet	2024
198	C	Not relevant	38389573	Mitochondrial-related hub genes in dermatomyosit	Wang S, Tang Y, C	Front Genet	2024
199	C	Not relevant	38870335	Gene therapy for choroideremia using an adeno-as	Cehajic-Kapetano	Gene therapy for chor	2024
200	C	Not relevant	39202334	Auditory and Language Abilities in Children with Ta	Caragli V, Genove	Genes (Basel)	2024
201	C	Not relevant	38179805	Malignant carotid body tumors: What we know, wha	Piazza C, Lancini D	Head Neck	2024
202	C	Not relevant	38896399	Comparative policy analysis of national rare diseas	Ng QX, Ong C, Cha	Health Econ Rev	2024
203	C	Not relevant	39011238	Prothrombin G20210A Mutation is Rare but not Abs	Satyarthi P, Ray D,	Indian J Hematol Blood	2024
204	C	Not relevant	38827541	Bartonella quintana pulmonary native valve endoca	Aranda-Domene R	Indian J Thorac Cardio	2024
205	C	Not relevant	39416336	Patient with Fabry disease undergoing cardiac surg	Vuckovic J, Stojsic	Indian J Thorac Cardio	2024
206	C	Not relevant	39504391	Infliximab versus alpha interferon in the treatment	Moots RJ, Fortune	Infliximab versus alpha	2024
207	C	Not relevant	39201272	Normal Values for the fT3/fT4 Ratio: Centile Charts	Wilpert NM, Tham	Int J Mol Sci	2024
208	C	Not relevant	38156787	A commentary on 'Patient-derived gene and protein	Suzuki T.	J Biochem	2024
209	C	Not relevant	37440426	Exploring the rare variants associated with Type 2 D	Shelake G, Baviska	J Biomol Struct Dyn	2024
210	C	Not relevant	38398243	Androgen Insensitivity Syndrome with Bilateral Gon	Karseladze AI, Asa	J Clin Med	2024
211	C	Not relevant	38398338	Analysis of Anakinra Therapy for the Deficiency of I	Pillai K, Pillai J, Lin	J Clin Med	2024
212	C	Not relevant	39191560	Impact of COVID-19 infection on lung function and	Semenchuk J, Nait	J Cyst Fibros	2024
213	C	Not relevant	39449562	A survey to analyze the need of genetic counseling	Shaikh AA, Imran M	J Genet Couns	2024
214	C	Not relevant	38508706	A comparative medical genomics approach may fa	Haque B, Guirguis	J Med Genet	2024
215	C	Not relevant	38427497	Disease Trajectories in the Revised Hammersmith S	Wolfe A, Stimpson	J Neuromuscul Dis	2024
216	C	Not relevant	38464804	Left Main Stem Compression by Intrapericardial Pa	Wang X, Gondal M	JACC Case Rep	2024

217	C	Not relevant	39446377	Anti-Programmed Death Ligand 1 Plus Targeted The	Cabanillas ME, Da	JAMA Oncol	2024
218	C	Not relevant	39304265	Global, regional, and national burden of stroke and	GBD 2021 Stroke I	Lancet Neurol	2024
219	C	Not relevant	38276209	High Prevalence of Novel Sequence Types in Strept	Mokaddas E, Asad	Microorganisms	2024
220	C	Not relevant	38996836	Advances in the management of parathyroid carcin	Kubal M, Lech M, L	Mol Cell Endocrinol	2024
221	C	Not relevant	37864541	A collaborative backbone resource for comparative	Saclier N, Duchem	Mol Ecol Resour	2024
222	C	Not relevant	38555683	Diagnosis of alpha-Mannosidosis: Practical approa	Santoro L, Cefalo (	Mol Genet Metab	2024
223	C	Not relevant	38544910	Towards genomic-Newborn Screening: Technical fe	Mauri A, Berardo C	Mol Genet Metab Rep	2024
224	C	Not relevant	38357258	A Deeper Insight into COL4A3, COL4A4, and COL4A	Yavas C, Ozgentur	Mol Syndromol	2024
225	C	Not relevant	38760335	Evaluating the utility of multi-gene, multi-disease p	Liang JW, Christen	NPJ Genom Med	2024
226	C	Not relevant	39271120	Accelerated discovery and miniaturization of novel	Deng J, Li X, Yu H,	Nucleic Acids Res	2024
227	C	Not relevant	38566363	Root resorptions induced by genetic disorders: A sy	Dupre N, Riou MC,	Oral Dis	2024
228	C	Not relevant	38360662	Psychotic illness in people with Prader-Willi syndro	Aman LCS, Lester	Orphanet J Rare Dis	2024
229	C	Not relevant	39673054	Retrospective longitudinal study on the long-term ir	Braun S, Laemmer	Orphanet J Rare Dis	2024
230	C	Not relevant	38961493	Screening primary carnitine deficiency in 10 millior	Zhou J, Li G, Zeng Y	Orphanet J Rare Dis	2024
231	C	Not relevant	39416614	Prevalence-pattern of congenital and hereditary an	Azmatullah, Khan	Pak J Med Sci	2024
232	C	Not relevant	38395889	Juvenile eosinophilic fasciitis: a single center case	Stubbs LA, Ogunbe	Pediatr Rheumatol On	2024
233	C	Not relevant	38178067	A rare manifestation of STING-associated vasculop	Weidler S, Koss S,	Pediatr Rheumatol On	2024
234	C	Not relevant	38625690	First Report of Globisporangium (Pythium) mastopl	Zajc J, Kovačec E,	Plant Dis	2024
235	C	Not relevant	39148509	Contemporary Management of the Upper Limb in A	Khabyeh-Hasbani	Plast Reconstr Surg Gl	2024
236	C	Not relevant	39398478	Exploring the wild almond, Prunus arabica (Olivier)	Brukental H, Doro	Tree Genet Genomes	2024
237	C	Not relevant	38733460	Current understanding of ELF4 deficiency: a novel i	Du HQ, Zhao XD.	World J Pediatr	2024
238	C	Not relevant	38848795	Advances and opportunities in process analytical te	Sripada SA, Hosse	Biotechnol Adv	2024
239	C	Not relevant	39348199	POLR3B is associated with a developmental and ep	Symonds JD, Park	Epilepsia	2024
240	C	Not relevant	39578163	Amelogenesis imperfecta: Analysis of the genetic b	Klein P, Hoppe JS,	J Prosthet Dent	2024
241	C	Not relevant	39272071	Artificial intelligence empowering rare diseases: a I	Ou P, Wen R, Shi L	Orphanet J Rare Dis	2024
242	C	Not relevant	38760043	Improving the care of children with GENetic Rare di	Low KJ, Watford A,	BMJ Open	2024
243	C	Not relevant	38841148	The protocol for an observational Australian cohort	Saks DG, Bajorek I	Cereb Circ Cogn Beha	2024
244	C	Not relevant	39381601	Rapid identification of primary atopic disorders (PA	Niehues T, von Ha	Allergol Select	2024
245	C	Not relevant	38355463	A training program for improving the capacity of infe	Zhang D, Cheng Y,	BMC Med Educ	2024
246	C	Not relevant	38789985	Objectivizing issues in the diagnosis of complex rar	Faviez C, Chen X, (	BMC Med Inform Decis	2024
247	C	Not relevant	38934534	Clues for improvement of research in objective stru	Foy JP, Serresse L,	Med Educ Online	2024

248	C	Not relevant	38200263	Analysis and comparative evaluation of expedited p	Jeong H, Purja S, K	Gene Ther	2024
249	C	Not relevant	39708237	Supporting the continuous development and use of	Spivack OKC, Klei	J Community Genet	2024
250	C	Not relevant	40036380	Recent and anticipated novel drug approvals (1Q 2	Rim MH, Karas BL,	Am J Health Syst Phar	2025
251	C	Not relevant	39333302	Onasemnogene Abeparvovec Gene Therapy and Ri	Khuntha S, Prawja	Appl Health Econ Heal	2025
252	C	Not relevant	39822039	Large-scale and long-term wildlife research and mo	Bruce T, Amir Z, Al	Biol Rev Camb Philos S	2025
253	C	Not relevant	39949400	Coexistence of T-Cell Lymphoblastic Lymphoma ar	Kamış ŞÇ, Yağcı B	Case Rep Oncol Med	2025
254	C	Not relevant	39917159	Familial Glucocorticoid Deficiency Type 4 Caused b	Alquraishi AS, Albi	Cureus	2025
255	C	Not relevant	39974235	Metastatic Melanoma to Ovary and Advances in Me	Cantave M, Chen A	Cureus	2025
256	C	Not relevant	39925562	Pediatric Granulomatosis With Polyangiitis: A Case	Costin M, Cinteza	Cureus	2025
257	C	Not relevant	39974295	New Delhi Metallo-Beta-Lactamases (NDM)-Carba	Guru S, Harish V, C	Cureus	2025
258	C	Not relevant	39906419	ERCC6L2-Associated Inherited Bone Marrow Failur	Kranjcec I, Matijas	Cureus	2025
259	C	Not relevant	39901997	Awareness of Varicose Veins Associated With the U	Mawkili W, Zakri S	Cureus	2025
260	C	Not relevant	40034887	Unresectable Moderately Differentiated Gallbladde	Sabih M, Ahmad S	Cureus	2025
261	C	Not relevant	39949455	A Teen With Trisomy 18: Challenges and Triumphs (	Tate WB, Ward K, S	Cureus	2025
262	C	Not relevant	39958080	A Rare Presentation of Five Primary Cancers in a Pa	Yadav R, Raman S	Cureus	2025
263	C	Not relevant	39936420	Translational Informatics Driven Drug Repositioning	Zheng X, Chen J, Z	Curr Neuropharmacol	2025
264	C	Not relevant	39638417	Childhood interstitial lung disease survivors in adul	Manali ED, Griese	Eur Respir J	2025
265	C	Not relevant	39603287	Crystal structure of F10 core protein from Mpox viru	Zhao R, Zhu XY, Zh	Int J Biol Macromol	2025
266	C	Not relevant	40046025	Research hotspots and trends of the SLC26A4 gene	Li Y, Wen C, Yu Y, L	Intractable Rare Dis Re	2025
267	C	Not relevant	39183127	Differential times of submission and approval of CF	Costa E, Girotti S,	J Cyst Fibros	2025
268	C	Not relevant	39983748	Changing life expectancy in European countries 19	GBD 2021 Europe	Lancet Public Health	2025
269	C	Not relevant	39689660	Environmental DNA/RNA metabarcoding for noninv	Ye P, Cheng J, Lo L	Mar Pollut Bull	2025
270	C	Not relevant	40008466	The role of genetics in the prognosis of acute myoca	Tomás MJ, Pinho A	Monaldi Arch Chest Di	2025
271	C	Not relevant	39812704	Neuronal ceroid lipofuscinosis 11 (CLN11) present	Maximiano-Alves C	Neurogenetics	2025
272	C	Not relevant	38965114	AChR-seropositive myasthenia gravis in muscular d	Avallone AR, Di Ste	Neurol Sci	2025
273	C	Not relevant	39254482	A scoping review of the role of managed entry agree	García-Parra B, Gu	Amyotroph Lateral Scl	2025
274	C	Not relevant	39833864	A genomic strategy for precision medicine in rare d	Méndez-Vidal C, B	J Transl Med	2025
275	C	Not relevant	39962044	Reporting of Fairness Metrics in Clinical Risk Predic	Rountree L, Lin YT,	Online J Public Health	2025
276	C	Not relevant	39939269	Evaluation of large language models for providing e	Huang Y, Shi R, Ch	Cont Lens Anterior Eye	2025
277	C	Not relevant	40019555	Next-generation nephrology: part 2-mainstreaming	Gupta A, Jayasingh	Pediatr Nephrol	2025
